# Humanized mutant FUS drives progressive motor neuron degeneration without aggregation in ‘FUSDelta14’ knockin mice

**DOI:** 10.1093/brain/awx248

**Published:** 2017-10-07

**Authors:** Anny Devoy, Bernadett Kalmar, Michelle Stewart, Heesoon Park, Beverley Burke, Suzanna J Noy, Yushi Redhead, Jack Humphrey, Kitty Lo, Julian Jaeger, Alan Mejia Maza, Prasanth Sivakumar, Cinzia Bertolin, Gianni Soraru, Vincent Plagnol, Linda Greensmith, Abraham Acevedo Arozena, Adrian M Isaacs, Benjamin Davies, Pietro Fratta, Elizabeth M C Fisher

**Affiliations:** 1 Department of Neurodegenerative Disease, UCL Institute of Neurology, Queen Square, London WC1N 3BG, UK; 2 Sobell Department of Motor Neuroscience and Movement Disorders, UCL Institute of Neurology, Queen Square, London WC1N 3BG, UK; 3 The Mary Lyon Centre, MRC Harwell Institute, Harwell, Oxfordshire OX11 0RD, UK; 4 UCL Genetics Institute, Gower Street, London WC1E 6BT, UK; 5 Department of Neurosciences, Università degli Studi di Padova, 35121 Padova, Italy; 6 MRC Center for Neuromuscular Diseases, UCL Institute of Neurology, Queen Square, London WC1N 3BG, UK; 7 Hospital Universitario de Canarias, Fundación Canaria de Investigación Sanitaria, Tenerife, Canary Islands, Spain; 8 UK Dementia Research Institute, UCL Institute of Neurology, Queen Square, London, WC1N 3BG, UK; 9 Wellcome Trust Centre for Human Genetics, Roosevelt Drive, Oxford OX3 7BN, UK

**Keywords:** ALS, FUS, mouse, Delta14, humanization

## Abstract

Mutations in *FUS* are causative for amyotrophic lateral sclerosis with a dominant mode of inheritance. In trying to model FUS-amyotrophic lateral sclerosis (ALS) in mouse it is clear that *FUS* is dosage-sensitive and effects arise from overexpression *per se* in transgenic strains. Novel models are required that maintain physiological levels of FUS expression and that recapitulate the human disease—with progressive loss of motor neurons in heterozygous animals. Here, we describe a new humanized FUS-ALS mouse with a frameshift mutation, which fulfils both criteria: the FUS Delta14 mouse. Heterozygous animals express mutant humanized FUS protein at physiological levels and have adult onset progressive motor neuron loss and denervation of neuromuscular junctions. Additionally, we generated a novel antibody to the unique human frameshift peptide epitope, allowing specific identification of mutant FUS only. Using our new FUSDelta14 ALS mouse-antibody system we show that neurodegeneration occurs in the absence of FUS protein aggregation. FUS mislocalization increases as disease progresses, and mutant FUS accumulates at the rough endoplasmic reticulum. Further, transcriptomic analyses show progressive changes in ribosomal protein levels and mitochondrial function as early disease stages are initiated. Thus, our new physiological mouse model has provided novel insight into the early pathogenesis of FUS-ALS.

## Teaser

Devoy *et al.* develop the first mouse model to fully recapitulate human FUS-ALS, as defined by midlife-onset progressive degeneration of motor neurons with dominant inheritance. A toxic gain of function occurs in the absence of FUS protein aggregation, involving disturbance of ribosomes and mitochondria at the endoplasmic reticulum.

## Introduction

Amyotrophic lateral sclerosis (ALS) is characterized by progressive degeneration of motor neurons in the brain and spinal cord, leading to muscle atrophy, paralysis and death (Taylor *et al.*, 2016). Although the vast majority of ALS is sporadic (without a family history), ∼10% is familial; mutations in the gene *FUS* (fused-in-sarcoma) account for ∼5% of familial and ∼1% sporadic ALS (Kwiatkowski *et al.*, 2009; Vance *et al.*, 2009; Rademakers *et al.*, 2010; Nolan *et al.*, 2016). Of the >40 mutations known in this multi-domain protein, most cluster near or within the final exon, which encodes the nuclear localization signal (Dormann and Haass, 2013; Deng *et al.*, 2014; Nolan *et al.*, 2016; Svetoni *et al.*, 2016); these may be missense or truncation mutations.

FUS binds to thousands of RNAs and regulates many aspects of RNA biology (Ling *et al.*, 2013; Kapeli *et al.*, 2016; Nolan *et al.*, 2016; Shang and Huang, 2016; Svetoni *et al.*, 2016). Several mouse strains have been created to understand FUS biology although not necessarily ALS pathology (Nolan *et al.*, 2016). These include knockouts, wild-type and mutant FUS overexpressing transgenics—including Cre-inducible and partially deleted FUS alleles. These models give important insights into FUS function and have shown neurons are exquisitely sensitive to FUS gene/protein dose—thus it is unclear which aspects of neurodegeneration arise from ectopic overexpression or the effects of FUS mutation (Mitchell *et al.*, 2013; Qiu *et al.*, 2014; Nolan *et al.*, 2016).

To address this key issue, we knocked into the mouse *Fus* locus, the human ‘FUSDelta14’ truncation mutation associated with ALS onset at 20 years of age and a disease course of 22 months to death (DeJesus-Hernandez *et al.*, 2010). Heterozygous FUSDelta14 mice express mutant FUS at physiological levels, which gives rise to progressive motor neuron loss from <12 months of age in the absence of pathological aggregation. Furthermore, we generated an antibody to the unique carboxyl-terminus missense peptide that arises in this human mutation, recognizing mutant FUS only. This new powerful mouse-antibody FUS-ALS model has allowed us to identify novel early pathological changes involving ribosome and mitochondrial interactions at the endoplasmic reticulum.

## Materials and methods

All materials and methods can be found in the Supplementary material.

## Results

### FUSDelta14 knockin mice express mutant FUS with a unique frameshift C-terminus at endogenous levels

**Figure 1:**
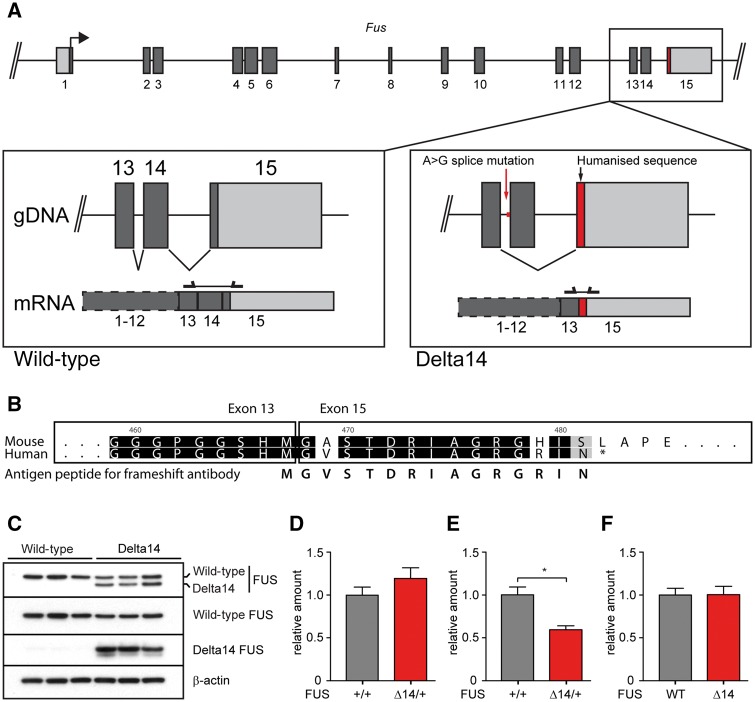
**The humanized FUS Delta14 mouse expresses endogenous levels of FUS protein.** (**A**) Schematic of the modified FUS locus. An A to G point mutation was introduced into the 5’ splice junction of exon 14 (red arrow) and the coding sequence of exon 15 was converted to the human FUS sequence (red box) to ensure production of the correct frameshifted protein, as shown in **B**. gDNA = genomic DNA. Protein coding sequence is shown in dark grey and untranslated regions are shown in light grey. (**B**) The wild-type mouse coding sequence of exon 15 does not produce the same peptide sequence as seen in human when exon 14 is skipped due to the splicing mutation; the mouse sequence lacks the early frameshift stop codon, and so would generate a 64-residue nonsense peptide, rather than the 14 residues for the human frameshift peptide. The 15-amino acid peptide used to generate a frameshift FUS-specific antibody (fsFUS) matches the human frameshift sequence. (**C**) Representative immunoblot from wild-type and heterozygous FUS Delta14 spinal cord showing endogenous levels of FUS protein. The novel frameshift-FUS antibody ‘Delta14’ specifically recognizes only FUS Delta14 protein. (**D**–**F**) The N-terminal antibody was used to quantify relative amounts of FUS in wild-type and heterozygous FUSDelta14 spinal cord. (**D**) No difference in total FUS protein was found between wild-type and heterozygous FUS Delta14 mice. (**E**) FUS Delta14 mice have approximately half as much wild-type FUS protein as their wild-type littermates (*P* = 0.0168). (**F**) The wild-type and mutant alleles in FUSDelta14 heterozygotes produce equal amounts of FUS protein. Together, these results show wild-type and heterozygous FUSDelta14 mice have equivalent endogenous levels of FUS protein.

To create a mouse model expressing mutant FUS at physiological levels, we targeted a human frameshift mutation (FUS p.G466VfsX14) (DeJesus-Hernandez *et al.*, 2010) into mouse *Fus*. The frameshift arises from an A to G point mutation in the splice acceptor site of exon 14, causing skipping of exon 14 during splicing and out-of-frame translation of exon 15 (the last exon), creating a novel frame-shifted C-terminus. We introduced the identical point mutation, g.13845A>G, into the splice acceptor site of mouse *Fus* exon 14 (Fig. 1A). The human exon 15 coding sequence was also knocked-in to ensure the frameshift peptide produced was identical to that of the human patient (14 residues long, Fig. 1A), because the mouse coding sequence lacks an early stop codon and would produce a frameshift peptide of 64 amino acids (Fig. 1B). The new strain, B6N;B6J-Fus^tm1Emcf/H^, is referred to as ‘FUSDelta14’.

We assessed protein levels in spinal cord using a panel of antibodies against wild-type and truncated frameshift FUSDelta14 proteins (Fig. 1C). An N-terminal FUS antibody recognizes both, giving a single band in wild-type and two bands in heterozygous FUSDelta14 mice. A C-terminal antibody only recognizes wild-type FUS because the epitope is lost in Delta14FUS, giving a single band in wild-type and heterozygous FUSDelta14 mice. We generated a novel frameshift FUS-specific antibody (fsFUS), to the last 15 residues of human FUSDelta14 frameshift protein (Fig. 1B), which specifically identifies mutant protein in heterozygous FUSDelta14 mice and not wild-type FUS protein.

The N-terminal antibody was used to quantify relative amounts of FUS in wild-type and heterozygous FUSDelta14 spinal cord and we found no difference in total FUS protein (Fig. 1D). However, FUSDelta14 mice have about half as much wild-type FUS protein as their wild-type littermates (*P* = 0.0168, Fig. 1E), because wild-type and mutant alleles in FUSDelta14 heterozygotes produce equal amounts of FUS protein (Fig. 1F). Thus wild-type and heterozygous FUSDelta14 mice have equivalent endogenous levels of FUS protein.

### Heterozygous FUSDelta14 mice have progressive motor degeneration

**Figure 2:**
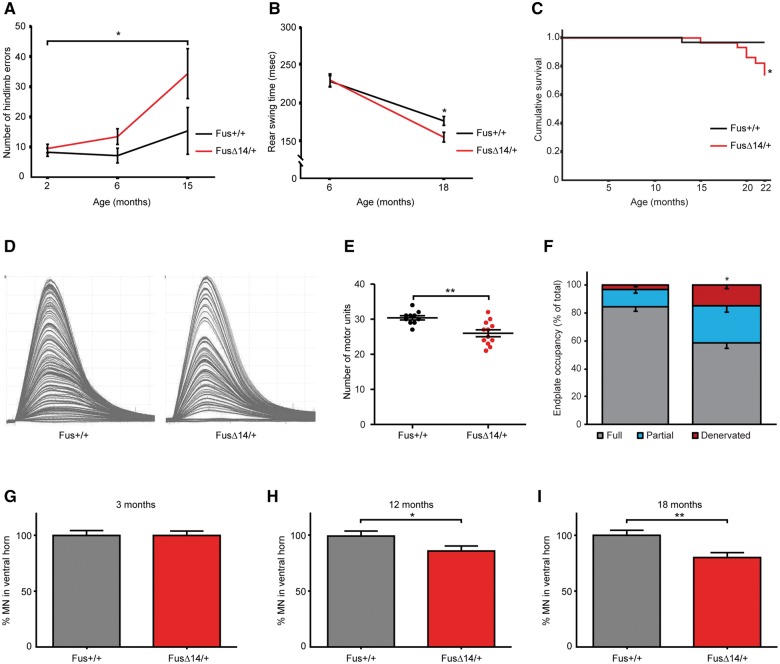
**FUS Delta14 mice develop progressive motor degeneration.** (**A**) FUS Delta14 mice show a progressive increase in hindlimb errors (missing ladder rung) during Locotronic tests (horizontal ladder). *P* = 0.039, *n* = 22 wild-type (11 males and 11 females) and 20 Delta14 mice (9 males and 11 females). (**B**) FUS Delta14 mice have a mild alteration in hindlimb gait by 18 months of age, with a reduced duration of hindlimb in swing phase. *P* = 0.021, *n* = 20 wild-type (10 males and 10 females) and 19 FUS Delta14 mice (seven males and 12 females). (**C**) Heterozygous FUS Delta14 mice show a small but significant reduction in lifespan. Kaplan-Meier LogRank (Mantel-Cox) survival, *P* = 0.033, *n* = 30 wild-type (15 males and 15 females) and 30 FUS Delta14 mice (15 males and 15 females). Graph does not include deaths from general welfare concerns associated with all inbred strains. (**D**) Examples of motor unit recording from extensor digitorum muscle of wild-type and heterozygous FUS Delta14 mice at 18 months of age. (**E**) There are significantly fewer motor units in the extensor digitorum muscle of FUS Delta14 mice compared to wild-type littermates. *P* = 0.0022, *n* = 6 per genotype, males only. (**F**) The proportion of fully innervated endplates decreased significantly in FUS Delta14 mice compared to wild-type littermates. *P* = 0.028, *n* = 6 per genotype, males only. (**G**) No motor neuron loss is observed in the L3–4 lumbar spinal cord of FUS Delta14 mice at 3 months of age, ruling out developmental differences in the lumbar motor pool; *n* = 5 per genotype. (**H**) At 12 months of age there is a 14% loss of motor neurons in the lumbar spinal cord of FUS Delta14 mice compared to wild-type littermates. *P* = 0.035, *n* = 5 wild-type and 7 FUS Delta14 mice. (**I**) By 18 months of age, 20% of motor neurons have been lost in the lumbar spinal cord of FUS Delta14 mice compared to wild-type littermates. *P* = 0.006, *n* = 6 per genotype. Scale bar = 20 µm. MN = motor neuron.

We carried out a broad phenotypic testing using the International Mouse Phenotyping Consortium pipeline and identified progressive alterations in motor function, which we assessed longitudinally using Locotronic (horizontal ladder) and gait analysis. Compared to wild-type littermates, at 3 months of age heterozygous FUSDelta14 mice did not show motor impairment on either test, ruling out a developmental phenotype (Fig. 2A and B). However, ageing FUSDelta14 heterozygotes had significant, progressively increasing, Locotronic hind-limb errors (paws slipping/missing rungs) at 12 and 15 months of age (*P* = 0.039, Fig. 2A), but no significant difference in forelimb errors or time taken to complete the task (Supplementary Fig. 1A and B). Gait analysis at 18 months showed FUSDelta14 mice have altered rear stride pattern (not length), through a reduction in the time the rear limb spent in the swing phase of the stride (*P* = 0.021; Fig. 2B and Supplementary Fig. 1C).

A Kaplan-Meier survival analysis to 22 months of age showed a modest but significant reduction in survival of FUSDelta14 mice compared to wild-type littermates, from 19 months of age [LogRank(Mantel-Cox) survival *P* = 0.033, Fig. 2C].

We investigated functional motor neurons innervating the extensor digitorum hindlimb muscles, by physiological analysis of motor units. At 18 months of age motor units were significantly reduced (15%) in FUSDelta14 extensor digitorum compared to littermates (Mann-Whitney *P* = 0.0022; Fig. 2D and E). To investigate whether neuromuscular junctions in hindlimb muscles are degenerating, we carried out a morphological assessment of endplate occupancy on hindlimb lumbrical muscles. In agreement with the motor unit analysis at 18 months, intact neuromuscular junction numbers were significantly reduced in heterozygous FUSDelta14 mice compared to wild-type littermates (58% versus 85% fully innervated, Mann-Whitney *P* = 0.028, Fig. 2F).

We counted motor neurons in lumbar spinal cord at 3, 12 and 18 months of age and found no difference between FUSDelta14 and wild-type littermates at 3 months, again ruling out a developmental phenotype (Fig. 2G). However, motor neurons were significantly decreased at 12 months (14% reduction) and 18 months (20% reduction) (*P* = 0.035 and *P* = 0.006, respectively; Fig. 2H and I), which is consistent with our motor unit analysis.

### FUSDelta14 protein mislocalization to the cytoplasm increases with disease

**Figure 3:**
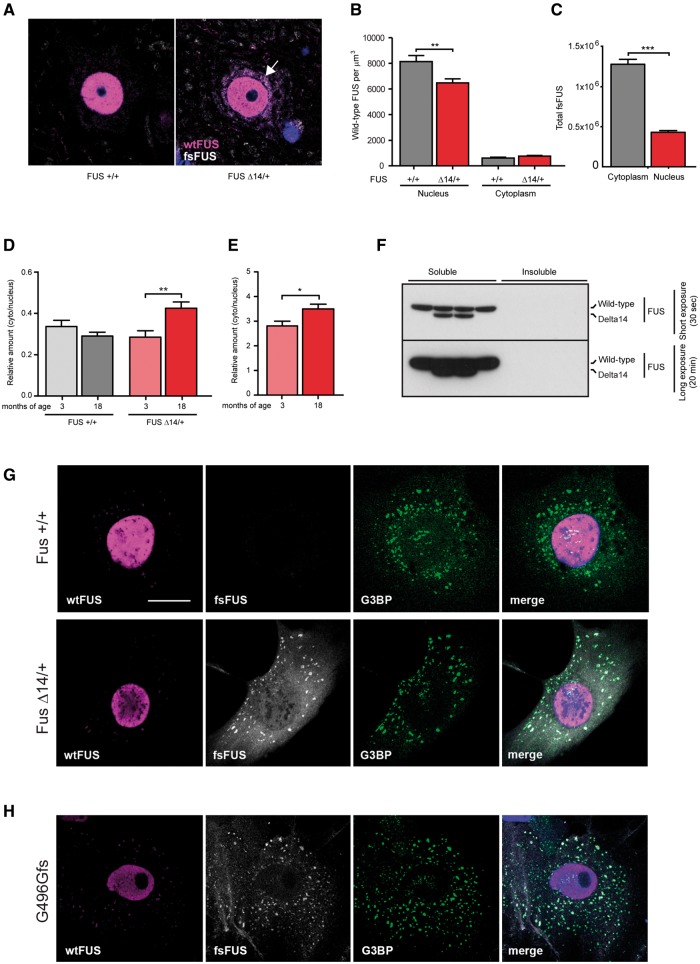
**FUS Delta14 protein mislocalizes to the cytoplasm but does not aggregate.** (**A**) Using a C-terminal FUS antibody that detects only wild-type FUS (magenta) and our novel mutant-specific FUS antibody (fsFUS, white) to quantify the distribution of FUS protein in lumbar motor neurons. Perinuclear accumulations of fsFUS are observed (arrow). (**B**) Quantification of distribution of wild-type FUS protein in the nucleus versus cytoplasm of motor neurons in wild-type versus heterozygous FUS Delta14 mice. Wild-type FUS is located almost exclusively in the nucleus in both wild-type and FUS Delta14 mice, with significantly less nuclear wild-type FUS in FUS Delta14 motor neurons (*P* = 0.0049). (**C**) Quantification of distribution of FUS Delta14 protein in the nucleus versus cytoplasm of motor neurons in heterozygous FUS Delta14 mice. There is significantly more cytoplasmic FUS Delta14 protein compared to nuclear protein, although 25% of FUS Delta14 protein is nuclear in spite of the lack of the nuclear localization signal (*P* < 0.0001). The distribution of FUS changes with age in Delta14 mice. (**D**) No differences in the cytoplasmic:nuclear ratio were observed between 3 and 18 months of age for the distribution of wild-type FUS protein in wild-type mice. However, there was a significant increase in the cytoplasmic:nuclear ratio of wild-type FUS protein in heterozygous FUS Delta14 mice (*P* = 0.0062). (**E**) There is an increase in cytoplasmic:nuclear ratio of mutant FUS protein at 18 months of age in heterozygous FUS Delta14 mice (*P* = 0.0486). (**F**) There is no insoluble FUS protein present in the lumbar spinal cord of 12-month-old wild-type and heterozygous FUS Delta14 mice. SOD1 G93A spinal cord was used as a positive control (data not shown). Mutant FUS protein is preferentially recruited into stress granules. (**G**) In wild-type and heterozygous adult mouse fibroblasts almost no wild-type FUS protein (wtFUS, magenta) is detected in cytoplasmic stress granules. However, in heterozygous FUS Delta14 fibroblasts a significant proportion of cytoplasmic frameshift FUS protein (fsFUS, white) co-localizes with the stress granule marker G3BP, green. (**H**) Human fibroblasts with a frameshift mutation in FUS (G496Gfs), which have the same frameshift peptide sequence at the C-terminus that is recognized by our novel frameshift FUS antibody (fsFUS, white), show the same recruitment of cytoplasmic frameshift FUS protein to stress granules (G3BP, green) as observed in FUS Delta14 adult mouse fibroblasts. Recruitment of wild-type FUS protein to stress granules is limited (wtFUS, magenta). Scale bar = 20 µm.

The FUSDelta14 mutation removes the nuclear localization signal. We assessed the distribution of FUS protein in lumbar spinal motor neurons by immunocytochemistry with the C-terminal FUS antibody that detects only wild-type FUS, and our novel mutant-specific fsFUS antibody (Fig. 3A, and negative controls in Supplementary Fig. 2). We found wild-type FUS is almost exclusively nuclear in wild-type and FUSDelta14 mice at 18 months of age, with significantly less nuclear wild-type FUS in FUSDelta14 motor neurons (*P* = 0.0049; Fig. 3B). We saw a trend for increased cytoplasmic wild-type FUS in FUSDelta14 motor neurons but this did not quite reach significance (*P* = 0.0512). Interestingly, our mutant-specific antibody showed that regardless of having no nuclear localization signal, ∼25% FUSDelta14 protein lies in the nucleus of FUSDelta14 motor neurons (*P* < 0.0001, Fig. 3C), while ∼75% is cytoplasmic.

No differences in cytoplasmic:nuclear ratio were observed between 3 and 18 months of age for the distribution of wild-type FUS protein in wild-type mice. However, there was a significant increase in cytoplasmic:nuclear ratio at 18 months of age for both wild-type FUS (*P* = 0.0062; Fig. 3D) and mutant FUS (*P* = 0.0486; Fig. 3E) in heterozygous FUSDelta14 mice.

### FUSDelta14 protein does not aggregate and is not depleted from nucleus

In nucleus and cytoplasm, FUSDelta14 protein distribution was predominantly diffuse with some perinuclear accumulations. We did not observe any motor neurons that had complete depletion of FUS protein from the nucleus. We found no evidence of insoluble FUS (Fig. 3F) in spinal cord lysates of 12-month-old FUSDelta14 mice (Fig. 3F)—the youngest age that showed motor neuron loss—which agrees with our histological observation of no aggregated p62 and ubiquitin pathology, beyond what would be expected in aged mice (Supplementary Fig. 3). Importantly, this suggests pathological FUS aggregates and associated nuclear depletion of FUS do not initiate disease and do not cause early motor neuron loss.

Human FUSDelta14 forms p62 positive cytoplasmic inclusions when expressed from an AAV-vector injected into adult wild-type mouse brain (Verbeeck *et al.*, 2012). This is most likely explained by the protein level, because FUS spontaneously aggregates at high concentration (Kino *et al.*, 2011; Shelkovnikova *et al.*, 2014; Murakami *et al.*, 2015). However, to rule out that lack of aggregation pathology was due to differences between the partially humanized mouse FUSDelta14 protein and human mutant FUS protein, we compared the formation of stress granules. FUS protein is recruited and accumulates in stress granules and it has been suggested that stress granules may act as a site for initiation/seeding of FUS aggregation (Shelkovnikova *et al.*, 2013; Murakami *et al.*, 2015; Yasuda *et al.*, 2017). We investigated FUS recruitment to stress granules in FUSDelta14 adult mouse fibroblasts and human primary fibroblasts from FUS-ALS patients—including a patient with a frameshift mutation generating the same nonsense peptide at the C-terminus, allowing specific detection of this protein by our fsFUS antibody. FUSDelta14 mouse fibroblasts had an identical response to low level induced stress as human patient fibroblasts (Fig. 3G, H and Supplementary Fig. 4), showing, for the first time, that when expressed at physiological levels, mutant FUS protein is preferentially recruited to stress granules, without wild-type FUS. This clearly indicates that mutant FUS acts in a gain-of-function manner with regard to stress granule formation, and that our humanized mutant FUS faithfully models human mutant FUS.

### FUSDelta14 mice have disturbed mitochondria and ribosomes

**Figure 4:**
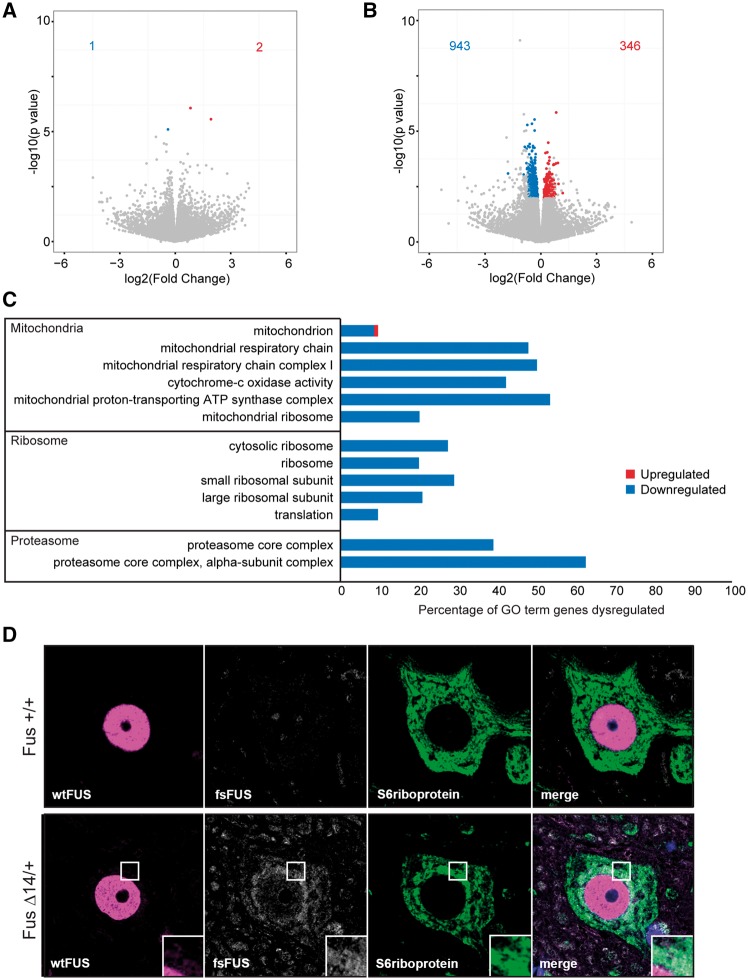
**Heterozygous FUS Delta14 mice have alteration of mitochondria and ribosomes.** (**A** and **B**) Volcano plots of differential expression in spinal cord at (**A**) 3 months and (**B**) 12 months of age. Genes with adjusted *P*-values (FDR) < 0.1 are marked in red (upregulated) and blue (downregulated). *n* = 4 per genotype. (**C**) Representative chart of GO term pathway enrichment analysis in genes dysregulated at 12 months of age in FUS Delta14 compared to wild-type littermates. Genes associated with mitochondria, ribosomes and the proteasome are significantly enriched. These genes are predominantly downregulated (blue), rather than upregulated (red). The full table of enriched GO terms with *P*-values is in Supplementary Table 1. (**D**) Using our novel mutant-specific FUS antibody that only recognizes frameshift FUS protein (fsFUS, white), we observe significant accumulation at the RER (Pearson correlation 0.415). The RER was delineated by an antibody to the ribosomal protein S6 (green). Wild-type FUS was detected with a c-terminal FUS antibody (magenta). 18 months of age, *n* = 6 per genotype.

Removal and/or mutation of FUS protein disturbs RNA metabolism, including gene expression levels. To investigate the impact of FUSDelta14 on the transcriptome we carried out longitudinal RNAseq on lumbar spinal cord. In heterozygous FUSDelta14 mice at the 3-month asymptomatic age, we identified only three genes with altered expression (Fig. 4A and Supplementary Table 1). However, by 12 months of age heterozygous FUSDelta14 mice have 1289 dysregulated genes (Fig. 4B and Supplementary Table 2), predominantly showing decreased expression. Gene ontology (GO) analysis and Gene Set Enrichment Analysis (GSEA) identified genes encoding mitochondrial proteins, ribosomal proteins and the catalytic core of the proteasome as significantly enriched in the list of dysregulated genes (Fig. 4C, Supplementary Fig. 5 and Supplementary Tables 3–10). A number of dysregulated genes were validated at the protein level. Interestingly, while gene expression was down protein levels were up (Supplementary Fig. 6), suggesting the possibility that an increase in proteins may be driving the gene downregulation.

We noticed that the perinuclear accumulations of mutant FUSDelta14 (Fig. 3A) bear a striking resemblance to Nissl substance, which stains the rough endoplasmic reticulum (RER), an area of dense accumulation of ribosomes and mitochondria. We confirmed that the cytoplasmic accumulations of FUSDelta14 protein overlap RER by co-localization with S6 riboprotein (Pearson correlation 0.415; Fig. 4D), indicating that mutant FUS may directly contribute to early ribosome and mitochondrial alterations.

## Discussion

Here we describe the first mouse model to fully recapitulate human FUS-ALS, as defined by midlife-onset (<12 months of age) progressive degeneration of motor neurons with a dominant mode of inheritance. Crucially, we express humanized mutant FUS from the endogenous mouse *Fus* locus and both mRNA and protein are expressed at endogenous levels. By behavioural analysis of motor performance, muscle physiology to assess innervation and function of hindlimb muscles, and pathological analysis of spinal motor neurons, the FUSDelta14 mouse has a consistent clinical picture of dominantly inherited, adult-onset, progressive degeneration of motor neurons.

Recently, Scekic-Zahirovic *et al.* (2017) generated heterozygous knockin mice expressing a reversible *Fus* nuclear localization signal-deletion allele, which have late-onset motor neuron loss at 22 months of age, that importantly, is rescued by selective expression of wild-type *Fus* in motor neurons. Importantly, heterozygous knock-out mice in the same study did not lose motor neurons, providing the strongest evidence to date that it is a gain-of-function through mislocalization, not loss of function that leads to motor neuron death. In contrast, our FUSDelta14 mice develop motor neuron loss considerably earlier by 12 months of age. The reason for this difference is not clear, but as FUSDelta14 mice have a normal motor system in young adulthood (3 months of age) and motor neuron loss is clearly underway by 12 months, this gives us an excellent window in which to investigate early pathomechanisms. This is an important issue for understanding ALS, as we do not know when the disease begins and have limited biomarkers for disease progression. Already, our work has shown that while pathological aggregation of FUS protein in not required for the initiation of disease and motor neuron death, there is clear evidence of disturbed proteostasis.

In FUS-ALS motor neuron loss is primarily driven by a gain-of-function mechanism (Scekic-Zahirovic *et al.*, 2016, 2017; Sharma *et al.*, 2016; Shiihashi *et al.*, 2016), and the focus has been on cytoplasmic gain-of-function because mutant FUS is mislocalized and the level of mislocalization has been linked to disease severity (Bosco *et al.*, 2010; Dormann *et al.*, 2010; Higelin *et al.*, 2016). We observe an increase in cytoplasmic mislocalization of FUS as disease progresses. However, interestingly, we do not observe nuclear depletion of FUS and using our FUSDelta14 antibody, we show that in heterozygous FUSDelta14 mice ∼25% FUSDelta14 protein is nuclear, despite the lack of a nuclear localization signal. We also observed nuclear fsFUS in both our mouse and human fibroblast lines. Thus toxic gain-of-functions may occur in both the nucleus and cytoplasm.

One clear gain-of-function that we observed was almost exclusive recruitment of mutant FUS to stress granules, using our novel mutant FUS-specific antibody, showing clearly for the first time that wild-type FUS has limited recruitment when expressed at physiological levels. Importantly, we found complete correlation between our FUSDelta14 fibroblasts and human FUS-ALS fibroblasts, highlighting that the behaviour of mutant FUS protein is consistent between mouse and human.

Our RNAseq results agree with a toxic gain-of-function: FUSDelta14 showed few alterations in gene expression at an asymptomatic age (3 months) in contrast to knockdown/knockout studies (Lagier-Tourenne *et al.*, 2012; Colombrita *et al.*, 2015). At a symptomatic time point (12 months) gene expression was dramatically dysregulated. The pathways over-represented are translation (ribosomes), energy metabolism (mitochondria) and protein turnover (proteasome) and, with protein levels also dysregulated, may highlight disturbed proteostasis as a key early disease stage. Importantly, we identified FUSDelta14 protein accumulates at the RER, which is a key location for the interaction between these pathways, suggesting a potential pathomechanism and site of action.

Our data suggest the FUS-ALS toxic gain-of-function occurs in cytoplasm and/or nucleus, in the absence of aggregation. The FUSDelta14 mouse-antibody allows us to map molecular changes longitudinally, from early development to humane end stage, in an *in vivo* physiological model, and to investigate mutant-specific interactions with our novel antibody. This system is a powerful tool and is giving new insight into early stages of ALS.

## Supplementary Material

Supplementary Figures

Supplementary Tables S1 to S3

Supplementary Tables S4 to S10

Supplementary Materials and Methods

## Abbreviation

ALS: amyotrophic lateral sclerosis
