# Supplementary material for: Humanized mutant FUS drives progressive motor neuron degeneration without aggregation in ‘FUSDelta14’ knockin mice
Source: Brain. 2017 Oct 7;140(11):2797–805. doi: 10.1093/brain/awx248 (PMC5841203; doi:10.1093/brain/awx248)

Supplementary Figure 1

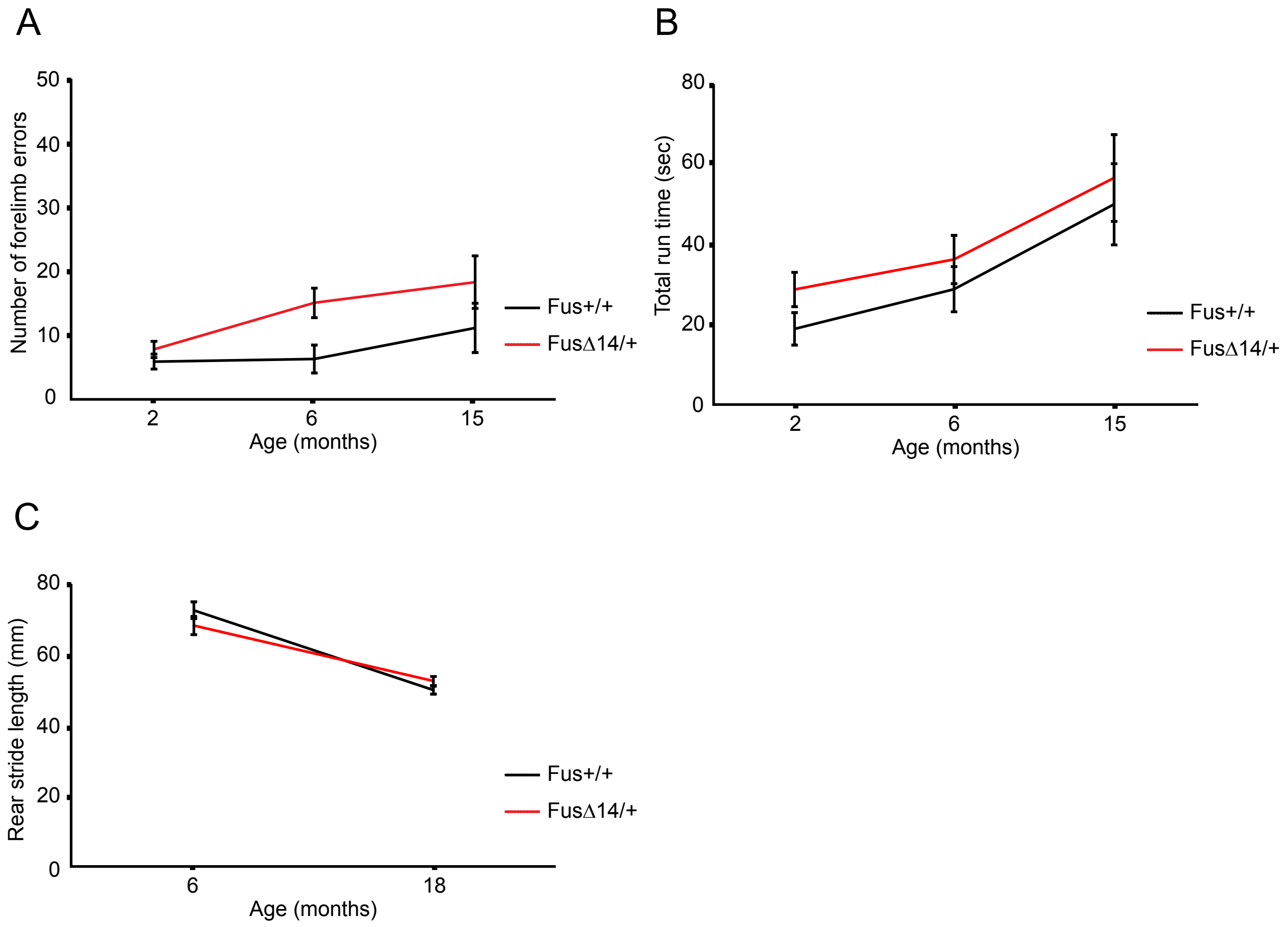

Supplementary Fig.1 No reduction in motor function in forelimbs of FUS Delta14 mice.
(A,B) As FUS Delta14 heterozygotes aged they showed no significant difference in forelimb errors **(A)** or time taken to complete the task **(B)** compared to their wildtype littermates during Locotronic tests. This is in contrast to the progressive increase in hindlimb errors observed **(Fig.2A)** **(C)** Gait analysis showed FUS Delta14 mice have no significant alteration in stride length up to 18 months of age compared to their littermates, despite having a significant change in stride pattern **(Fig.2B)**.

Supplementary Figure 2

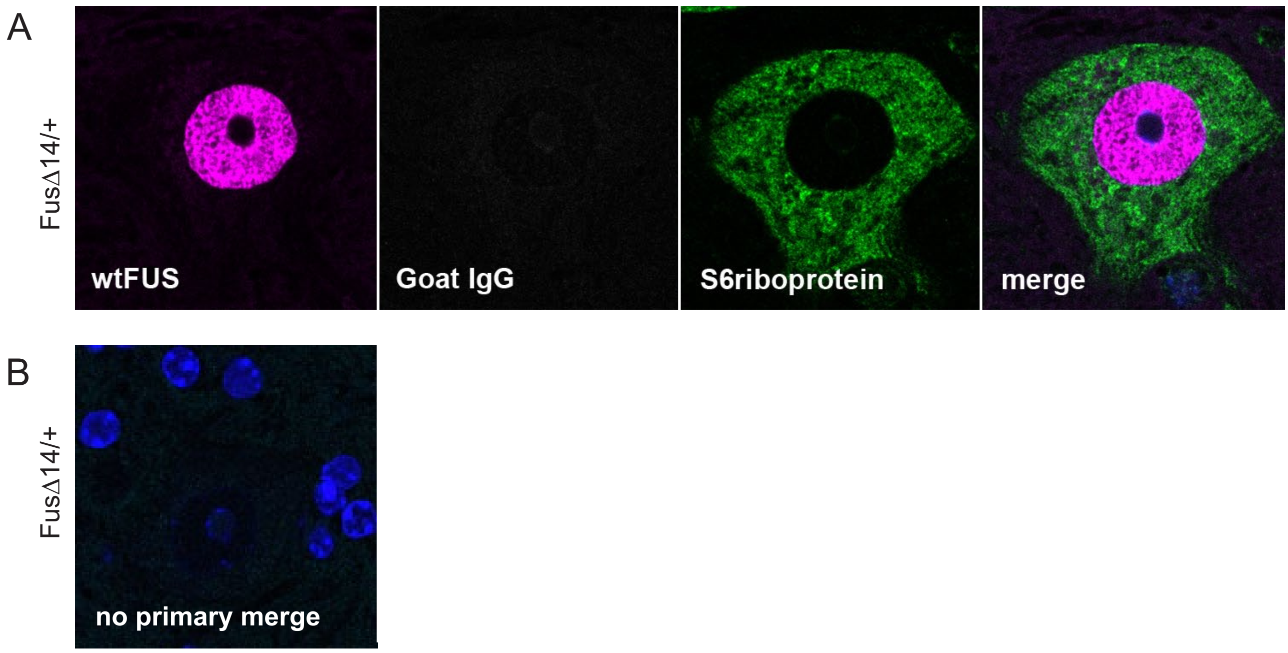

Supplementary Fig.2 Negative controls for fsFUS immunohistochemistry
(A) Goat IgG isotype control and **(B)** no primary control show that there is no non-specific binding of secondary antibodies, nor autofluorescence, contributing to the fsFUS signal shown in Figures 3A and 4D.

Supplementary Figure 3

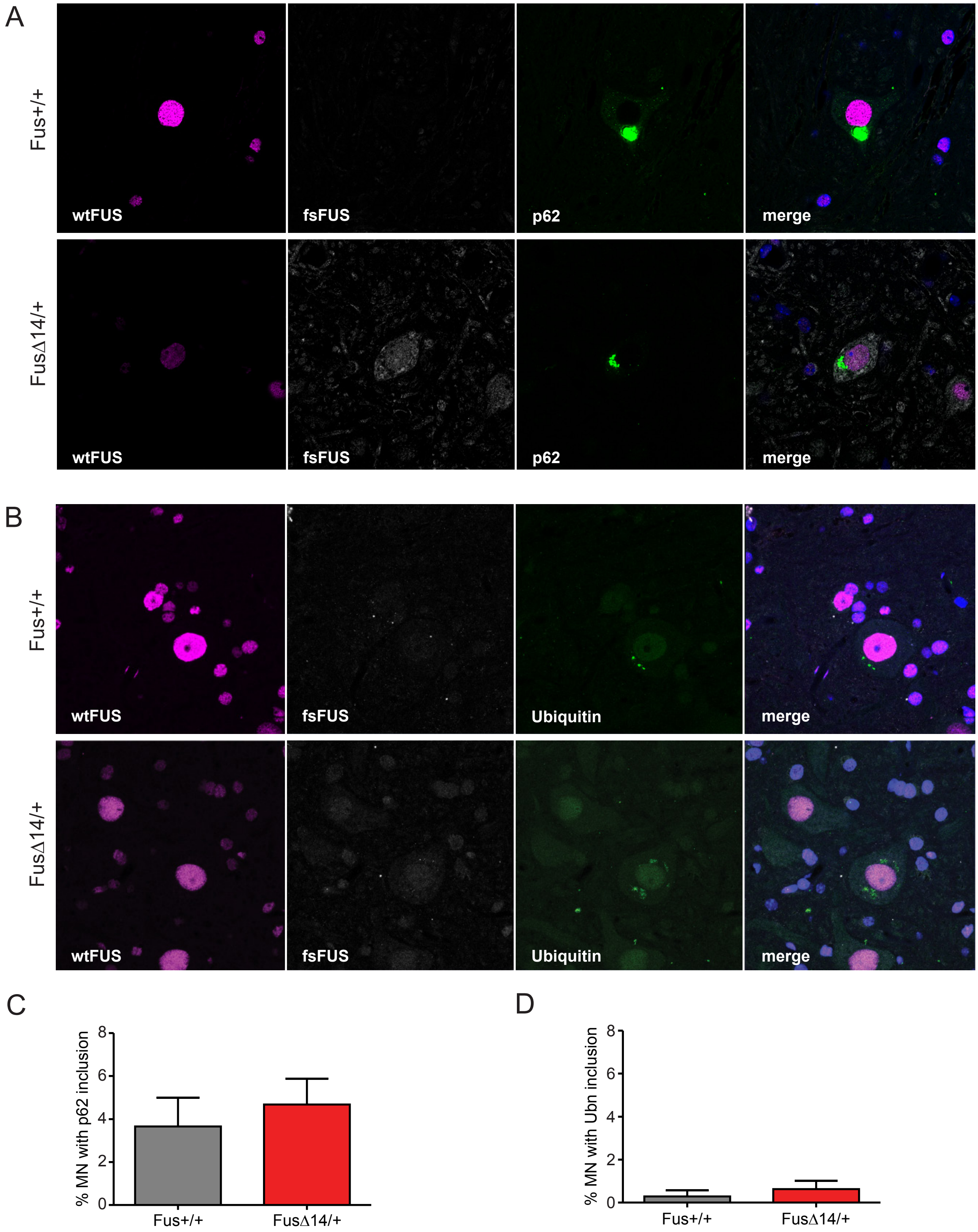

Supplementary Fig.3 p62 and ubiquitin inclusions are an age, not genotype, related phenotype. **(A, B)** Cytoplasmic p62-positive **(A)** and ubiquitin-positive **(B)** inclusions were observed in spinal motor neurons of FUS Delta14 mice at 18 months of age. However, inclusions were also present in wildtype littermates and when quantified no significant difference was found in the number of spinal motor neurons with inclusions between wildtype and FUS Delta14 mice **(C, D)**.

Supplementary Figure 4

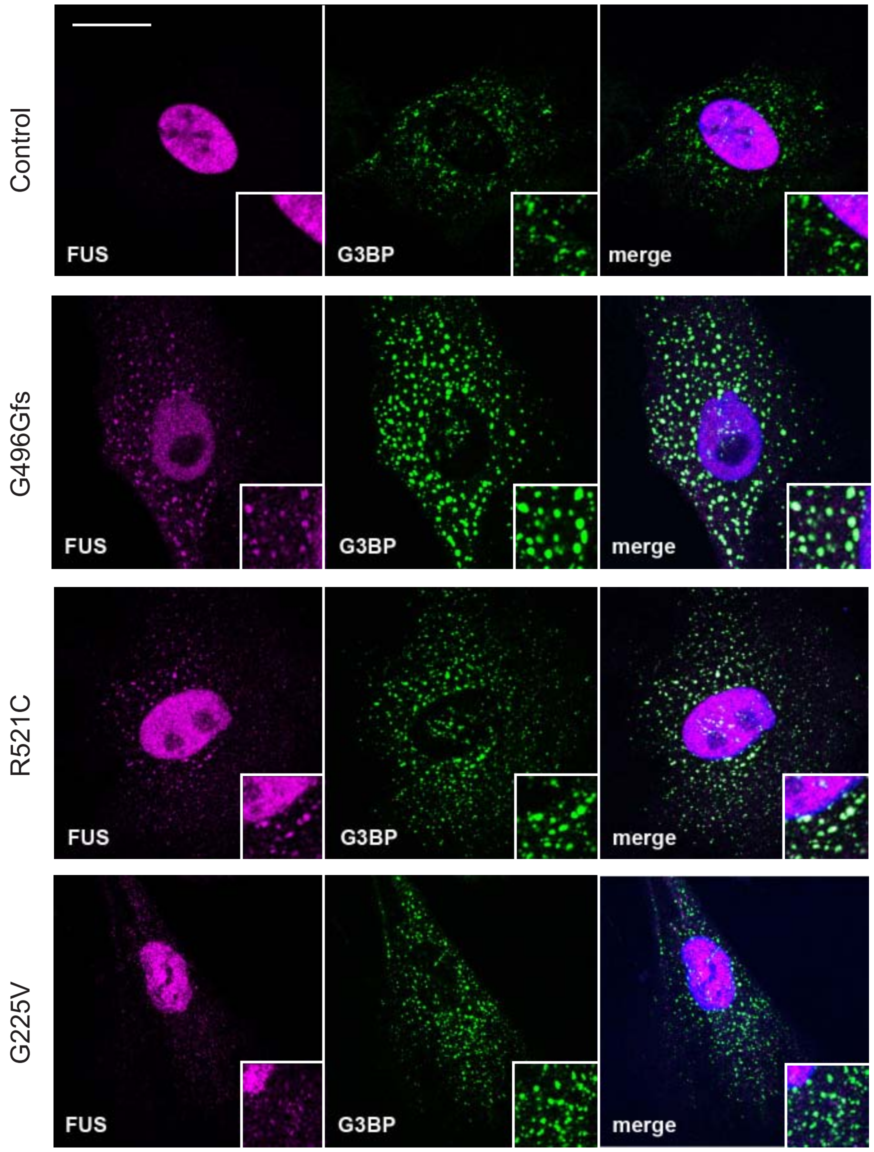

Supplementary Fig.4 Mutant FUS protein is preferentially recruited into stress granules in human fibroblasts.

In low stress conditions human fibroblast control lines (no identified mutations in ALS genes) do not recruit FUS protein (FUS, magenta) to stress granules (G3BP, green), the same as wildtype adult mouse fibroblasts (**Fig. 3G**). However, in human fibroblasts with FUS point mutations (R521C and G225V - which cannot be identified by our frameshift antibody) FUS is clearly recruited to stress granules in the same way as the human frameshift FUS line (G496Gfs) and the FUS Delta14 adult mouse fibroblasts **(Fig.3G,H)**. The FUS antibody used binds the N-terminus of FUS and recognises both wildtype and mutant FUS protein.
Scale bar 20 µm.

Supplementary Figure 5

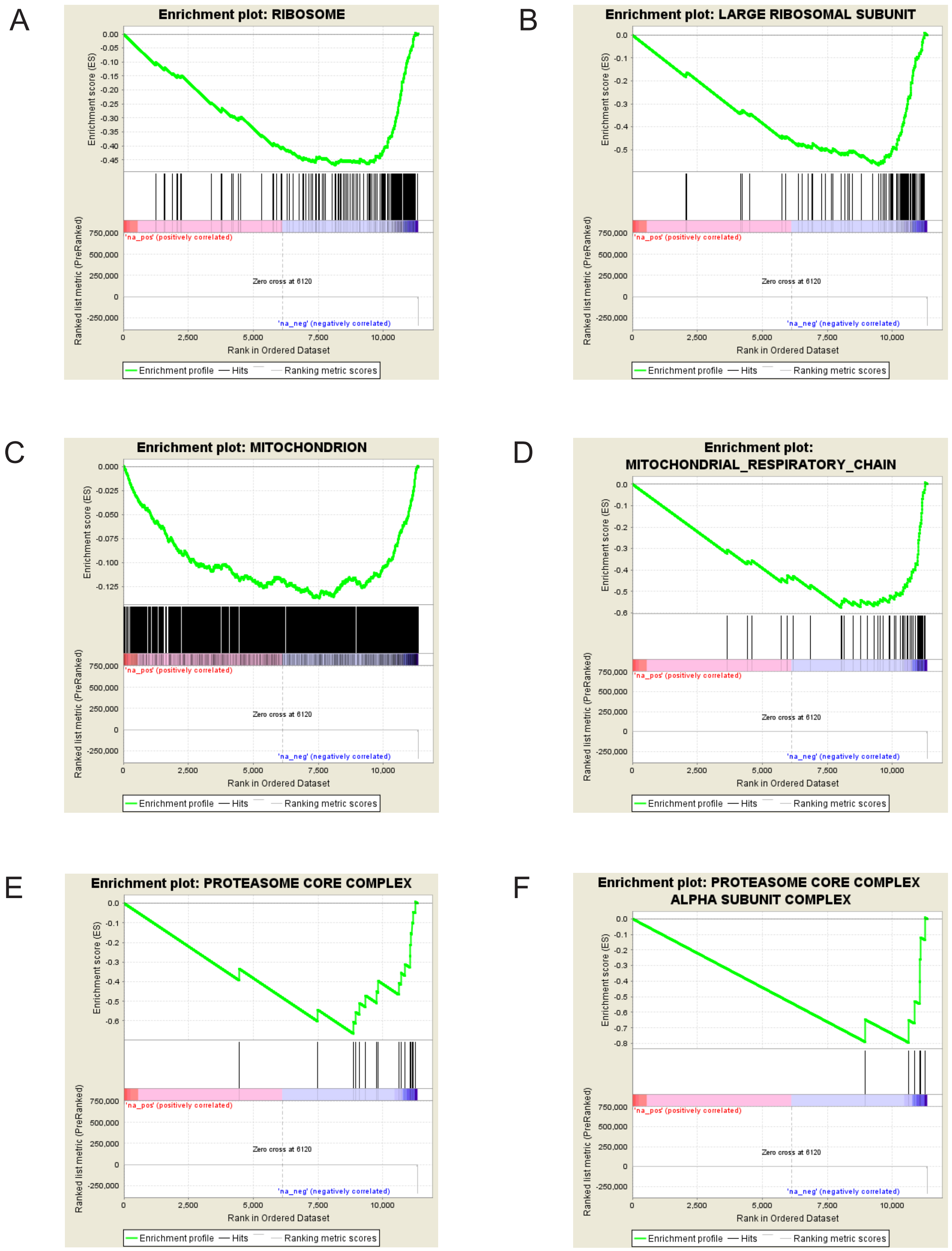

Supplementary Fig.5 Gene Set Enrichment Analysis (GSEA) further highlights the strength of enrichment for proteins associated with mitochondria, ribosome and proteasome function

Gene set enrichment analysis, unlike GO analysis, takes into account p-value, Padj and fold change for each gene. Genes associated with the ribosome **(A)** and specifically the respiratory chain **(B)** show significant enrichment in the downregulated direction in the FUS Delta14 mice at 12 months of age. Genes associated with the mitochondria **(C)** are enriched in both the up and downregulation direction, while the mitochondria respiratory chain **(D)** is enriched only in the downregulation direction. Genes of the proteasome core complex **(E)**, and specifically alpha subunits of the core complex **(F)** are enriched in the downregulation direction.

Supplementary Figure 6

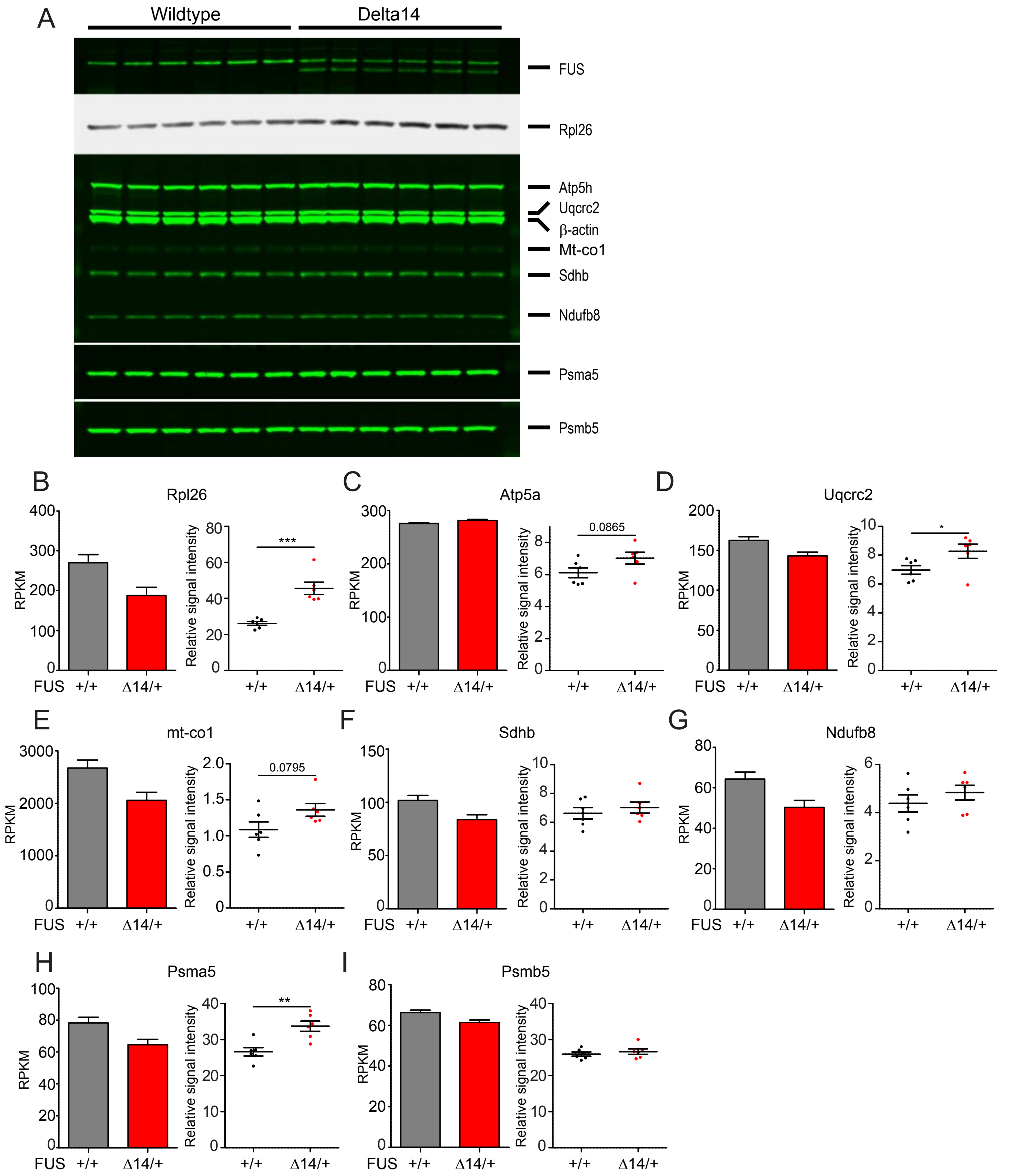

Supplementary Fig.6 Heterozygous FUS Delta14 mice have altered mitochondria, ribosomes and proteasomes. Validation of genes dyregulated at 12 months of age at the protein level shows that genes that were downregulated at RNA level (Bar Graphs) are predominantly increased in amount at the protein level (scatter plots). This pattern was true for genes associated with the ribosome large subunit (Rpl26), mitochondria respiratory chain complexes (Atp5a, Uqcrc2, mt-co1, Sdhb and NdufB8) and the proteasome catalytic core (Pmsa5 and Psmb5). Bar graphs represent RNAseq RPKM and SEM; Scatter plots represent western blot analysis of protein levels. RNAseq: n=4 per genotype; Western blot: n=6 per genotype with 2-tailed t-test used to test for significance. * = >0.05, ** = >0.01, *** = >0.001.

Supplementary Figure 7 - Original gel images from Figure 1

Fig 1 C: N-term FUS

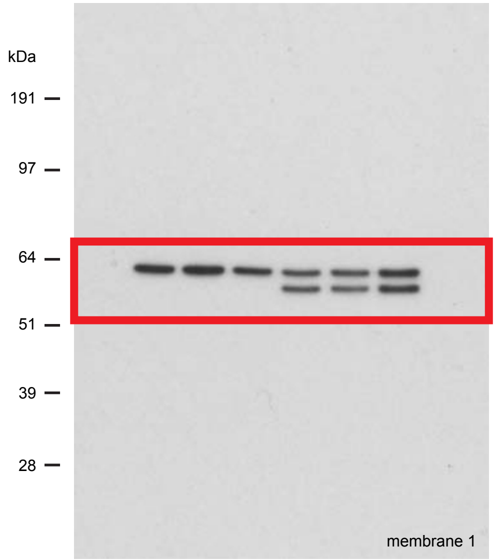

Fig 1 C: C-term FUS

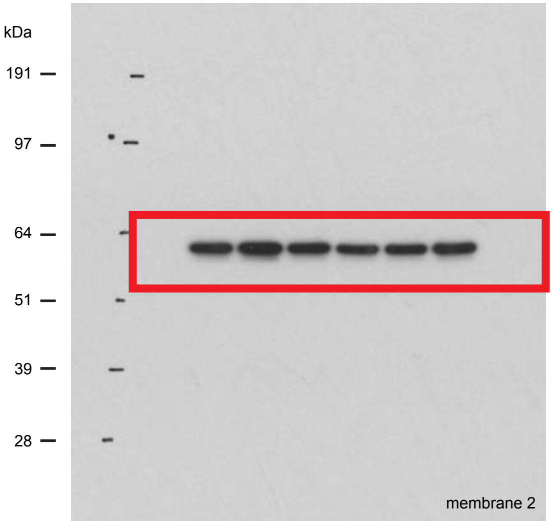

Fig 1 C: frameshift FUS

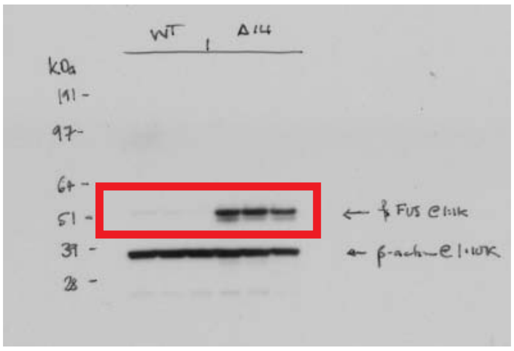

Fig 1 C: β-actin (re-probed FUS membranes 1 and 2)

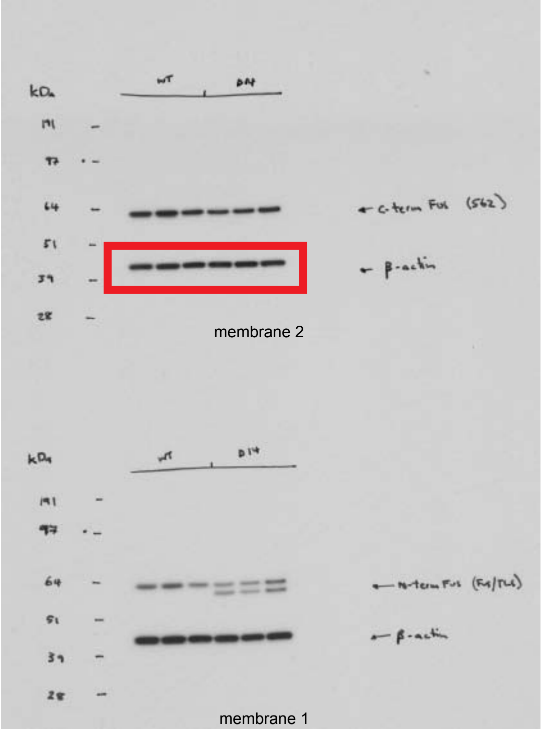

Fig 1 D-F: N-term FUS and β-actin for quantification

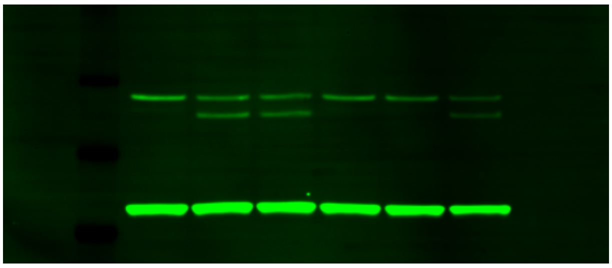

Supplementary Figure 8 - Original gel images from Supplementary Figure 6

N-term FUS

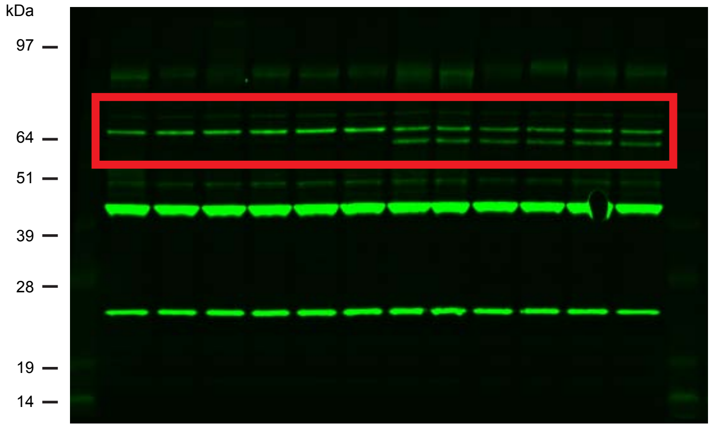

Rpl26

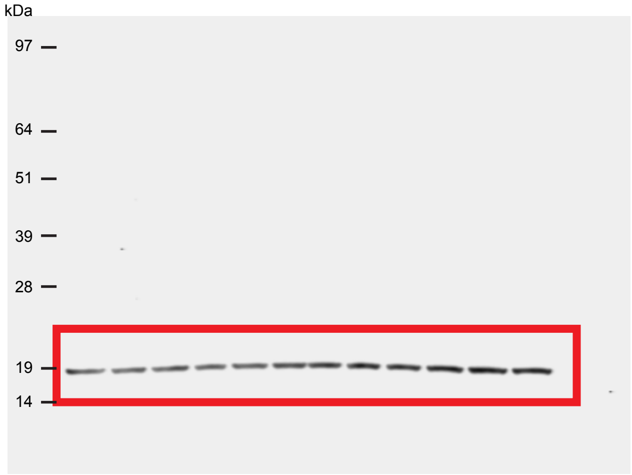

OXPHOS cocktail *

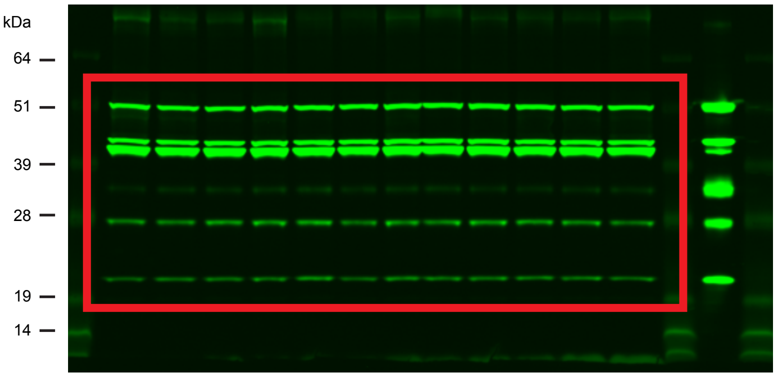

* Rat heart lysate positive control (CST)

Psma5

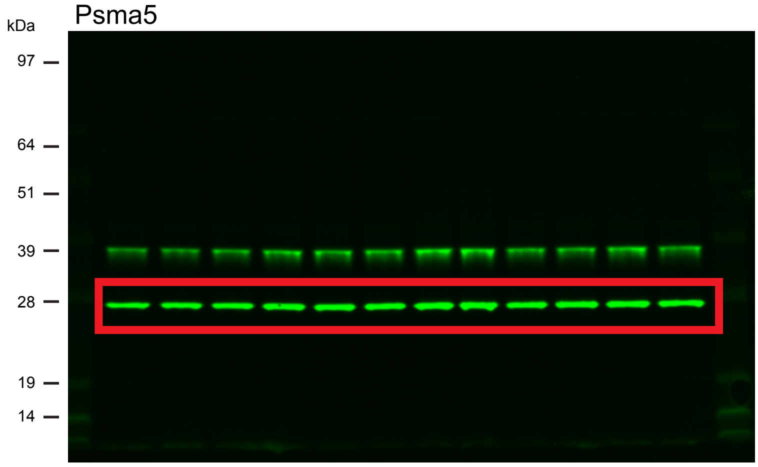

Psmb5

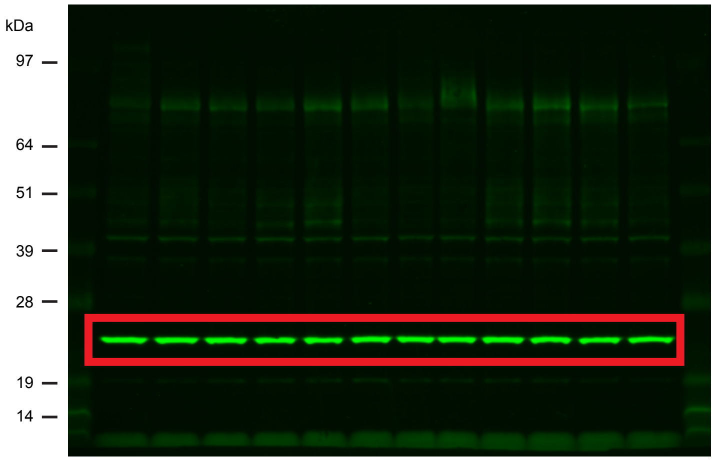

Total Protein - example of area analysed for normalisation value

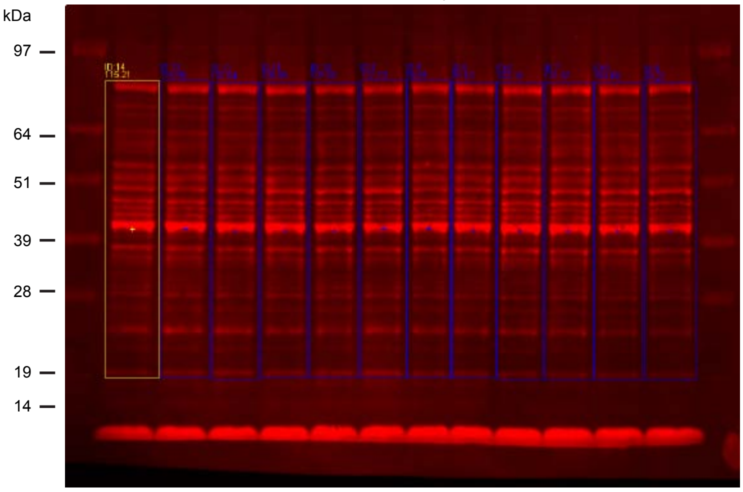

Supplement: Supplementary Figures [file brain-2017-00604-file007_awx248.pdf]
