# Supplementary material for: Humanized mutant FUS drives progressive motor neuron degeneration without aggregation in ‘FUSDelta14’ knockin mice
Source: Brain. 2017 Oct 7;140(11):2797–805. doi: 10.1093/brain/awx248 (PMC5841203; doi:10.1093/brain/awx248)
Supplement: Supplementary Tables S1 to S3 [file brain-2017-00604-file008_awx248.pdf]

Humanised mutant FUS drives progressive motor neuron degeneration without aggregation in 'FUSDelta14' knockin mice

Devoy et al. 2017

Supplementary Table 1 Expression gene list for 3 months

external_gene_id	EnsemblID	log2FoldChange	pvalue	padj
Cyp3a13	ENSMUSG00000029727	0.823002616	8.54E-07	0.025220427
Dnah14	ENSMUSG00000047369	1.932460828	2.73E-06	0.040361788
Pgm5	ENSMUSG00000041731	-0.393681834	7.96E-06	0.078360778

Humanised mutant FUS drives progressive motor neuron degeneration without aggregation in 'FUSDelta14' knockin mice

Devoy et al. 2017

Supplementary Table 2 Expression gene list for 12 months

external_gene_id	EnsemblID	log2FoldChange	pvalue	padj
Dlgap2	ENSMUSG00000047495	0.827047655	1.42E-06	0.015704761
Jkamp	ENSMUSG00000005078	-0.335386922	2.95E-06	0.015704761
1700029J07Rik	ENSMUSG00000071103	-0.4775818	4.59E-06	0.015704761
1600020E01Rik	ENSMUSG00000097048	-0.727104238	5.21E-06	0.015704761
Dtymk	ENSMUSG00000026281	-0.333959249	9.34E-06	0.022536207
Tgm2	ENSMUSG00000037820	0.401536935	3.27E-05	0.048269368
9230110C19Rik	ENSMUSG00000053070	-0.596396307	3.78E-05	0.048269368
Polr2i	ENSMUSG00000019738	-0.421369259	4.61E-05	0.048269368
Ankrd37	ENSMUSG00000050914	-0.6850316	4.75E-05	0.048269368
A330048O09Rik	ENSMUSG00000097326	-0.866495929	5.06E-05	0.048269368
Gm8104	ENSMUSG00000096446	-0.602621085	5.20E-05	0.048269368
Gm21092	ENSMUSG00000095403	-0.419030489	5.47E-05	0.048269368
Dynlrb1	ENSMUSG00000047459	-0.36872205	5.56E-05	0.048269368
Sdf2	ENSMUSG00000002064	-0.341363987	5.60E-05	0.048269368
Tdgf1	ENSMUSG00000032494	-0.764918569	6.18E-05	0.049698061
Sgk3	ENSMUSG00000025915	-0.465485865	7.50E-05	0.056117292
Caap1	ENSMUSG00000028578	-0.476904116	7.91E-05	0.056117292
Nup98	ENSMUSG00000063550	0.350901453	8.98E-05	0.060227512
Taf15	ENSMUSG00000020680	0.253288578	9.58E-05	0.060850942
Prr18	ENSMUSG00000055945	-0.277584693	0.000107648	0.063084744
Efcab1	ENSMUSG00000068617	-0.622085483	0.000109776	0.063084744
Sema3f	ENSMUSG00000034684	0.466224143	0.000150658	0.079756804
Omg	ENSMUSG00000049612	-0.296797415	0.00015298	0.079756804
Mrps22	ENSMUSG00000032459	-0.531567218	0.000158615	0.079756804
Fuom	ENSMUSG00000025466	-0.518114525	0.000169291	0.081720326
Unc50	ENSMUSG00000026111	-0.336828736	0.000200332	0.087059069
Atg10	ENSMUSG00000021619	-0.508292719	0.000202303	0.087059069
Pex13	ENSMUSG00000020283	-0.298513915	0.000206082	0.087059069
Swap70	ENSMUSG00000031015	0.456015681	0.000214186	0.087059069
Cst6	ENSMUSG00000024846	-0.480130021	0.000237783	0.087059069
Commd6	ENSMUSG00000075486	-0.524271313	0.000246107	0.087059069
Sypl	ENSMUSG00000020570	-0.325837996	0.000258842	0.087059069
Vwf	ENSMUSG00000001930	0.9173183	0.000270758	0.087059069
Gm14418	ENSMUSG00000078867	-0.77412017	0.000275572	0.087059069
Tmem208	ENSMUSG00000014856	-0.448977898	0.000276666	0.087059069
Zak	ENSMUSG00000004085	0.374123001	0.000278794	0.087059069
Tgfb3	ENSMUSG00000021253	-0.312564219	0.000280722	0.087059069
Col6a2	ENSMUSG00000020241	0.820497623	0.000283224	0.087059069
Adgrf5	ENSMUSG00000056492	0.421957161	0.000296033	0.087059069
Lamb2	ENSMUSG00000052911	0.691385157	0.000311993	0.087059069
Med21	ENSMUSG00000030291	-0.670884794	0.000313344	0.087059069
Ptpn14	ENSMUSG00000026604	0.730137878	0.000318527	0.087059069
Ntn1	ENSMUSG00000020902	0.673974339	0.000329781	0.087059069
1110059E24Rik	ENSMUSG00000035171	-0.581871756	0.000331749	0.087059069
Phospho2	ENSMUSG00000027088	-0.413743797	0.000345971	0.087059069
Mthfd2l	ENSMUSG00000029376	-0.419825715	0.000371513	0.087059069
Pantr1	ENSMUSG00000060424	-0.500022163	0.000378474	0.087059069
Tmem251	ENSMUSG00000046675	-0.405710992	0.000423628	0.087059069
Immp1l	ENSMUSG00000042670	-0.664286785	0.000427076	0.087059069
Spcs2	ENSMUSG00000035227	-0.29847896	0.000441409	0.087059069
Ostc	ENSMUSG00000041084	-0.465240713	0.000457086	0.087059069
Pfdn4	ENSMUSG00000052033	-0.733721311	0.000459831	0.087059069
Gm14403	ENSMUSG00000094786	-0.654163806	0.000468519	0.087059069

Thrsp	ENSMUSG00000035686	-0.302031619	0.000487554	0.087059069
Dpy19l3	ENSMUSG00000043671	0.301082253	0.0004885	0.087059069
Ube2w	ENSMUSG00000025939	-0.437958574	0.000493862	0.087059069
Cd164l2	ENSMUSG00000028865	-0.524788857	0.000497444	0.087059069
Mtrf1	ENSMUSG00000022022	-0.316774167	0.000501846	0.087059069
Ttpa	ENSMUSG00000073988	-0.745408502	0.000509063	0.087059069
Ugt8a	ENSMUSG00000032854	-0.296564049	0.000511472	0.087059069
Gm37111	ENSMUSG00000104494	-0.514367263	0.00052409	0.087059069
Gm1673	ENSMUSG00000070858	-0.423704535	0.000531956	0.087059069
Yars2	ENSMUSG00000022792	-0.290347519	0.000538008	0.087059069
Mir21a+Vmp1	ENSMUSG00000065455+ENSMUSG00000018171	-0.296010031	0.000540407	0.087059069
Ap4s1	ENSMUSG00000020955	-0.366180755	0.000556916	0.087059069
Commd3	ENSMUSG00000051154	-0.539666901	0.000565719	0.087059069
Serpinb1a	ENSMUSG00000044734	-0.482932323	0.000566188	0.087059069
S100a10	ENSMUSG00000041959	-0.596930802	0.000585003	0.087059069
Gm13340	ENSMUSG00000083563	-0.620754376	0.000610056	0.087059069
Ermn	ENSMUSG00000026830	-0.451423953	0.000612401	0.087059069
Cd24a	ENSMUSG00000047139	-0.439855895	0.000623547	0.087059069
Snx16	ENSMUSG00000027534	-0.374441683	0.000630224	0.087059069
Slc10a7	ENSMUSG00000031684	-0.326409401	0.000640915	0.087059069
Styx	ENSMUSG00000053205	-0.656236977	0.000647963	0.087059069
Tefm	ENSMUSG00000046909	-0.565013047	0.000653387	0.087059069
Higd1b	ENSMUSG00000020928	-0.621467006	0.000653557	0.087059069
Fam151b	ENSMUSG00000034334	-0.464249608	0.000654688	0.087059069
Atp5s	ENSMUSG00000054894	-0.3769785	0.000683253	0.087059069
Myof	ENSMUSG00000048612	0.463429057	0.000684394	0.087059069
Atp5f1	ENSMUSG00000000563	-0.341628373	0.000686286	0.087059069
Lgals4	ENSMUSG00000053964	-0.542222841	0.000715744	0.087059069
Uts2b	ENSMUSG00000056423	-0.669876305	0.000737245	0.087059069
Phf13	ENSMUSG00000047777	0.474920095	0.000739895	0.087059069
Mt2	ENSMUSG00000031762	-0.610871203	0.000740504	0.087059069
Etv1	ENSMUSG00000004151	-0.434050397	0.000754998	0.087059069
Gng13	ENSMUSG00000025739	-0.647085946	0.000759993	0.087059069
Erbb3	ENSMUSG00000018166	0.453540811	0.000762784	0.087059069
Serpinb6a	ENSMUSG00000060147	-0.291463243	0.000768209	0.087059069
Ccnh	ENSMUSG00000021548	-0.358054731	0.000771549	0.087059069
Hint2	ENSMUSG00000028470	-0.52802534	0.000774484	0.087059069
AI413582	ENSMUSG00000062753	-0.40947405	0.000785096	0.087059069
Erh	ENSMUSG00000021131	-0.478543555	0.000789541	0.087059069
mt-Co3+mt-Atp6+mt-Atp8	ENSMUSG00000064358+ENSMUSG00000064356+E	-1.759771123	0.000805989	0.087059069
Ssu72	ENSMUSG00000029038	-0.401537688	0.000820328	0.087059069
Slc5a6	ENSMUSG00000006641	0.350009195	0.000847228	0.087059069
Cst3	ENSMUSG00000027447	-0.390937328	0.000862104	0.087059069
Itgb3	ENSMUSG00000020689	0.610857232	0.000865716	0.087059069
Cldn10	ENSMUSG00000022132	-0.378961601	0.000868893	0.087059069
Polr3b	ENSMUSG00000034453	0.343176531	0.000873125	0.087059069
Col15a1	ENSMUSG00000028339	0.426256178	0.000874387	0.087059069
Ugp2	ENSMUSG00000001891	-0.378339158	0.00087599	0.087059069
Fus	ENSMUSG00000030795	0.325253125	0.000876316	0.087059069
Crnde	ENSMUSG00000031736	-0.656954828	0.000877184	0.087059069
Adgra2	ENSMUSG00000031486	0.563685103	0.000882307	0.087059069
Mir16-1+Dleu2	ENSMUSG00000097589+ENSMUSG00000092995	-0.591374848	0.000898558	0.087059069
Ebpl	ENSMUSG00000021928	-0.546967498	0.000899956	0.087059069
Tmem198	ENSMUSG00000051703	0.359043089	0.000904367	0.087059069
Dnmbp	ENSMUSG00000025195	0.464998625	0.000907371	0.087059069
mt-Nd6	ENSMUSG00000064368	-0.91855042	0.00090774	0.087059069
Serpinb9	ENSMUSG00000045827	-0.462821504	0.00090818	0.087059069
Dbi	ENSMUSG00000026385	-0.446459408	0.000923056	0.087059069
2610001J05Rik	ENSMUSG00000052419	-0.551314747	0.000928459	0.087059069

Dars	ENSMUSG00000026356	-0.368670062	0.00094784	0.087059069
Zfp382	ENSMUSG00000074220	0.395687614	0.000998213	0.087059069
Trappc2l	ENSMUSG00000015013	-0.464978843	0.001020161	0.087059069
Pam16	ENSMUSG00000014301	-0.420922024	0.001025943	0.087059069
Ndufv2	ENSMUSG00000024099	-0.53490767	0.001029709	0.087059069
Med10	ENSMUSG00000021598	-0.34205846	0.001032692	0.087059069
Prdx3	ENSMUSG00000024997	-0.271105299	0.001045059	0.087059069
Tmem256	ENSMUSG00000070394	-0.586126642	0.001046356	0.087059069
Crtap	ENSMUSG00000032431	0.452306918	0.001054338	0.087059069
Psma1	ENSMUSG00000030751	-0.272781944	0.001057878	0.087059069
Nedd8	ENSMUSG00000010376	-0.516368558	0.001066991	0.087059069
Msmo1	ENSMUSG00000031604	-0.264692985	0.001073229	0.087059069
Uqcr10	ENSMUSG00000059534	-0.496106351	0.001079869	0.087059069
Hopx	ENSMUSG00000059325	-0.480853366	0.001082497	0.087059069
Tmem205	ENSMUSG00000040883	-0.44965504	0.001099727	0.087059069
Txndc17	ENSMUSG00000020803	-0.508035191	0.001105671	0.087059069
Tvp23b	ENSMUSG00000014177	-0.410852857	0.001107763	0.087059069
Ndufa3	ENSMUSG00000035674	-0.459806916	0.001120987	0.087059069
Gramd4	ENSMUSG00000035900	0.301439808	0.001128953	0.087059069
Fopnl	ENSMUSG00000022677	-0.521765002	0.00113067	0.087059069
Pip4k2b	ENSMUSG00000018547	0.299289981	0.001133988	0.087059069
Abcb1a	ENSMUSG00000040584	0.41800785	0.001135002	0.087059069
Bnip3	ENSMUSG00000078566	-0.267627962	0.001140469	0.087059069
Bet1	ENSMUSG00000032757	-0.51652558	0.001143455	0.087059069
Dynll1	ENSMUSG00000009013	-0.459982022	0.001145043	0.087059069
Svbp	ENSMUSG00000028643	-0.589196755	0.001146715	0.087059069
Nop10	ENSMUSG00000027133	-0.510139184	0.001154201	0.087059069
Nt5c1a	ENSMUSG00000054958	0.4619679	0.001156115	0.087059069
Chac2	ENSMUSG00000020309	-0.438521815	0.001165331	0.087059069
Smurf1	ENSMUSG00000038780	0.481772533	0.001178126	0.087059069
Gm14295	ENSMUSG00000078877	-0.663464582	0.001179893	0.087059069
Higd1a	ENSMUSG00000038412	-0.277270747	0.001185427	0.087059069
Mterf3	ENSMUSG00000021519	-0.352778037	0.001200031	0.087059069
Zfp277	ENSMUSG00000055917	-0.391648682	0.001200144	0.087059069
Mrpl1	ENSMUSG00000029486	-0.471754843	0.001202889	0.087059069
Supt4a	ENSMUSG00000020485	-0.327542658	0.001223873	0.087059069
Mrpl36	ENSMUSG00000021607	-0.338465954	0.001232897	0.087059069
Mdh1	ENSMUSG00000020321	-0.390543959	0.001233197	0.087059069
Atp6v0e	ENSMUSG00000015575	-0.532957896	0.00125309	0.087059069
Lsm3	ENSMUSG00000034192	-0.576491875	0.001253943	0.087059069
Chmp5	ENSMUSG00000028419	-0.447729078	0.001257591	0.087059069
Mpc1	ENSMUSG00000023861	-0.509917379	0.00126868	0.087059069
Rpe	ENSMUSG00000026005	-0.499414547	0.001288216	0.087059069
Snrnp48	ENSMUSG00000021431	-0.390724617	0.001304264	0.087059069
Echdc1	ENSMUSG00000019883	-0.2627989	0.001336976	0.087059069
Zfp994	ENSMUSG00000096433	-0.601206364	0.001340528	0.087059069
Med6	ENSMUSG00000002679	-0.471084484	0.00135912	0.087059069
Cd300c2	ENSMUSG00000044811	-0.528984589	0.001364066	0.087059069
Cox14	ENSMUSG00000023020	-0.425611398	0.001371449	0.087059069
Nr4a1	ENSMUSG00000023034	0.539005083	0.00137432	0.087059069
Chchd2	ENSMUSG00000070493	-0.497911346	0.001376027	0.087059069
Gpx4	ENSMUSG00000075706	-0.523881285	0.001380938	0.087059069
Myeov2	ENSMUSG00000073616	-0.606960825	0.001382006	0.087059069
Hsp90aa1	ENSMUSG00000021270	-0.221495904	0.001384239	0.087059069
Me2	ENSMUSG00000024556	0.241711294	0.001400156	0.087059069
Sucla2	ENSMUSG00000022110	-0.322765992	0.001407409	0.087059069
2010107E04Rik	ENSMUSG00000021290	-0.582994247	0.001419784	0.087059069
1500011K16Rik	ENSMUSG00000051319	-0.695388746	0.001420848	0.087059069
Camkk2	ENSMUSG00000029471	0.380330104	0.001422344	0.087059069

4930506C21Rik	ENSMUSG00000087478	-0.563020094	0.001422493	0.087059069
Mtx2	ENSMUSG00000027099	-0.483068946	0.001423551	0.087059069
Plekha7	ENSMUSG00000045659	0.420588186	0.00142598	0.087059069
Ramp1	ENSMUSG00000034353	-0.451472228	0.001432285	0.087059069
Zfp516	ENSMUSG00000058881	0.35102793	0.001444181	0.087059069
Pex7	ENSMUSG00000020003	-0.238070063	0.00145108	0.087059069
4932411E22Rik	ENSMUSG00000047773	0.470936448	0.001456213	0.087059069
Scaf11	ENSMUSG00000033228	0.179195491	0.001458311	0.087059069
Ifrd1	ENSMUSG00000001627	-0.231637465	0.001482334	0.087059069
Calcb	ENSMUSG00000030666	-0.628247022	0.001508279	0.087059069
Top3a	ENSMUSG00000002814	0.445172676	0.001509054	0.087059069
Smpd3	ENSMUSG00000031906	0.369026866	0.001510341	0.087059069
Atp8a2	ENSMUSG00000021983	0.627537373	0.001513148	0.087059069
Grp	ENSMUSG00000024517	-0.433622776	0.001514476	0.087059069
Mog	ENSMUSG00000076439	-0.225517377	0.001514828	0.087059069
Usmg5	ENSMUSG00000071528	-0.563433554	0.001515564	0.087059069
Fth1	ENSMUSG00000024661	-0.335713562	0.001516895	0.087059069
Pvalb	ENSMUSG00000005716	-0.57200341	0.001524402	0.087059069
H3f3a	ENSMUSG00000060743	-0.426200629	0.001532727	0.087059069
Echdc2	ENSMUSG00000028601	-0.279854293	0.00153518	0.087059069
Uqcc2	ENSMUSG00000024208	-0.548253761	0.00153712	0.087059069
Taf13	ENSMUSG00000048100	-0.487025835	0.001538219	0.087059069
mt-Tw+mt-Nd2	ENSMUSG00000064346+ENSMUSG00000064345	-0.440535087	0.001563892	0.087059069
	ENSMUSG00000029333	0.255216475	0.001591081	0.087059069
Coq2	ENSMUSG00000029319	-0.327848944	0.001594142	0.087059069
Fam162a	ENSMUSG00000003955	-0.529831615	0.001599801	0.087059069
Sugct	ENSMUSG00000055137	-0.379401588	0.001603825	0.087059069
Ddx42	ENSMUSG00000020705	0.198095587	0.001603829	0.087059069
Rnf13	ENSMUSG00000036503	-0.427160018	0.001604043	0.087059069
Jrkl	ENSMUSG00000079083	-0.497367697	0.001605267	0.087059069
Slc24a3	ENSMUSG00000063873	0.286152136	0.001606639	0.087059069
Grik3	ENSMUSG00000001985	0.688054336	0.001607595	0.087059069
Card10	ENSMUSG00000033170	0.339913404	0.001615143	0.087059069
Cep350	ENSMUSG00000033671	0.244902462	0.001617237	0.087059069
Tmem2	ENSMUSG00000024754	0.486306529	0.001624305	0.087059069
1810043H04Rik	ENSMUSG00000078572	-0.605122112	0.001624522	0.087059069
Mrpl48	ENSMUSG00000030706	-0.319865634	0.001631083	0.087059069
Cox8a	ENSMUSG00000035885	-0.310670788	0.001641072	0.087059069
Bdh2	ENSMUSG00000028167	-0.474427262	0.001643747	0.087059069
Plekhg2	ENSMUSG00000037552	0.51081429	0.00164601	0.087059069
Rnf7	ENSMUSG00000051234	-0.418089612	0.001646395	0.087059069
Dgkg	ENSMUSG00000022861	0.356634136	0.001658703	0.087059069
Sdcbp	ENSMUSG00000028249	-0.391888313	0.001670816	0.087059069
Glra1	ENSMUSG00000000263	0.661404184	0.001688663	0.087059069
Nf1	ENSMUSG00000020716	0.338283942	0.001690673	0.087059069
Pdcd5	ENSMUSG00000030417	-0.529330739	0.001701005	0.087059069
Fpgt	ENSMUSG00000053870	-0.370504332	0.001705224	0.087059069
2610021A01Rik	ENSMUSG00000091474	-0.391976868	0.001712167	0.087059069
Rps29	ENSMUSG00000034892	-0.505178314	0.001713774	0.087059069
Pfkfb4	ENSMUSG00000025648	0.329199509	0.001727608	0.087059069
Ccdc85c	ENSMUSG00000084883	0.251990673	0.001727832	0.087059069
Pfdn1	ENSMUSG00000024346	-0.520216668	0.001742102	0.087059069
Cnbp	ENSMUSG00000030057	-0.338360842	0.001744078	0.087059069
Timm8b	ENSMUSG00000039016	-0.590725185	0.001746936	0.087059069
Psmb7	ENSMUSG00000026750	-0.406451802	0.001754949	0.087059069
Idi1	ENSMUSG00000058258	-0.387609086	0.001758826	0.087059069
Zscan20	ENSMUSG00000061894	0.538954473	0.001770971	0.087059069
Gm27505+Gm27533+Gm28	ENSMUSG00000098543+ENSMUSG00000092274+E	-0.275028983	0.00178969	0.087059069
Tex30+Kdelc1+Gm29247	ENSMUSG00000026047+ENSMUSG00000026049+E	-0.327538737	0.001793769	0.087059069

Nsmce1	ENSMUSG00000030750	-0.353473924	0.001795527	0.087059069
Lamc1	ENSMUSG00000026478	0.555501213	0.001812509	0.087059069
Gstm5	ENSMUSG00000004032	-0.523626261	0.001814382	0.087059069
Fbn1	ENSMUSG00000027204	0.626775075	0.001815801	0.087059069
Mgst1	ENSMUSG00000008540	-0.653729871	0.001818154	0.087059069
Mrpl52	ENSMUSG00000010406	-0.451348494	0.001828206	0.087059069
Cox7a2	ENSMUSG00000032330	-0.608345191	0.001843997	0.087059069
Tmem258	ENSMUSG00000036372	-0.543863168	0.001851466	0.087059069
Gtf2b	ENSMUSG00000028271	-0.418916238	0.001852185	0.087059069
Ticam2+Tmed7	ENSMUSG00000033184+ENSMUSG00000056130	-0.455752198	0.0018556	0.087059069
A930005H10Rik	ENSMUSG00000054426	-0.297148513	0.001856061	0.087059069
Cops5	ENSMUSG00000025917	-0.322764721	0.001860448	0.087059069
Coa6	ENSMUSG00000051671	-0.54997013	0.001868803	0.087059069
Adk	ENSMUSG00000039197	-0.34849863	0.001872876	0.087059069
Nit2	ENSMUSG00000022751	-0.399801357	0.001873231	0.087059069
1110032A03Rik	ENSMUSG00000037971	-0.417583341	0.001877022	0.087059069
Gabrg1	ENSMUSG00000001260	-0.603413226	0.001886279	0.087059069
Mns1	ENSMUSG00000032221	-0.451562609	0.001915827	0.087059069
Cep162	ENSMUSG00000056919	0.211146057	0.001919047	0.087059069
Pdgfd	ENSMUSG00000032006	-0.378954424	0.001942918	0.087059069
Atraid	ENSMUSG00000013622	-0.256870843	0.001953828	0.087059069
Amn1	ENSMUSG00000068250	-0.554032344	0.001956575	0.087059069
Mrpl13	ENSMUSG00000022370	-0.429753559	0.001956896	0.087059069
Spryd7	ENSMUSG00000021930	-0.426399021	0.001957397	0.087059069
Atp6v1d	ENSMUSG00000021114	-0.252066186	0.001958435	0.087059069
mt-Rnr1	ENSMUSG00000064337	-0.508232579	0.001962074	0.087059069
Nol4l	ENSMUSG00000061411	0.498926988	0.001968347	0.087059069
Gtf2ird1	ENSMUSG00000023079	0.481803922	0.001972218	0.087059069
Ndufs4	ENSMUSG00000021764	-0.611455834	0.001991828	0.087059069
Tyw5	ENSMUSG00000048495	-0.523558773	0.001999331	0.087059069
Msantd4	ENSMUSG00000041124	-0.206234516	0.002021398	0.087059069
Fcf1	ENSMUSG00000021243	-0.542588624	0.002045689	0.087059069
Postn	ENSMUSG00000027750	-0.58897619	0.002047996	0.087059069
Cenpq	ENSMUSG00000023919	-0.593074048	0.002050598	0.087059069
Gm16534+Fam78a	ENSMUSG00000090157+ENSMUSG00000050592	0.506202424	0.002052742	0.087059069
Rpl22l1	ENSMUSG00000039221	-0.660200427	0.002052893	0.087059069
Mrpl30	ENSMUSG00000026087	-0.528680259	0.002061627	0.087059069
Ttc33	ENSMUSG00000022151	-0.341058469	0.002088546	0.087059069
Ndufc2	ENSMUSG00000030647	-0.57161794	0.002100388	0.087059069
Gng10+Gm20503+Dnajc25	ENSMUSG00000038607+ENSMUSG00000070972+E	-0.496205161	0.002102681	0.087059069
Eif3m	ENSMUSG00000027170	-0.567578107	0.002102792	0.087059069
Fam96b	ENSMUSG00000031879	-0.369814294	0.002113641	0.087059069
Rps26	ENSMUSG00000025362	-0.450574002	0.002129534	0.087059069
Atpif1	ENSMUSG00000054428	-0.509640856	0.002139207	0.087059069
Fam213b	ENSMUSG00000029059	-0.534313486	0.002145595	0.087059069
Gm27941+Crot	ENSMUSG00000003623+ENSMUSG00000099209	-0.291903175	0.002157638	0.087059069
Cox7c	ENSMUSG00000017778	-0.521871179	0.002163447	0.087059069
Arl6ip1	ENSMUSG00000030654	-0.225904796	0.002173937	0.087059069
Pakap+Akap2+Palm2	ENSMUSG00000089945+ENSMUSG00000090053+E	0.307177976	0.002185768	0.087059069
Polr2l	ENSMUSG00000038489	-0.342408721	0.002193675	0.087059069
Crls1	ENSMUSG00000027357	-0.364633627	0.002201586	0.087059069
Ndufb5	ENSMUSG00000027673	-0.403985891	0.002203413	0.087059069
Hspa13	ENSMUSG00000032932	-0.3137879	0.002206189	0.087059069
Psmb1	ENSMUSG00000014769	-0.401958131	0.002217452	0.087059069
Snapc5	ENSMUSG00000032398	-0.506020191	0.002228652	0.087059069
Aimp1	ENSMUSG00000028029	-0.357651531	0.002257453	0.087059069
A430035B10Rik	ENSMUSG00000087305	-0.502494563	0.002274628	0.087059069
Ccdc85b	ENSMUSG00000095098	-0.326958988	0.002281658	0.087059069
Cox6a1	ENSMUSG00000041697	-0.327495368	0.002290441	0.087059069

Chchd3	ENSMUSG00000053768	-0.44316207	0.002298794	0.087059069
Cox5b	ENSMUSG00000061518	-0.282784433	0.002306281	0.087059069
Dhrs7b	ENSMUSG00000042569	-0.32540904	0.002318413	0.087059069
Pts	ENSMUSG00000032067	-0.485969273	0.002318613	0.087059069
Hist1h2bc	ENSMUSG00000018102	-0.496349664	0.002325155	0.087059069
Sepp1	ENSMUSG00000064373	-0.386031805	0.002325903	0.087059069
Lsm7	ENSMUSG00000035215	-0.477699102	0.002326005	0.087059069
Selk	ENSMUSG00000042682	-0.450319147	0.002335071	0.087059069
Ccdc153	ENSMUSG00000070306	-0.539249	0.002346539	0.087059069
Atp5h	ENSMUSG00000034566	-0.446351354	0.002350804	0.087059069
Spock3	ENSMUSG00000054162	-0.437908216	0.002356324	0.087059069
Rab28	ENSMUSG00000029128	-0.412194881	0.002361026	0.087059069
Acyp2	ENSMUSG00000060923	-0.573301435	0.002361342	0.087059069
Nenf	ENSMUSG00000037499	-0.437361588	0.00236485	0.087059069
2310009B15Rik	ENSMUSG00000079283	-0.454831474	0.002369441	0.087059069
Col1a1	ENSMUSG00000001506	0.949749936	0.002385363	0.087059069
Lims1	ENSMUSG00000019920	-0.268961726	0.002386745	0.087059069
Svip	ENSMUSG00000074093	-0.501889803	0.002388232	0.087059069
Ppargc1b	ENSMUSG00000033871	0.757455647	0.002396437	0.087059069
Mir682+Gm4149	ENSMUSG00000074800+ENSMUSG00000076236	-0.655604342	0.002398845	0.087059069
Gm44250	ENSMUSG00000107881	-0.375447196	0.002399757	0.087059069
Mfsd8	ENSMUSG00000025759	-0.294138808	0.002399873	0.087059069
Ndufa4	ENSMUSG00000029632	-0.531359016	0.00241074	0.087059069
mt-Rnr2	ENSMUSG00000064339	-0.517787714	0.002417334	0.087059069
9330132A10Rik	ENSMUSG00000043498	-0.570951302	0.002426058	0.087059069
Ormdl2	ENSMUSG00000025353	-0.452394539	0.002427952	0.087059069
Rap1b	ENSMUSG00000052681	-0.327497292	0.002436895	0.087059069
Glg1	ENSMUSG00000003316	0.501619146	0.002449437	0.087059069
0610009B22Rik	ENSMUSG00000007777	-0.550638084	0.002452604	0.087059069
Haus1	ENSMUSG00000041840	-0.334583063	0.002452892	0.087059069
Prdx1	ENSMUSG00000028691	-0.535520426	0.002463059	0.087059069
Bbs9	ENSMUSG00000035919	0.341246949	0.00246389	0.087059069
Rps27	ENSMUSG00000090733	-0.555397299	0.002465521	0.087059069
mt-Ti+mt-Nd1	ENSMUSG00000064342+ENSMUSG00000064341	-0.436674181	0.002469384	0.087059069
Scp2	ENSMUSG00000028603	-0.355415435	0.002479373	0.087059069
Alg14	ENSMUSG00000039887	-0.313972691	0.002484719	0.087059069
Fam174a	ENSMUSG00000051185	-0.44985293	0.002486991	0.087059069
Mphosph10	ENSMUSG00000030521	-0.419052999	0.002492725	0.087059069
Cmtm5	ENSMUSG00000040759	-0.214191416	0.002496409	0.087059069
Cnep1r1	ENSMUSG00000036810	-0.37241534	0.002502093	0.087059069
Capza2	ENSMUSG00000015733	-0.461622315	0.002515274	0.087059069
Pdzd2	ENSMUSG00000022197	0.60674184	0.002529049	0.087059069
Rpf2	ENSMUSG00000038510	-0.438774338	0.002533482	0.087059069
1810037I17Rik	ENSMUSG00000054091	-0.481978976	0.002535356	0.087059069
Zfp24	ENSMUSG00000051469	-0.330367481	0.002556707	0.087059069
Arl6	ENSMUSG00000022722	-0.468494788	0.002558404	0.087059069
Bud31	ENSMUSG00000038722	-0.441764634	0.002558801	0.087059069
Cacybp	ENSMUSG00000014226	-0.506046276	0.002561649	0.087059069
Scube1	ENSMUSG00000016763	0.611446777	0.002571821	0.087059069
Suv39h2	ENSMUSG00000026646	-0.361505214	0.002578193	0.087059069
Pex2	ENSMUSG00000040374	-0.494261038	0.002586337	0.087059069
Ndufa2	ENSMUSG00000014294	-0.565083835	0.002595015	0.087059069
Smim15	ENSMUSG00000071180	-0.562234455	0.002603617	0.087059069
Rpl31	ENSMUSG00000073702	-0.573347097	0.002614826	0.087059069
Hibch	ENSMUSG00000041426	-0.351213044	0.002617159	0.087059069
Psma6	ENSMUSG00000021024	-0.400267868	0.002618988	0.087059069
Them4	ENSMUSG00000028145	-0.409298462	0.002619106	0.087059069
Celrr	ENSMUSG00000097881	-0.455819933	0.002620241	0.087059069
Rgs10	ENSMUSG00000030844	-0.333016942	0.00262093	0.087059069

Sys1+Gm20458+Dbndd2	ENSMUSG00000017734+ENSMUSG00000090996+E	-0.350331912	0.002622915	0.087059069
Ubap2l	ENSMUSG00000042520	0.368163631	0.002624862	0.087059069
Hat1	ENSMUSG00000027018	-0.334135494	0.002626913	0.087059069
Serf1	ENSMUSG00000021643	-0.559407418	0.002642142	0.087059069
Cln6	ENSMUSG00000032245	0.355045899	0.002643489	0.087059069
Nudt7	ENSMUSG00000031767	-0.347586309	0.00265838	0.087059069
Mcee	ENSMUSG00000033429	-0.514711709	0.00266202	0.087059069
Rgcc	ENSMUSG00000022018	-0.435244338	0.002672081	0.087059069
Gnpda2	ENSMUSG00000029209	-0.530391665	0.002677223	0.087059069
2810402E24Rik	ENSMUSG00000099966	-0.464471244	0.002685367	0.087059069
Atp5j2	ENSMUSG00000038690	-0.615990024	0.002685724	0.087059069
Psma5	ENSMUSG00000068749	-0.280198859	0.002686357	0.087059069
Fdx1l	ENSMUSG00000079677	-0.470435003	0.002686902	0.087059069
Gm43511+Mrps18c	ENSMUSG00000104761+ENSMUSG00000016833	-0.643582774	0.002693301	0.087059069
Zfp367	ENSMUSG00000044934	-0.392121065	0.00270071	0.087059069
Il6st	ENSMUSG00000021756	0.284219753	0.002702255	0.087059069
Dio2	ENSMUSG00000007682	-0.464580655	0.002709091	0.087059069
Cox6c	ENSMUSG00000014313	-0.554092475	0.002714015	0.087059069
Tmed5	ENSMUSG00000063406	-0.604307811	0.002715397	0.087059069
Zfp52	ENSMUSG00000051341	-0.411174141	0.002716795	0.087059069
Ndufa7	ENSMUSG00000041881	-0.391084276	0.002732227	0.087059069
Sik1	ENSMUSG00000024042	0.549324497	0.002735957	0.087059069
Smim1	ENSMUSG00000078350	-0.313034718	0.002763576	0.087059069
Polr2k	ENSMUSG00000045996	-0.666619959	0.002774617	0.087059069
Cox6b1	ENSMUSG00000036751	-0.413729881	0.00277762	0.087059069
Exoc3l4	ENSMUSG00000021280	0.460295495	0.002789745	0.087059069
Tenm3	ENSMUSG00000031561	0.581672067	0.002791505	0.087059069
Serpinb1b	ENSMUSG00000051029	-0.45087612	0.002801359	0.087059069
Tmem219	ENSMUSG00000060538	-0.248286384	0.00280271	0.087059069
Kirrel3	ENSMUSG00000032036	0.335723266	0.002804488	0.087059069
Tmem229a	ENSMUSG00000048022	-0.387762496	0.002808488	0.087059069
1110008P14Rik	ENSMUSG00000039195	-0.320016163	0.002816537	0.087059069
Slirp	ENSMUSG00000021040	-0.582437716	0.002820554	0.087059069
Zfp273	ENSMUSG00000030446	-0.682021891	0.002836707	0.087059069
Aldh18a1	ENSMUSG00000025007	0.405758385	0.002842282	0.087059069
Uqcrq	ENSMUSG00000044894	-0.414690419	0.002845126	0.087059069
Hddc2	ENSMUSG00000000295	-0.507650428	0.00285291	0.087059069
Gm44958+Magohb	ENSMUSG00000030188+ENSMUSG00000107658	-0.614900966	0.002860587	0.087059069
Pde4d	ENSMUSG00000021699	0.27465523	0.002870918	0.087059069
Pigx	ENSMUSG00000023791	-0.402832577	0.002876429	0.087059069
Psma3	ENSMUSG00000060073	-0.507924068	0.002876562	0.087059069
Tma7	ENSMUSG00000091537	-0.483440272	0.002883265	0.087059069
Hspe1	ENSMUSG00000073676	-0.458795883	0.002885453	0.087059069
Resp18	ENSMUSG00000033061	-0.269168298	0.002902017	0.087059069
Paip2	ENSMUSG00000037058	-0.39132026	0.00291669	0.087059069
Cep63	ENSMUSG00000032534	-0.256104182	0.002923016	0.087059069
Mrpl22	ENSMUSG00000020514	-0.335903355	0.00293128	0.087059069
Tnfrsf1a	ENSMUSG00000030341	0.323270635	0.002931408	0.087059069
Cbr4	ENSMUSG00000031641	-0.45483905	0.002940427	0.087059069
Bend4	ENSMUSG00000092060	0.525100298	0.002940828	0.087059069
Prorsd1	ENSMUSG00000032673	-0.550900042	0.002948108	0.087059069
Snord42b+Rpl23a	ENSMUSG00000058546+ENSMUSG00000065676	-0.524828023	0.002948263	0.087059069
Smtn	ENSMUSG00000020439	0.579354367	0.002948753	0.087059069
Sub1	ENSMUSG00000022205	-0.472708384	0.002951414	0.087059069
Sep-15	ENSMUSG00000037072	-0.510769971	0.002953145	0.087059069
Ncoa1	ENSMUSG00000020647	0.354736552	0.00296657	0.087059069
Trhr	ENSMUSG00000038760	-0.370133716	0.002966722	0.087059069
Doc2b	ENSMUSG00000020848	0.369502814	0.002969983	0.087059069
Polb	ENSMUSG00000031536	-0.434782288	0.002975329	0.087059069

Guk1	ENSMUSG00000020444	-0.360233004	0.002977425	0.087059069
Psmd6	ENSMUSG00000021737	-0.270469249	0.00297904	0.087059069
Ndufc1	ENSMUSG00000037152	-0.539102626	0.002989122	0.087059069
Eif1a	ENSMUSG00000057561	-0.362180889	0.002990254	0.087059069
Mpc1-ps	ENSMUSG00000091498	-0.55778785	0.002996309	0.087059069
Zfp788	ENSMUSG00000074165	-0.322948643	0.003006398	0.087059069
Gm27734+Ntan1	ENSMUSG00000022681+ENSMUSG00000099314	-0.461098145	0.003009739	0.087059069
C1d	ENSMUSG00000000581	-0.591197662	0.003011365	0.087059069
Tyrobp	ENSMUSG00000030579	-0.533112966	0.003021465	0.087059069
Myo10	ENSMUSG00000022272	0.591998207	0.003028471	0.087059069
Dnajc24	ENSMUSG00000027166	-0.505875672	0.003042932	0.087059069
Calm2	ENSMUSG00000036438	-0.439849349	0.003044871	0.087059069
Slmo2	ENSMUSG00000016257	-0.321851991	0.003057321	0.087059069
Rps19	ENSMUSG00000040952	-0.47050524	0.003058272	0.087059069
Ndufb3	ENSMUSG00000026032	-0.47408692	0.003062041	0.087059069
Scoc	ENSMUSG00000063253	-0.355552028	0.003068898	0.087059069
Cript	ENSMUSG00000024146	-0.461290312	0.003082054	0.087059069
Rab29	ENSMUSG00000026433	-0.437325861	0.003084372	0.087059069
Ube2e1	ENSMUSG00000021774	-0.437721911	0.003085294	0.087059069
Atp5e	ENSMUSG00000016252	-0.526797678	0.003090102	0.087059069
Mrpl42	ENSMUSG00000062981	-0.492045309	0.003113822	0.087059069
Acbd5	ENSMUSG00000026781	-0.342407388	0.003119675	0.087059069
Fam96a	ENSMUSG00000032381	-0.386036882	0.003127656	0.087059069
Rps3a1	ENSMUSG00000028081	-0.498212921	0.003142964	0.087059069
Rabac1	ENSMUSG00000003380	-0.303773883	0.003143936	0.087059069
Lamtor5	ENSMUSG00000087260	-0.4705693	0.003145364	0.087059069
Syde1	ENSMUSG00000032714	0.257448315	0.003148306	0.087059069
Tstd3	ENSMUSG00000028251	-0.457027153	0.003156218	0.087059069
Bloc1s1+Rdh5	ENSMUSG00000090247+ENSMUSG00000025350	-0.488310218	0.003165745	0.087059069
Fabp3	ENSMUSG00000028773	-0.353828601	0.003186981	0.087059069
Ift74	ENSMUSG00000028576	-0.368443216	0.003200513	0.087059069
Mir1892+Sptssa	ENSMUSG00000106344+ENSMUSG00000044408	-0.564879018	0.003205111	0.087059069
Ndufv3	ENSMUSG00000024038	-0.297774986	0.003217475	0.087059069
BC002059	ENSMUSG00000060149	-0.434040619	0.003229689	0.087059069
Arsk	ENSMUSG00000021592	-0.294693697	0.003237229	0.087059069
Ankrd49	ENSMUSG00000031931	-0.479080905	0.00324937	0.087059069
Nme1+Gm20390+Nme2	ENSMUSG00000091228+ENSMUSG00000020857+E	-0.390532276	0.003251553	0.087059069
1810022K09Rik	ENSMUSG00000078784	-0.469721126	0.003253302	0.087059069
Gjb6	ENSMUSG00000040055	-0.242459963	0.003256525	0.087059069
Nts	ENSMUSG00000019890	-0.505786979	0.003268137	0.087059069
Atp5l	ENSMUSG00000038717	-0.538672595	0.003269096	0.087059069
Pole	ENSMUSG00000007080	0.543315386	0.003272498	0.087059069
Cenpo	ENSMUSG00000020652	0.444079417	0.003300783	0.087059069
Snrpd2	ENSMUSG00000040824	-0.434160399	0.003304143	0.087059069
Iqgap1	ENSMUSG00000030536	0.42521842	0.003306166	0.087059069
Sh3pxd2a	ENSMUSG00000053617	0.453951448	0.003310447	0.087059069
Dusp12	ENSMUSG00000026659	-0.2596112	0.00331748	0.087059069
Ankra2	ENSMUSG00000021661	-0.340334032	0.003317719	0.087059069
Cdh5	ENSMUSG00000031871	0.64122343	0.00331845	0.087059069
Ppia	ENSMUSG00000071866	-0.40415745	0.003324675	0.087059069
S100b	ENSMUSG00000033208	-0.386822804	0.003335139	0.087059069
mt-Co1	ENSMUSG00000064351	-0.378394151	0.003359724	0.087059069
Ndufa6	ENSMUSG00000022450	-0.502142731	0.003365363	0.087059069
Snord21+Rpl5	ENSMUSG00000064453+ENSMUSG00000058558	-0.421486562	0.003374398	0.087059069
Rps5	ENSMUSG00000012848	-0.541484138	0.00337876	0.087059069
Lama4	ENSMUSG00000019846	0.55184051	0.003399142	0.087059069
Ndufab1	ENSMUSG00000030869	-0.211698284	0.003399674	0.087059069
Smarcad1	ENSMUSG00000029920	-0.312094763	0.003404378	0.087059069
Uba3	ENSMUSG00000030061	-0.383595513	0.003406026	0.087059069

C330018D20Rik	ENSMUSG00000024592	-0.507901575	0.00342423	0.087059069
Ndufa12	ENSMUSG00000020022	-0.448405724	0.003426647	0.087059069
Snrpb2	ENSMUSG00000008333	-0.494297356	0.00343616	0.087059069
mt-Nd4+mt-Nd4l	ENSMUSG00000065947+ENSMUSG00000064363	-0.387704351	0.003451799	0.087059069
Ttc32	ENSMUSG00000066637	-0.627754879	0.003454515	0.087059069
Uqcrh	ENSMUSG00000063882	-0.508178237	0.003456999	0.087059069
Srp9	ENSMUSG00000026511	-0.506160225	0.003459253	0.087059069
1700066M21Rik	ENSMUSG00000038323	-0.445971059	0.003461266	0.087059069
Vps35	ENSMUSG00000031696	-0.368133835	0.003462383	0.087059069
Scamp2	ENSMUSG00000040188	0.266086418	0.003468344	0.087059069
Tspan2	ENSMUSG00000027858	-0.263695519	0.003475614	0.087059069
Lyrm5	ENSMUSG00000040370	-0.466586604	0.003480293	0.087059069
Ndufs8	ENSMUSG00000059734	-0.338404424	0.003480572	0.087059069
Cystm1	ENSMUSG00000046727	-0.299588006	0.003482883	0.087059069
Znrd1as	ENSMUSG00000036214	-0.479712902	0.003485613	0.087059069
Ddit3	ENSMUSG00000025408	-0.300013532	0.003485844	0.087059069
BC031181	ENSMUSG00000036299	-0.268485664	0.003487012	0.087059069
Slc38a3	ENSMUSG00000010064	0.367336436	0.003491597	0.087059069
Clec5a	ENSMUSG00000029915	-0.645329048	0.003514798	0.087059695
Sdk1	ENSMUSG00000039683	0.748926211	0.003540506	0.087059695
Emc2	ENSMUSG00000022337	-0.473466722	0.003541875	0.087059695
2410015M20Rik	ENSMUSG00000049760	-0.544233635	0.003545668	0.087059695
Edil3	ENSMUSG00000034488	-0.468204515	0.003553727	0.087059695
Smim4	ENSMUSG00000058351	-0.495634501	0.00356159	0.087059695
Amotl1	ENSMUSG00000013076	0.428276131	0.00357081	0.087059695
Rps14	ENSMUSG00000024608	-0.554071143	0.003575435	0.087059695
Nbas	ENSMUSG00000020576	0.402041026	0.003585033	0.087059695
Lsr	ENSMUSG00000001247	0.460702905	0.003585505	0.087059695
Bbip1	ENSMUSG00000084957	-0.540078539	0.003600561	0.087059695
Actr6	ENSMUSG00000019948	-0.578274583	0.003603373	0.087059695
Bcl2a1b+Mthfs+Gm29094	ENSMUSG00000100838+ENSMUSG00000066442+E	-0.497550652	0.00360361	0.087059695
Ccdc122	ENSMUSG00000034795	-0.635747312	0.003603716	0.087059695
Ppp1r14a	ENSMUSG00000037166	-0.326823514	0.003631539	0.087059695
Zscan21	ENSMUSG00000037017	0.252877034	0.003641988	0.087059695
Srp14	ENSMUSG00000009549	-0.429815869	0.003644043	0.087059695
Adcy9	ENSMUSG00000005580	0.557897207	0.003646799	0.087059695
Psmc6	ENSMUSG00000021832	-0.496295373	0.003660738	0.087059695
Atp5j	ENSMUSG00000022890	-0.457678537	0.003661004	0.087059695
Mob4	ENSMUSG00000025979	-0.478344146	0.003661369	0.087059695
Fam134b	ENSMUSG00000022270	-0.286051277	0.003671887	0.087059695
Nek7	ENSMUSG00000026393	-0.450092992	0.00367226	0.087059695
Fstl4	ENSMUSG00000036264	0.640223285	0.003673394	0.087059695
Mff	ENSMUSG00000026150	-0.306993646	0.003674426	0.087059695
Mir207+Dnaja1	ENSMUSG00000065452+ENSMUSG00000028410	-0.259958014	0.003679188	0.087059695
Dld	ENSMUSG00000020664	-0.319138404	0.003695232	0.08726821
Naa38	ENSMUSG00000059278	-0.509226838	0.003706361	0.087360086
Nae1	ENSMUSG00000031878	-0.329306551	0.003735436	0.087615839
Deb1	ENSMUSG00000032526	-0.475305696	0.003737765	0.087615839
Naca	ENSMUSG00000061315	-0.410749815	0.003740473	0.087615839
Fastkd2	ENSMUSG00000025962	-0.2867882	0.003748931	0.087615839
Lpar6	ENSMUSG00000033446	-0.411746525	0.003773586	0.087615839
Nmd3	ENSMUSG00000027787	-0.355447802	0.003778427	0.087615839
Pcp4+Igsf5	ENSMUSG00000090223+ENSMUSG00000000159	-0.406239461	0.003780063	0.087615839
Zfp950	ENSMUSG00000074733	-0.392036835	0.003783964	0.087615839
Rpl7	ENSMUSG00000043716	-0.44956007	0.003801171	0.087615839
Smad3	ENSMUSG00000032402	0.460533809	0.003802024	0.087615839
Txn1	ENSMUSG00000028367	-0.344074983	0.003807893	0.087615839
Ube2b	ENSMUSG00000020390	-0.38815297	0.003813685	0.087615839
Rpsa	ENSMUSG00000032518	-0.411342484	0.003829094	0.087615839

Smim8	ENSMUSG00000028295	-0.482671811	0.003829514	0.087615839
Pcnp	ENSMUSG00000071533	-0.370395267	0.003857465	0.087615839
Rap1a	ENSMUSG00000068798	-0.505797541	0.003862788	0.087615839
Mir7014+Ecm1	ENSMUSG00000028108+ENSMUSG00000098737	0.47564208	0.00388787	0.087615839
Thsd4	ENSMUSG00000032289	0.627684972	0.00389167	0.087615839
Zfp503	ENSMUSG00000039081	0.487277493	0.003894219	0.087615839
Lrfn1	ENSMUSG00000030600	0.381050823	0.003894389	0.087615839
Tuft1	ENSMUSG00000005968	0.339836606	0.003909021	0.087615839
Pole4	ENSMUSG00000030042	-0.42995419	0.003910139	0.087615839
Tmem126b	ENSMUSG00000030614	-0.425401577	0.003917284	0.087615839
Mphosph8	ENSMUSG00000079184	-0.18676145	0.003918496	0.087615839
Polr2g	ENSMUSG00000071662	-0.258644397	0.003932104	0.087615839
Cyp2j12	ENSMUSG00000081225	-0.446936634	0.003945718	0.087615839
Aph1b	ENSMUSG00000032375	-0.245122067	0.003953683	0.087615839
Pdcd10	ENSMUSG00000027835	-0.517438695	0.003954772	0.087615839
Tceb1	ENSMUSG00000079658	-0.502955181	0.003955799	0.087615839
Tomm5	ENSMUSG00000078713	-0.562334802	0.003961328	0.087615839
Itgb1bp1	ENSMUSG00000062352	-0.263794219	0.003961654	0.087615839
Greb1l	ENSMUSG00000042942	0.42114679	0.00396575	0.087615839
S100a13	ENSMUSG00000042312	-0.516830906	0.003967989	0.087615839
Fkbp3	ENSMUSG00000020949	-0.474232203	0.00397085	0.087615839
Adck2+Ndufb2+Gm42420	ENSMUSG00000107071+ENSMUSG00000002416+E	-0.414635054	0.003974644	0.087615839
Romo1	ENSMUSG00000067847	-0.497956723	0.003982241	0.087615839
Dnajc13	ENSMUSG00000032560	0.253746115	0.003985838	0.087615839
Cept1	ENSMUSG00000040774	-0.324099433	0.003998428	0.08770296
Adamts1	ENSMUSG00000022893	0.46172751	0.004004336	0.08770296
mt-Cytb	ENSMUSG00000064370	-0.378398197	0.004013294	0.087739912
Gm14325	ENSMUSG00000095362	-0.598698711	0.004025205	0.087806812
Snx10	ENSMUSG00000038301	-0.307278893	0.004045547	0.087806812
2610316D01Rik	ENSMUSG00000097040	-0.39861096	0.004049525	0.087806812
Glrx3	ENSMUSG00000031068	-0.403601717	0.004061255	0.087806812
Psenen	ENSMUSG00000036835	-0.301629312	0.004063248	0.087806812
Sumo1	ENSMUSG00000026021	-0.536482144	0.004066878	0.087806812
Mrps21	ENSMUSG00000054312	-0.380516028	0.004074446	0.087806812
Heatr5b	ENSMUSG00000039414	0.451961541	0.004083093	0.087806812
Commd8	ENSMUSG00000029213	-0.396952937	0.004105936	0.087806812
A830082K12Rik	ENSMUSG00000087143	-0.366104526	0.004115596	0.087806812
Wwc2	ENSMUSG00000031563	0.211300926	0.004121222	0.087806812
Gm15421+Orc5	ENSMUSG00000029012+ENSMUSG00000084235	-0.255473995	0.004131791	0.087806812
Ccdc53	ENSMUSG00000020056	-0.429415238	0.004138654	0.087806812
Plcb3	ENSMUSG00000024960	0.603530856	0.004140243	0.087806812
2310009A05Rik	ENSMUSG00000098332	-0.546707687	0.004142145	0.087806812
Zfp131	ENSMUSG00000094870	-0.391652687	0.004144338	0.087806812
Etfa	ENSMUSG00000032314	-0.242108589	0.004148456	0.087806812
Qdpr	ENSMUSG00000015806	-0.294853187	0.004151319	0.087806812
Lamb1	ENSMUSG00000002900	0.652129018	0.004156998	0.087806812
Cetn4	ENSMUSG00000045031	-0.442914113	0.00416212	0.087806812
Nmu	ENSMUSG00000029236	-0.548321929	0.00416915	0.087806812
Kbtbd7	ENSMUSG00000043881	-0.375659633	0.004181993	0.087923849
Gm37988+Lypla1+Tcea1	ENSMUSG00000033813+ENSMUSG00000025903+E	-0.460382458	0.004193435	0.088011093
E2f5	ENSMUSG00000027552	-0.44301304	0.004215971	0.088277384
Pigp+Gm15310	ENSMUSG00000022940+ENSMUSG00000089814	-0.494836083	0.004221285	0.088277384
Zfand6	ENSMUSG00000030629	-0.420172926	0.004238589	0.088277384
Znhit3	ENSMUSG00000020526	-0.514152174	0.004240685	0.088277384
Rps18	ENSMUSG00000008668	-0.53478243	0.004242848	0.088277384
Myo1c	ENSMUSG00000017774	0.381236433	0.00425334	0.088277384
Smim10l1+RP24-470M22.1	ENSMUSG00000072704+ENSMUSG00000107838	-0.365093778	0.004257328	0.088277384
Haus2	ENSMUSG00000027285	-0.399440197	0.004284541	0.088521346
Arntl	ENSMUSG00000055116	0.350758392	0.004300402	0.088521346

Fabp7	ENSMUSG00000019874	-0.648645055	0.004309314	0.088521346
Gnai3	ENSMUSG00000000001	-0.231289625	0.004314699	0.088521346
Rpl41	ENSMUSG00000093674	-0.525968151	0.004340377	0.088521346
Cstb	ENSMUSG00000005054	-0.414645032	0.004343185	0.088521346
Ndufb4	ENSMUSG00000022820	-0.449440631	0.004344937	0.088521346
Zfp455	ENSMUSG00000051037	-0.571914224	0.004345845	0.088521346
Eif4a3	ENSMUSG00000025580	0.344897409	0.004351294	0.088521346
Ubqln4	ENSMUSG00000008604	0.165738985	0.00435881	0.088521346
Ankrd34a	ENSMUSG00000049097	0.36147313	0.004368915	0.088521346
Mrpl47	ENSMUSG00000037531	-0.455779404	0.004378514	0.088521346
Tmed2	ENSMUSG00000029390	-0.284140068	0.004385769	0.088521346
Nmi	ENSMUSG00000026946	-0.445260067	0.004388063	0.088521346
Vdac3	ENSMUSG00000008892	-0.296010464	0.004390385	0.088521346
Sfrp1	ENSMUSG00000031548	0.310216068	0.00439142	0.088521346
Aif1	ENSMUSG00000024397	-0.542345612	0.004393792	0.088521346
Eva1a	ENSMUSG00000035104	-0.43084619	0.004401779	0.08853444
Eif5a2	ENSMUSG00000050192	-0.428795866	0.004411123	0.08855194
Rpl18a	ENSMUSG00000045128	-0.492993031	0.004424995	0.08855194
1110008F13Rik	ENSMUSG00000027637	-0.293976544	0.004430672	0.08855194
Sar1b	ENSMUSG00000020386	-0.511931403	0.004473715	0.08855194
Frem1	ENSMUSG00000059049	0.295069406	0.004482621	0.08855194
Metrn	ENSMUSG00000002274	-0.518204983	0.004483804	0.08855194
1110065P20Rik	ENSMUSG00000078570	-0.440790435	0.004498689	0.08855194
Thnsl1	ENSMUSG00000048550	-0.296013026	0.004499055	0.08855194
Midn	ENSMUSG00000035621	0.492475809	0.004511824	0.08855194
Mettl20	ENSMUSG00000039958	-0.409125975	0.004522133	0.08855194
Lamc3	ENSMUSG00000026840	0.634698605	0.004522187	0.08855194
Gas7	ENSMUSG00000033066	0.304821342	0.004523122	0.08855194
Uqcrb	ENSMUSG00000021520	-0.494475831	0.004524065	0.08855194
Pno1	ENSMUSG00000020116	-0.413432281	0.004526015	0.08855194
Mrpl54	ENSMUSG00000034932	-0.432120382	0.004526018	0.08855194
Tmsb15b2+Tmsb15l+Tmsb1	ENSMUSG00000072955+ENSMUSG00000089768+E	-0.590917886	0.004527063	0.08855194
Gm25091+Gm23202+Rpl21	ENSMUSG00000077185+ENSMUSG00000077564+E	-0.497542936	0.004549857	0.08855194
Ccdc90b	ENSMUSG00000030613	-0.557205862	0.00455078	0.08855194
Zfp933	ENSMUSG00000059423	-0.384132915	0.00456021	0.08855194
Ptbp1	ENSMUSG00000006498	0.29779899	0.004560248	0.08855194
Tmem126a	ENSMUSG00000030615	-0.463050816	0.004560413	0.08855194
Eif3i	ENSMUSG00000028798	-0.251311923	0.004569867	0.08855194
Srsf7	ENSMUSG00000024097	-0.273805509	0.004574292	0.08855194
Gm32444	ENSMUSG00000102729	-0.375740184	0.004605889	0.08855194
Gm24016+Rps20	ENSMUSG00000088351+ENSMUSG00000028234	-0.521211498	0.004618579	0.08855194
Lmo1	ENSMUSG00000036111	-0.411691343	0.004620156	0.08855194
Calcrl	ENSMUSG00000059588	-0.502392538	0.004634904	0.08855194
Pbxip1	ENSMUSG00000042613	0.23799186	0.004640277	0.08855194
1110034G24Rik	ENSMUSG00000044991	-0.33739133	0.004641141	0.08855194
Gm27943+Glb1l	ENSMUSG00000026200+ENSMUSG00000099204	0.220457577	0.004658	0.08855194
Tmem167	ENSMUSG00000012422	-0.505804691	0.004662552	0.08855194
Ptpre	ENSMUSG00000041836	0.287521057	0.004668597	0.08855194
Rps28	ENSMUSG00000067288	-0.362980213	0.004668683	0.08855194
Rpl37rt	ENSMUSG00000072692	-0.655855524	0.004670943	0.08855194
Cd53	ENSMUSG00000040747	-0.645897288	0.004676029	0.08855194
Rapgef2	ENSMUSG00000062232	0.393007955	0.004678168	0.08855194
Rpa3	ENSMUSG00000012483	-0.500638419	0.004684554	0.08855194
Nudt4	ENSMUSG00000020029	-0.229986523	0.004689094	0.08855194
Lyrm4	ENSMUSG00000046573	-0.408743068	0.004703265	0.08855194
Sf3b6	ENSMUSG00000037361	-0.558300589	0.004705952	0.08855194
Sep-07	ENSMUSG00000001833	-0.426942306	0.004717678	0.08855194
Fbxo10	ENSMUSG00000048232	0.384072181	0.004721741	0.08855194
Nudt5	ENSMUSG00000025817	-0.323824841	0.004723138	0.08855194

Psma2	ENSMUSG00000015671	-0.455371417	0.00472551	0.08855194
Kcnj6	ENSMUSG00000043301	0.596870895	0.004746723	0.088806199
Cetn3	ENSMUSG00000021537	-0.54456163	0.004767562	0.088806199
Pdgfrb	ENSMUSG00000024620	0.562836271	0.004776095	0.088806199
Rps23	ENSMUSG00000049517	-0.506417257	0.004781465	0.088806199
H2afz	ENSMUSG00000037894	-0.439942624	0.004785615	0.088806199
Stx8	ENSMUSG00000020903	-0.272836127	0.004793827	0.088806199
Ube2v2	ENSMUSG00000022674	-0.545697158	0.004801541	0.088806199
Cops2	ENSMUSG00000027206	-0.543964734	0.004804052	0.088806199
Tceb2	ENSMUSG00000055839	-0.394865423	0.004805606	0.088806199
Rps21	ENSMUSG00000039001	-0.532034496	0.004817141	0.088806199
Spats2l	ENSMUSG00000038305	0.266237654	0.00483264	0.088806199
Mgst3	ENSMUSG00000026688	-0.302019015	0.004841041	0.088806199
2700094K13Rik	ENSMUSG00000076437	-0.393377181	0.004841908	0.088806199
Rpl37	ENSMUSG00000041841	-0.491646794	0.004848347	0.088806199
Rrp12	ENSMUSG00000035049	0.525894852	0.00484946	0.088806199
Otud6b	ENSMUSG00000040550	-0.396251488	0.004868884	0.089026804
Mocs2	ENSMUSG00000015536	-0.345125548	0.004883022	0.089041592
Pomp	ENSMUSG00000029649	-0.382693986	0.004884449	0.089041592
Nostrin	ENSMUSG00000034738	-0.50119104	0.004959771	0.090144607
Myh10	ENSMUSG00000020900	0.574144962	0.004959895	0.090144607
Nudcd1	ENSMUSG00000038736	-0.303169094	0.004980661	0.090243179
Ilf3	ENSMUSG00000032178	0.253461174	0.004985359	0.090243179
Klhl2	ENSMUSG00000031605	-0.204312711	0.004987753	0.090243179
Cnot1	ENSMUSG00000036550	0.341969954	0.005022429	0.090479281
Ociad2	ENSMUSG00000029153	-0.261890823	0.005026795	0.090479281
Cdc42bpg	ENSMUSG00000024769	0.448698992	0.005028581	0.090479281
Etv6	ENSMUSG00000030199	0.403536151	0.005030792	0.090479281
Nudt19	ENSMUSG00000034875	-0.402015463	0.005053424	0.090496185
Fat4	ENSMUSG00000046743	0.582958552	0.005057579	0.090496185
Zfp931	ENSMUSG00000078861	-0.629544009	0.005062796	0.090496185
Ccnc	ENSMUSG00000028252	-0.464725112	0.005068462	0.090496185
Zkscan17	ENSMUSG00000020472	0.333664389	0.005074453	0.090496185
Hltf	ENSMUSG00000002428	-0.245219366	0.005076725	0.090496185
4930522L14Rik	ENSMUSG00000072762	-0.628685923	0.005095164	0.090690908
Hdac2	ENSMUSG00000019777	-0.205026404	0.00510412	0.090716527
Aamdc	ENSMUSG00000035642	-0.497484702	0.00512081	0.090733492
Rarg	ENSMUSG00000001288	0.388484201	0.005124292	0.090733492
Btf3l4	ENSMUSG00000028568	-0.347600757	0.00512763	0.090733492
Oprd1	ENSMUSG00000050511	0.463036857	0.005146654	0.090911486
Fbxl3	ENSMUSG00000022124	-0.410472302	0.005152756	0.090911486
Tenm2	ENSMUSG00000049336	0.624189357	0.005164612	0.090952056
Lyrm2	ENSMUSG00000045854	-0.458982049	0.005178279	0.090952056
Hint1	ENSMUSG00000020267	-0.414517966	0.005183244	0.090952056
Tm2d1	ENSMUSG00000028563	-0.524647399	0.005192299	0.090952056
Ephb2	ENSMUSG00000028664	0.613180462	0.005202672	0.090952056
Nectin2	ENSMUSG00000062300	0.377193048	0.005207581	0.090952056
Srp19	ENSMUSG00000014504	-0.451555619	0.005221111	0.090952056
Itgb3bp	ENSMUSG00000028549	-0.555688072	0.005224579	0.090952056
Crbn	ENSMUSG00000005362	-0.410130423	0.005224655	0.090952056
Hexdc	ENSMUSG00000039307	0.238140778	0.005230422	0.090952056
Grpel1	ENSMUSG00000029198	-0.268368022	0.005261002	0.091323104
Mtrf1l	ENSMUSG00000019774	-0.293229132	0.0052751	0.091323104
Mttp	ENSMUSG00000028158	0.216227139	0.005299371	0.091323104
Thoc7	ENSMUSG00000053453	-0.410496185	0.005299483	0.091323104
Esd	ENSMUSG00000021996	-0.291056138	0.005300606	0.091323104
Dusp28	ENSMUSG00000047067	-0.411643284	0.005302903	0.091323104
Ptcd1	ENSMUSG00000029624	0.36916688	0.005304731	0.091323104
Vps26b	ENSMUSG00000031988	0.23403852	0.005329285	0.091615123

Myl6	ENSMUSG00000090841	-0.428833989	0.005343198	0.091692633
Taf1a	ENSMUSG00000072258	0.263834815	0.005353866	0.091692633
Fxr1	ENSMUSG00000027680	-0.265277586	0.005356734	0.091692633
Tmem69	ENSMUSG00000055900	-0.34219413	0.005364186	0.091692633
Ncan	ENSMUSG00000002341	0.513025131	0.005379474	0.091698198
Ptprb	ENSMUSG00000020154	0.403722981	0.005392191	0.091698198
Dmp1	ENSMUSG00000029307	0.511151147	0.005405656	0.091698198
Atp6v1g1	ENSMUSG00000039105	-0.288559395	0.005411925	0.091698198
Axin1	ENSMUSG00000024182	0.31631144	0.005427814	0.091698198
Rpl29	ENSMUSG00000048758	-0.356163193	0.00543629	0.091698198
Uggt1	ENSMUSG00000037470	0.366169157	0.005447863	0.091698198
Hnmt	ENSMUSG00000026986	-0.528988243	0.005456864	0.091698198
Slc12a2	ENSMUSG00000024597	-0.323884293	0.005458586	0.091698198
Gstm7	ENSMUSG00000004035	-0.270190635	0.005459689	0.091698198
Slc39a12	ENSMUSG00000036949	-0.219419671	0.005469144	0.091698198
Rab43+Isy1+Gm45140	ENSMUSG00000030056+ENSMUSG00000107928+E	0.173823553	0.0054791	0.091698198
Relt	ENSMUSG00000008318	0.219417199	0.005490988	0.091698198
4933434E20Rik	ENSMUSG00000027942	-0.366305289	0.005497777	0.091698198
Syn3	ENSMUSG00000059602	0.613330343	0.005505347	0.091698198
Vip	ENSMUSG00000019772	-0.367956291	0.005508519	0.091698198
Fchsd1	ENSMUSG00000038524	0.278850883	0.005523471	0.091698198
Ndufs6	ENSMUSG00000021606	-0.424073549	0.005523924	0.091698198
Eef1e1	ENSMUSG00000001707	-0.525245129	0.005528974	0.091698198
Esyt3	ENSMUSG00000037681	0.510493938	0.00553965	0.091698198
3110082I17Rik	ENSMUSG00000053553	-0.348313577	0.005542058	0.091698198
Csmd1	ENSMUSG00000060924	0.570657347	0.005548068	0.091698198
Gm43848	ENSMUSG00000106317	-0.430461709	0.005549934	0.091698198
Abhd17b	ENSMUSG00000047368	-0.324461145	0.005567421	0.091698198
Rpl11	ENSMUSG00000059291	-0.48804384	0.005575454	0.091698198
Fam19a1	ENSMUSG00000059187	-0.234582742	0.005595259	0.091698198
Pcdhb17	ENSMUSG00000046387	0.266152506	0.005598334	0.091698198
Agpat5	ENSMUSG00000031467	-0.240356391	0.005600602	0.091698198
Lamtor4	ENSMUSG00000050552	-0.477330235	0.005606887	0.091698198
Rps15	ENSMUSG00000063457	-0.422471774	0.005616172	0.091698198
Rida	ENSMUSG00000022323	-0.565433243	0.005617103	0.091698198
Gm12057+Gm28048+Gm12	ENSMUSG00000098650+ENSMUSG00000051355+E	-0.394789278	0.005617138	0.091698198
Gpm6a	ENSMUSG00000031517	-0.293500076	0.005628685	0.091698198
Cbwd1	ENSMUSG00000024878	-0.463765875	0.005639964	0.091698198
Slc8a2	ENSMUSG00000030376	0.517531522	0.005653665	0.091698198
Gm13341	ENSMUSG00000083863	-0.689974846	0.005654614	0.091698198
Crem	ENSMUSG00000063889	-0.479732644	0.005670553	0.091698198
Rheb	ENSMUSG00000028945	-0.330973378	0.005677208	0.091698198
Cldnd1	ENSMUSG00000022744	-0.284576966	0.005686374	0.091698198
Ly86	ENSMUSG00000021423	-0.51106286	0.005688938	0.091698198
Mrpl18	ENSMUSG00000057388	-0.405880981	0.005689982	0.091698198
Mzt1	ENSMUSG00000033186	-0.44622173	0.005694583	0.091698198
Bnip3l	ENSMUSG00000022051	-0.312123123	0.005697069	0.091698198
Bbox1	ENSMUSG00000041660	-0.378038216	0.005705509	0.091698198
Cd52	ENSMUSG00000000682	-0.571521941	0.005706442	0.091698198
Cbfa2t3	ENSMUSG00000006362	0.537775406	0.005721851	0.09181769
Rec8	ENSMUSG00000002324	0.469112401	0.005729095	0.09181769
Thada	ENSMUSG00000024251	0.350358174	0.005744093	0.091935963
Smyd3	ENSMUSG00000055067	0.192340943	0.005760772	0.091942352
Cnih4	ENSMUSG00000062169	-0.285437012	0.005766986	0.091942352
Aebp1	ENSMUSG00000020473	0.727522154	0.005769041	0.091942352
Pdhb	ENSMUSG00000021748	-0.237499698	0.005774967	0.091942352
Zfp677	ENSMUSG00000062743	-0.529780683	0.005798373	0.092193367
Snord15b+Rps3	ENSMUSG00000064966+ENSMUSG00000030744	-0.46985973	0.005808013	0.09222514
Mrpl24	ENSMUSG00000019710	-0.235874622	0.005836155	0.092429528

Ufsp2	ENSMUSG00000031634	-0.205874686	0.005837772	0.092429528
Zfp946	ENSMUSG00000071266	-0.480218248	0.005850653	0.092429528
Sec61b	ENSMUSG00000053317	-0.432181888	0.005854423	0.092429528
Akip1	ENSMUSG00000031023	-0.320854355	0.00585918	0.092429528
Tex10	ENSMUSG00000028345	-0.216954453	0.005881518	0.092499493
Tmem33	ENSMUSG00000037720	-0.318301203	0.005886179	0.092499493
E130311K13Rik	ENSMUSG00000048581	-0.416618616	0.005896488	0.092499493
Trmt10c	ENSMUSG00000044763	-0.431513154	0.00589871	0.092499493
Smc4	ENSMUSG00000034349	-0.349475411	0.005917329	0.092499493
Zfp518b	ENSMUSG00000046572	0.289514432	0.00592732	0.092499493
Snrpe	ENSMUSG00000090553	-0.558259673	0.005927327	0.092499493
Zfp938	ENSMUSG00000062931	-0.524362835	0.005931754	0.092499493
Rpp30	ENSMUSG00000024800	-0.431787812	0.005932599	0.092499493
Prkrir	ENSMUSG00000030753	-0.379819519	0.005954582	0.09272244
Fcho2	ENSMUSG00000041685	-0.455197299	0.005992946	0.092820295
Rplp1	ENSMUSG00000007892	-0.519257606	0.006001224	0.092820295
Cops3	ENSMUSG00000019373	-0.23147652	0.006001716	0.092820295
Sft2d1+T2	ENSMUSG00000058159+ENSMUSG00000073468	-0.216997823	0.006006504	0.092820295
Atf5	ENSMUSG00000038539	-0.295963456	0.006015063	0.092820295
Hcn4	ENSMUSG00000032338	0.647313916	0.006026643	0.092820295
Prdm11	ENSMUSG00000075028	0.514764926	0.006029065	0.092820295
Lmo7	ENSMUSG00000033060	0.283784826	0.006041231	0.092820295
Glis2	ENSMUSG00000014303	0.412978375	0.006049863	0.092820295
Mboat7	ENSMUSG00000035596	0.390458247	0.006053861	0.092820295
Smdt1+Fam109b	ENSMUSG00000049687+ENSMUSG00000022452	-0.460824933	0.006076204	0.092820295
Fam103a1	ENSMUSG00000038646	-0.497312837	0.006083012	0.092820295
Fbxo33	ENSMUSG00000035329	-0.316094539	0.006083047	0.092820295
Mkks	ENSMUSG00000027274	-0.460966267	0.006087248	0.092820295
Igf1r	ENSMUSG00000005533	0.626544643	0.006122977	0.092820295
Dpy30	ENSMUSG00000024067	-0.419339428	0.006131112	0.092820295
Gng11	ENSMUSG00000032766	-0.52016937	0.006131709	0.092820295
Uqcr11	ENSMUSG00000020163	-0.457028586	0.006138737	0.092820295
Rpl23	ENSMUSG00000071415	-0.453446153	0.006139228	0.092820295
Rcbtb2	ENSMUSG00000022106	-0.218235405	0.006142121	0.092820295
Atp6v1f	ENSMUSG00000004285	-0.340160814	0.006149141	0.092820295
Kdm3b	ENSMUSG00000038773	0.322326815	0.006162135	0.092820295
Atrn	ENSMUSG00000027312	0.390456639	0.006179342	0.092820295
Fbxo8	ENSMUSG00000038206	-0.319634677	0.006199567	0.092820295
Pcna	ENSMUSG00000027342	-0.32763098	0.006205655	0.092820295
Tmem128	ENSMUSG00000067365	-0.434288394	0.006207741	0.092820295
Phf10	ENSMUSG00000023883	-0.236880321	0.006218083	0.092820295
Gm6377+Sh3bgrl	ENSMUSG00000031246+ENSMUSG00000048621	-0.513285823	0.006226094	0.092820295
Uqcrfs1	ENSMUSG00000038462	-0.223181918	0.006227883	0.092820295
Gpr22	ENSMUSG00000044067	-0.540189988	0.006229722	0.092820295
Evi2a+Gm21975+Evi2b	ENSMUSG00000093938+ENSMUSG00000070354+E	-0.22578873	0.00623129	0.092820295
Fn1	ENSMUSG00000026193	1.177891357	0.006238507	0.092820295
Cdc26	ENSMUSG00000066149	-0.348156592	0.006246113	0.092820295
Lmtk2	ENSMUSG00000038970	0.566489916	0.00625009	0.092820295
Coq7	ENSMUSG00000030652	-0.277663863	0.006250605	0.092820295
Ccdc43	ENSMUSG00000020925	-0.2749832	0.006251044	0.092820295
Adh5	ENSMUSG00000028138	-0.365435099	0.006251699	0.092820295
Sptb	ENSMUSG00000021061	0.747331459	0.006253141	0.092820295
Ano6	ENSMUSG00000064210	0.291629062	0.006267819	0.092923889
Kcnb1	ENSMUSG00000050556	0.491759564	0.006277305	0.092950328
Wdr5	ENSMUSG00000026917	0.240885577	0.006301531	0.093082543
Ubl5	ENSMUSG00000084786	-0.410832666	0.006305308	0.093082543
Bcas1	ENSMUSG00000013523	-0.324316305	0.006315182	0.093082543
Dmtf1	ENSMUSG00000042508	-0.21939365	0.006317087	0.093082543
Pfdn5+Myg1	ENSMUSG00000001289+ENSMUSG00000001285	-0.455803476	0.006351725	0.093176191

Lgals1	ENSMUSG00000068220	-0.514129035	0.006361419	0.093176191
Rbm7	ENSMUSG00000042396	-0.481100415	0.006362756	0.093176191
Gm21987+Cox17+Popdc2	ENSMUSG00000095464+ENSMUSG00000046516+E	-0.453791352	0.006374945	0.093176191
Acot13	ENSMUSG00000006717	-0.481943666	0.006382991	0.093176191
Swt1	ENSMUSG00000052748	-0.372886022	0.006386988	0.093176191
Ost4	ENSMUSG00000038803	-0.533532414	0.006387906	0.093176191
Klf4	ENSMUSG00000003032	0.671835828	0.00638816	0.093176191
Igfbp5	ENSMUSG00000026185	0.414912708	0.006392931	0.093176191
Ehbp1	ENSMUSG00000042302	0.178862007	0.00641116	0.093329174
Gm28041+Zfp953+Gm2804	ENSMUSG00000098692+ENSMUSG00000078995+E	-0.463459928	0.006421536	0.093367584
Dag1	ENSMUSG00000039952	0.413848305	0.006435668	0.093440954
Eftud2	ENSMUSG00000020929	0.308237107	0.006442068	0.093440954
Scrg1	ENSMUSG00000031610	-0.547681169	0.006463028	0.093616503
Spcs1	ENSMUSG00000021917	-0.485944934	0.006473997	0.093616503
Selt	ENSMUSG00000075700	-0.345695754	0.006485167	0.093616503
Ublcp1	ENSMUSG00000041231	-0.254644923	0.006496654	0.093616503
Yaf2	ENSMUSG00000022634	-0.294898747	0.006510015	0.093616503
2500004C02Rik	ENSMUSG00000073236	-0.385938649	0.006511156	0.093616503
Nipa2	ENSMUSG00000030452	-0.274074901	0.006520186	0.093616503
Decr1	ENSMUSG00000028223	-0.355861809	0.006527568	0.093616503
Cycs	ENSMUSG00000063694	-0.467183745	0.00654995	0.093616503
Itpa	ENSMUSG00000074797	-0.372215714	0.006558159	0.093616503
Ndufa5	ENSMUSG00000023089	-0.511802903	0.006559337	0.093616503
Nt5c3	ENSMUSG00000029780	-0.45588795	0.006562854	0.093616503
Acat1	ENSMUSG00000032047	-0.222539737	0.006566409	0.093616503
Zfp811	ENSMUSG00000055202	-0.332538114	0.006588229	0.093616503
Rpl35	ENSMUSG00000062997	-0.485897796	0.006590291	0.093616503
Fam136a	ENSMUSG00000057497	-0.278684868	0.006592587	0.093616503
Rpl26	ENSMUSG00000060938	-0.528984097	0.006600518	0.093616503
Grin2b	ENSMUSG00000030209	0.619352983	0.006603826	0.093616503
Rps24	ENSMUSG00000025290	-0.49248234	0.006612737	0.093616503
Fabp5	ENSMUSG00000027533	-0.414691317	0.006630478	0.093616503
Erich3	ENSMUSG00000078161	0.370041191	0.006634976	0.093616503
Sarnp	ENSMUSG00000078427	-0.482171817	0.006638822	0.093616503
Paf1	ENSMUSG00000003437	0.323130243	0.006640123	0.093616503
Rps12	ENSMUSG00000061983	-0.495082854	0.006647003	0.093616503
4833423E24Rik	ENSMUSG00000075217	-0.39770999	0.006652481	0.093616503
Gm17018	ENSMUSG00000041035	-0.308355667	0.006659982	0.093616503
Slc23a2	ENSMUSG00000027340	0.332304047	0.006663621	0.093616503
Lyst	ENSMUSG00000019726	0.399667801	0.006688025	0.093796763
Adcy7	ENSMUSG00000031659	0.453648337	0.006716471	0.093796763
Sec22c	ENSMUSG00000061536	0.228006444	0.006716538	0.093796763
Itga1	ENSMUSG00000042284	0.355014535	0.00671722	0.093796763
Plxnc1	ENSMUSG00000074785	0.269872872	0.006728434	0.093796763
Pik3ap1	ENSMUSG00000025017	0.388677845	0.006730567	0.093796763
Rpl34	ENSMUSG00000062006	-0.51397758	0.006730858	0.093796763
Vangl2	ENSMUSG00000026556	0.404590095	0.006740609	0.0938243
Fis1	ENSMUSG00000019054	-0.444617417	0.006752782	0.093885447
Zfp426	ENSMUSG00000059475	-0.194366649	0.006774409	0.093932153
Mbnl2	ENSMUSG00000022139	-0.315815384	0.006779533	0.093932153
Nudcd2	ENSMUSG00000020328	-0.405567011	0.006781835	0.093932153
Nus1	ENSMUSG00000023068	-0.326686263	0.006799768	0.093932153
Ech1	ENSMUSG00000053898	-0.214339784	0.006801749	0.093932153
Eif3e	ENSMUSG00000022336	-0.555228961	0.006809361	0.093932153
Golga4	ENSMUSG00000038708	0.299150155	0.006811637	0.093932153
Supt6	ENSMUSG00000002052	0.459079094	0.006823283	0.093932153
Rpl38	ENSMUSG00000057322	-0.497099405	0.006832304	0.093932153
Trappc6a	ENSMUSG00000002043	-0.327120905	0.006848196	0.093932153
Irs1	ENSMUSG00000055980	0.285930696	0.006858446	0.093932153

Pttg1	ENSMUSG00000020415	-0.210261205	0.006859763	0.093932153
Xrcc4	ENSMUSG00000021615	-0.484011143	0.006869416	0.093932153
Eapp	ENSMUSG00000054302	-0.365541235	0.006874522	0.093932153
Rwdd1	ENSMUSG00000019782	-0.410035627	0.006874902	0.093932153
Plekhb1	ENSMUSG00000030701	-0.261812938	0.006880678	0.093932153
4930570G19Rik	ENSMUSG00000085084	-0.454259475	0.006903247	0.094133775
Lrrc16a	ENSMUSG00000021338	0.285892072	0.006912356	0.094151599
Efna5	ENSMUSG00000048915	0.329778776	0.006943155	0.094303796
Igsf9b	ENSMUSG00000034275	0.784192365	0.006944773	0.094303796
Zfp758	ENSMUSG00000044501	-0.540745946	0.006957974	0.094303796
Psmb3	ENSMUSG00000069744	-0.429278318	0.006962339	0.094303796
Btrc	ENSMUSG00000025217	0.22359196	0.006974458	0.094303796
Urm1	ENSMUSG00000069020	-0.307720287	0.006980173	0.094303796
Slc50a1	ENSMUSG00000027953	-0.169774467	0.006982829	0.094303796
Sec31b+Ndufb8+Gm20538	ENSMUSG00000091471+ENSMUSG00000025204+E	-0.354862507	0.007014026	0.094303796
Slc2a5	ENSMUSG00000028976	0.400605502	0.00701633	0.094303796
Npm1	ENSMUSG00000057113	-0.33528124	0.007024475	0.094303796
Zfp709	ENSMUSG00000056019	-0.401496444	0.007037126	0.094303796
Lrrc20	ENSMUSG00000037151	0.273855439	0.007038378	0.094303796
Pigyl	ENSMUSG00000010607	-0.355160798	0.007040484	0.094303796
Rbfox3	ENSMUSG00000025576	0.408580415	0.007065849	0.094303796
Trappc1	ENSMUSG00000049299	-0.24420423	0.007074378	0.094303796
Mrps14	ENSMUSG00000058267	-0.467742138	0.007092123	0.094303796
Qk	ENSMUSG00000062078	-0.357257623	0.007109048	0.094303796
Ptprn2	ENSMUSG00000056553	0.385619982	0.007110909	0.094303796
Egr1	ENSMUSG00000038418	0.679168338	0.007112277	0.094303796
Kcnh1	ENSMUSG00000058248	0.434720262	0.007117151	0.094303796
Plxnd1	ENSMUSG00000030123	0.625137857	0.007117198	0.094303796
Sri	ENSMUSG00000003161	-0.288557729	0.007153803	0.094303796
Cdh13	ENSMUSG00000031841	0.34743553	0.007159126	0.094303796
Sdhd	ENSMUSG00000000171	-0.227523379	0.007164462	0.094303796
Flot2	ENSMUSG00000061981	0.219526605	0.007165244	0.094303796
Ngdn	ENSMUSG00000022204	-0.355986635	0.007181505	0.094303796
Snx29	ENSMUSG00000071669	0.394182295	0.007188675	0.094303796
Amd1	ENSMUSG00000075232	-0.26143751	0.007190638	0.094303796
Trmt11	ENSMUSG00000019792	-0.440096157	0.007200124	0.094303796
Cfap36	ENSMUSG00000020462	-0.458144248	0.007200883	0.094303796
Mbp	ENSMUSG00000041607	-0.188670184	0.007208477	0.094303796
Zfp882+Zfp617	ENSMUSG00000089857+ENSMUSG00000066880	-0.30655245	0.007211738	0.094303796
Zfp316	ENSMUSG00000046658	0.392570043	0.007222325	0.094303796
Tap1	ENSMUSG00000037321	0.420131394	0.007233718	0.094303796
Nr1d2	ENSMUSG00000021775	-0.358489669	0.007241061	0.094303796
Snora65+Rpl12	ENSMUSG00000038900+ENSMUSG00000065124	-0.466861586	0.007255599	0.094303796
Arhgap17	ENSMUSG00000030766	0.431634773	0.00725593	0.094303796
Utp20	ENSMUSG00000004356	0.330401457	0.007257027	0.094303796
Parp14	ENSMUSG00000034422	0.437259487	0.007261317	0.094303796
Scaf4	ENSMUSG00000022983	0.344897323	0.007268278	0.094303796
Eif2s1	ENSMUSG00000021116	-0.24897623	0.00728542	0.094303796
Hmgxb3	ENSMUSG00000024622	0.45280895	0.007294	0.094303796
Dbpht2	ENSMUSG00000029878	-0.358363563	0.007302254	0.094303796
E130114P18Rik	ENSMUSG00000048747	-0.650164969	0.007303545	0.094303796
Pcnx	ENSMUSG00000021140	0.407434732	0.007305986	0.094303796
Zfp943	ENSMUSG00000053347	-0.46165991	0.007311908	0.094303796
Abhd3	ENSMUSG00000002475	-0.205072201	0.00731608	0.094303796
Mark4	ENSMUSG00000030397	0.51680346	0.007316141	0.094303796
Ktn1	ENSMUSG00000021843	-0.209479233	0.00733367	0.094303796
Cfl2	ENSMUSG00000062929	-0.336338989	0.007336034	0.094303796
Rmdn3	ENSMUSG00000070730	-0.213778962	0.007343713	0.094303796
Snx5	ENSMUSG00000027423	-0.346636643	0.007343766	0.094303796

Snhg6	ENSMUSG00000098234	-0.506952408	0.007350785	0.094303796
Anapc16	ENSMUSG00000020107	-0.240061386	0.007351136	0.094303796
Pde10a	ENSMUSG00000023868	0.328099078	0.007353321	0.094303796
Fam229b	ENSMUSG00000051736	-0.412838505	0.007372375	0.0944478
Tmem30a	ENSMUSG00000032328	-0.425507189	0.007393543	0.094592979
Hoxd3os1	ENSMUSG00000052371	-0.423492878	0.007399384	0.094592979
AI987944	ENSMUSG00000056383	-0.32142991	0.007421376	0.094678731
Rpl36al	ENSMUSG00000049751	-0.469172608	0.00742821	0.094678731
Rgs2	ENSMUSG00000026360	-0.276162764	0.007429629	0.094678731
Nkiras1	ENSMUSG00000021772	-0.236071257	0.00745362	0.09488427
Mrps10	ENSMUSG00000034729	-0.29725985	0.007491465	0.095093117
Cnpy2	ENSMUSG00000025381	-0.388334498	0.00749918	0.095093117
Rab18	ENSMUSG00000073639	-0.299868968	0.007507114	0.095093117
Zfp280d	ENSMUSG00000038535	-0.303512727	0.007513332	0.095093117
Gpr161	ENSMUSG00000040836	0.359855332	0.007532071	0.095093117
Cdo1	ENSMUSG00000033022	-0.344087028	0.007534185	0.095093117
Zfp759	ENSMUSG00000057396	-0.571141513	0.007537795	0.095093117
Ptp4a1	ENSMUSG00000026064	-0.328035624	0.007548354	0.095093117
Idh1	ENSMUSG00000025950	-0.172092401	0.0075498	0.095093117
Xrcc1	ENSMUSG00000051768	0.20033428	0.00755286	0.095093117
Slf1	ENSMUSG00000021597	-0.333890936	0.007556704	0.095093117
Eif2a	ENSMUSG00000027810	-0.160637728	0.007582042	0.09509471
Khnyn	ENSMUSG00000047153	0.445841454	0.007587175	0.09509471
Gm14326	ENSMUSG00000078862	-0.592486383	0.007609837	0.09509471
Mthfsl	ENSMUSG00000079427	-0.412457876	0.007643277	0.09509471
Coprs	ENSMUSG00000031458	-0.373347694	0.007649831	0.09509471
Rps7	ENSMUSG00000061477	-0.518238537	0.007652072	0.09509471
Dnajb9	ENSMUSG00000014905	-0.341761706	0.007654608	0.09509471
Smim3	ENSMUSG00000038059	-0.611697459	0.00766776	0.09509471
Zfp61	ENSMUSG00000050605	0.303723603	0.007670913	0.09509471
Bola2	ENSMUSG00000047721	-0.479370482	0.00769225	0.09509471
Apip	ENSMUSG00000010911	-0.240310717	0.007694412	0.09509471
Rims3	ENSMUSG00000032890	0.262769514	0.007704132	0.09509471
Tln2	ENSMUSG00000052698	0.549809	0.007709079	0.09509471
Crk	ENSMUSG00000017776	-0.302908421	0.007715131	0.09509471
Vti1b	ENSMUSG00000021124	-0.443248239	0.007720935	0.09509471
Bola3	ENSMUSG00000045160	-0.492353988	0.007724339	0.09509471
Mterf2	ENSMUSG00000049038	-0.402111822	0.007725182	0.09509471
Kcna2	ENSMUSG00000040724	0.30730757	0.007727372	0.09509471
Rnf157	ENSMUSG00000052949	0.450136572	0.007729521	0.09509471
Mir5117+Snord80+Gm2622	ENSMUSG00000077220+ENSMUSG00000065160+E	-0.458962102	0.007739842	0.09509471
Iqcb1	ENSMUSG00000022837	-0.354854934	0.007747397	0.09509471
Notch3	ENSMUSG00000038146	0.687446167	0.007754901	0.09509471
Rps16	ENSMUSG00000037563	-0.456742051	0.007761898	0.09509471
Mpc2	ENSMUSG00000026568	-0.334150092	0.007762412	0.09509471
Nipal3	ENSMUSG00000028803	0.378233866	0.007764818	0.09509471
Tpmt	ENSMUSG00000021376	-0.267558561	0.007772535	0.09509471
Prkra	ENSMUSG00000002731	-0.173179747	0.007778514	0.09509471
Pla2g16	ENSMUSG00000060675	-0.299190566	0.007799352	0.09509471
Gtf2h5	ENSMUSG00000034345	-0.394941524	0.007815902	0.09509471
Tbca	ENSMUSG00000042043	-0.527120607	0.007819986	0.09509471
Igsf3	ENSMUSG00000042035	0.504737844	0.007831607	0.09509471
Syne3	ENSMUSG00000054150	0.457779998	0.007832337	0.09509471
Ptprt	ENSMUSG00000053141	0.413840915	0.007835541	0.09509471
Tpt1	ENSMUSG00000060126	-0.440633382	0.007838705	0.09509471
Disp1	ENSMUSG00000030768	0.363603554	0.007840217	0.09509471
Gm14303	ENSMUSG00000081344	-0.549503349	0.007840507	0.09509471
0610012G03Rik	ENSMUSG00000107002+ENSMUSG00000096232	-0.351528052	0.007869058	0.095345169
Hist2h2be	ENSMUSG00000068854	-0.353949925	0.007890572	0.095504767

Gmfb	ENSMUSG00000062014	-0.400841994	0.007899713	0.095504767
Ptprj	ENSMUSG00000025314	0.33602697	0.007905971	0.095504767
Caskin2	ENSMUSG00000034471	0.460098643	0.007920897	0.09556722
Rps27a	ENSMUSG00000020460	-0.508305531	0.007927332	0.09556722
Skp1a	ENSMUSG00000036309	-0.259714582	0.007941329	0.09556722
Sdhaf3	ENSMUSG00000042505	-0.393191376	0.007943907	0.09556722
Cyfip2	ENSMUSG00000020340	0.423009056	0.007956356	0.09556722
Ablim1	ENSMUSG00000025085	0.278662417	0.007958656	0.09556722
Ginm1	ENSMUSG00000040006	-0.304782891	0.007967001	0.095572332
Snrk	ENSMUSG00000038145	0.333827835	0.007980622	0.095640662
Dock1	ENSMUSG00000058325	0.410992111	0.007995848	0.095673684
Zswim1	ENSMUSG00000017764	0.341931879	0.008012741	0.095673684
D3Ertd751e	ENSMUSG00000025766	-0.446073851	0.008013694	0.095673684
Ccnd2	ENSMUSG00000000184	0.231112756	0.008015089	0.095673684
Ago1	ENSMUSG00000041530	0.375555602	0.008105701	0.096266764
Chd6	ENSMUSG00000057133	0.316176253	0.008109687	0.096266764
Triqk	ENSMUSG00000055963	-0.551192574	0.00812178	0.096266764
Gstk1	ENSMUSG00000029864	-0.25915537	0.008133891	0.096266764
BC003331	ENSMUSG00000006010	-0.398527683	0.008136573	0.096266764
Psma4	ENSMUSG00000032301	-0.408309987	0.008138453	0.096266764
Tstd2	ENSMUSG00000035495	0.255199447	0.008138548	0.096266764
Mrpl12	ENSMUSG00000039640	-0.352024092	0.008144674	0.096266764
Sema6d	ENSMUSG00000027200	0.285532091	0.008155774	0.096266764
Zmat2	ENSMUSG00000001383	-0.345412629	0.008165313	0.096266764
Ndfip2	ENSMUSG00000053253	-0.237805363	0.00816676	0.096266764
Hspb8	ENSMUSG00000041548	-0.190615607	0.008169708	0.096266764
Mar-09	ENSMUSG00000040502	0.322736764	0.008175705	0.096266764
Exoc5	ENSMUSG00000061244	-0.391440259	0.008178091	0.096266764
Snord50a+Snord50b+Snhg5	ENSMUSG00000094263+ENSMUSG00000094179+E	-0.369259607	0.00818443	0.096266764
Kcna1	ENSMUSG00000047976	0.369397378	0.008218233	0.096519187
Lyrm7	ENSMUSG00000020268	-0.367653493	0.00823051	0.096519187
Zfp654	ENSMUSG00000047141	-0.491647824	0.008230895	0.096519187
Wdr12	ENSMUSG00000026019	-0.18553668	0.008237882	0.096519187
Lrba	ENSMUSG00000028080	0.271503603	0.008273357	0.096803752
Anapc10	ENSMUSG00000036977	-0.54197974	0.008296215	0.096803752
Slc35f2	ENSMUSG00000042195	0.283445725	0.008299649	0.096803752
Mir7079+Snord68+Rpl13	ENSMUSG00000098550+ENSMUSG00000000740+E	-0.38519424	0.008300615	0.096803752
Icmt	ENSMUSG00000039662	0.25543122	0.008302277	0.096803752
Coro1c	ENSMUSG00000004530	0.284210682	0.008310896	0.096810704
Sec62	ENSMUSG00000027706	-0.195756134	0.008331446	0.096876858
Stox2	ENSMUSG00000038143	0.289306275	0.008347424	0.096876858
Rnf213	ENSMUSG00000070327	0.644051584	0.008361378	0.096876858
Triap1	ENSMUSG00000029535	-0.288577978	0.008388347	0.096876858
Tmem263	ENSMUSG00000060935	-0.326999607	0.008390396	0.096876858
Gm10076	ENSMUSG00000060143	-0.613100347	0.008393683	0.096876858
Leo1	ENSMUSG00000042487	0.286912425	0.008396347	0.096876858
Kctd4	ENSMUSG00000046523	-0.518967714	0.008398293	0.096876858
B230118H07Rik+Rag1	ENSMUSG00000061311+ENSMUSG00000027165	-0.514314517	0.008420089	0.096876858
Lingo4	ENSMUSG00000044505	0.313748375	0.00843479	0.096876858
Piezo1	ENSMUSG00000014444	0.574097871	0.00843623	0.096876858
Tbc1d15	ENSMUSG00000020130	-0.260824915	0.008446322	0.096876858
Gpatch1	ENSMUSG00000063808	0.18628884	0.008454938	0.096876858
Ddx50	ENSMUSG00000020076	-0.274544371	0.008456318	0.096876858
Mt3	ENSMUSG00000031760	-0.324984963	0.008465293	0.096876858
Lzts1	ENSMUSG00000036306	0.389113682	0.008467001	0.096876858
Ppil3	ENSMUSG00000026035	-0.42168403	0.0084729	0.096876858
Gng5	ENSMUSG00000068523	-0.488760067	0.008497514	0.096876858
Mrpl27	ENSMUSG00000024414	-0.456802317	0.008500162	0.096876858
Scarb1	ENSMUSG00000037936	0.270104589	0.008500836	0.096876858

Fam53b	ENSMUSG00000030956	0.342770342	0.0085064	0.096876858
Atox1	ENSMUSG00000018585	-0.288739543	0.008509	0.096876858
Zdhhc20	ENSMUSG00000021969	-0.358059499	0.008533882	0.096876858
0610037L13Rik	ENSMUSG00000028608	-0.246103668	0.008539964	0.096876858
Trim23	ENSMUSG00000021712	-0.442502715	0.008539968	0.096876858
Sdhaf4	ENSMUSG00000026154	-0.512752816	0.008544152	0.096876858
Cgnl1	ENSMUSG00000032232	0.500244599	0.008556333	0.096876858
Mir1668+Adgre5	ENSMUSG00000002885+ENSMUSG00000098465	0.270200409	0.008556811	0.096876858
Pecam1	ENSMUSG00000020717	0.487344092	0.008565603	0.096876858
Gm14306+Gm14305	ENSMUSG00000078878+ENSMUSG00000078879	-0.503873382	0.008574477	0.096876858
Gabbr2	ENSMUSG00000039809	0.500971097	0.008582167	0.096876858
Serp1	ENSMUSG00000027808	-0.339808425	0.008604409	0.096876858
1700001L19Rik	ENSMUSG00000021534	-0.518612803	0.008609133	0.096876858
Mknk1	ENSMUSG00000028708	0.222722551	0.008610368	0.096876858
Calca	ENSMUSG00000030669	-0.492284286	0.00861461	0.096876858
Myo16	ENSMUSG00000039057	0.584554394	0.008619697	0.096876858
Snrnp200	ENSMUSG00000003660	0.397885659	0.008622429	0.096876858
Rpl37a	ENSMUSG00000046330	-0.404587924	0.008625533	0.096876858
Cmpk1	ENSMUSG00000028719	-0.349809528	0.008629651	0.096876858
Cyb5a	ENSMUSG00000024646	-0.46264081	0.008659614	0.097063508
Atp6v0b	ENSMUSG00000033379	-0.221735892	0.008669447	0.097063508
Vamp8	ENSMUSG00000050732	-0.553661818	0.00867086	0.097063508
Tnks	ENSMUSG00000031529	0.320068459	0.008678449	0.097063508
Acox3	ENSMUSG00000029098	0.257892371	0.008714317	0.097185128
Ccdc3	ENSMUSG00000026676	-0.292102963	0.00871462	0.097185128
Arhgap35	ENSMUSG00000058230	0.330111377	0.008714707	0.097185128
Fam45a	ENSMUSG00000024993	-0.247232805	0.008723206	0.097185128
Nynrin	ENSMUSG00000075592	0.540014078	0.008734244	0.097185128
Memo1	ENSMUSG00000058704	-0.314292144	0.008751496	0.097185128
Socs6	ENSMUSG00000056153	-0.425618267	0.008752201	0.097185128
Ndufb9	ENSMUSG00000022354	-0.438139995	0.008753748	0.097185128
Rps27l	ENSMUSG00000036781	-0.594658364	0.008764892	0.09721941
Adcy1	ENSMUSG00000020431	0.52319725	0.008787915	0.097303492
A330074K22Rik	ENSMUSG00000097960	0.44223981	0.008788599	0.097303492
Uchl3	ENSMUSG00000022111	-0.480096058	0.008807939	0.097366052
Cbl	ENSMUSG00000034342	0.516674409	0.008824618	0.097366052
Katnb1	ENSMUSG00000031787	0.285996667	0.008843563	0.097366052
Rpl9	ENSMUSG00000047215	-0.401725125	0.008846378	0.097366052
Mrps36	ENSMUSG00000061474	-0.338776091	0.008847509	0.097366052
Sec61g	ENSMUSG00000078974	-0.488737073	0.008849277	0.097366052
Pcdh1	ENSMUSG00000051375	0.697612683	0.008850726	0.097366052
Luzp1	ENSMUSG00000001089	0.223310409	0.008861167	0.097392135
Brinp1	ENSMUSG00000028351	0.205039155	0.008872836	0.097413811
Rsl1d1	ENSMUSG00000005846	-0.212770279	0.00888225	0.097413811
Hnrnpc	ENSMUSG00000060373	-0.2419328	0.008889691	0.097413811
Nr1h3+Gm13783	ENSMUSG00000086206+ENSMUSG00000002108	-0.340375854	0.008896283	0.097413811
Ndufb6	ENSMUSG00000071014	-0.457589989	0.008913805	0.097413811
Ska2	ENSMUSG00000020492	-0.46881263	0.008917051	0.097413811
Abl2	ENSMUSG00000026596	0.411180801	0.008921178	0.097413811
Lrtm2	ENSMUSG00000055003	0.404257677	0.008927716	0.097413811
Slc19a1	ENSMUSG00000001436	0.299590212	0.008947305	0.097459368
Itfg1	ENSMUSG00000031703	-0.292499481	0.008949724	0.097459368
Sstr2	ENSMUSG00000047904	0.309028129	0.008956119	0.097459368
Yme1l1	ENSMUSG00000026775	-0.343024206	0.008975999	0.097587712
Erc1	ENSMUSG00000030172	0.331200476	0.008986766	0.097616824
4933404O12Rik	ENSMUSG00000097908	0.313458077	0.009005379	0.097679635
Sv2c	ENSMUSG00000051111	0.488969298	0.009008737	0.097679635
Zfp46	ENSMUSG00000051351	0.30895704	0.009065105	0.098201154
Ttbk1	ENSMUSG00000015599	0.589796336	0.009073757	0.098201154

Cdc123	ENSMUSG00000039128	-0.289212392	0.009084545	0.098201154
Tmem39b	ENSMUSG00000053730	0.391275107	0.009099701	0.098201154
Ctxn2	ENSMUSG00000074872	-0.546765773	0.0091044	0.098201154
Chpt1	ENSMUSG00000060002	-0.563225594	0.009112328	0.098201154
Plxna4	ENSMUSG00000029765	0.604199816	0.009113796	0.098201154
Adsl	ENSMUSG00000022407	0.23577122	0.009144283	0.098441758
Specc1l	ENSMUSG00000033444	0.241661067	0.009166537	0.098496834
Golgb1	ENSMUSG00000034243	0.321712819	0.00917625	0.098496834
Evc	ENSMUSG00000029122	0.273128604	0.009181303	0.098496834
Timm10	ENSMUSG00000027076	-0.360119911	0.009182047	0.098496834
Parvb	ENSMUSG00000022438	0.353246316	0.009219725	0.098759207
Nebl	ENSMUSG00000053702	-0.424039779	0.009225689	0.098759207
Golga2	ENSMUSG00000002546	0.325284924	0.009243876	0.098759207
Timm9	ENSMUSG00000021079	-0.267826595	0.009252216	0.098759207
Ezr	ENSMUSG00000052397	0.282365723	0.009255126	0.098759207
Stxbp3	ENSMUSG00000027882	-0.274455228	0.009259488	0.098759207
Gm29514	ENSMUSG00000099964	-0.439168119	0.00926379	0.098759207
Col18a1	ENSMUSG00000001435	0.387234084	0.009297651	0.098872291
Ppp6c	ENSMUSG00000026753	-0.260578327	0.009306946	0.098872291
Rpl15	ENSMUSG00000012405	-0.403912836	0.009314361	0.098872291
Arel1	ENSMUSG00000042350	0.273091542	0.00932828	0.098872291
Spred1	ENSMUSG00000027351	-0.345609656	0.009359601	0.098872291
Nkain2	ENSMUSG00000069670	-0.288405131	0.009362738	0.098872291
Arpp19	ENSMUSG00000007656	-0.275555778	0.009382885	0.098872291
Anapc13	ENSMUSG00000035048	-0.513077785	0.00939015	0.098872291
Nedd4	ENSMUSG00000032216	0.227441232	0.009406951	0.098872291
Mrps33	ENSMUSG00000029918	-0.465480083	0.009406958	0.098872291
Slc35c1	ENSMUSG00000049922	0.379271096	0.009409612	0.098872291
Clasp1	ENSMUSG00000064302	0.419483221	0.009411998	0.098872291
Pfdn2	ENSMUSG00000006412	-0.412973485	0.009414029	0.098872291
Asnsd1+Gm28551+Gm188(	ENSMUSG00000026095+ENSMUSG00000101838+E	-0.340067547	0.009419572	0.098872291
Cebpzos	ENSMUSG00000062691	-0.517517102	0.009420576	0.098872291
Dram2	ENSMUSG00000027900	-0.446672223	0.009421333	0.098872291
Minos1	ENSMUSG00000050608	-0.387295896	0.009428347	0.098872291
Tmem14a	ENSMUSG00000025933	-0.430370079	0.009433109	0.098872291
Mblac2	ENSMUSG00000051098	-0.276349455	0.00943773	0.098872291
Fam120aos	ENSMUSG00000097059	-0.475722731	0.00944207	0.098872291
Sesn2	ENSMUSG00000028893	0.424030087	0.009449261	0.098872291
Snora7a+Rpl32	ENSMUSG00000064563+ENSMUSG00000057841	-0.452748874	0.009454642	0.098872291
Abracl	ENSMUSG00000078453	-0.488361052	0.009476315	0.099013133
Hectd1	ENSMUSG00000035247	0.328080115	0.009506135	0.099065452
Ptges3	ENSMUSG00000071072	-0.215514218	0.009509107	0.099065452
Tm4sf1	ENSMUSG00000027800	-0.349719538	0.009514979	0.099065452
Mrpl41	ENSMUSG00000036850	-0.39281089	0.009521476	0.099065452
Cd302	ENSMUSG00000060703	-0.472376669	0.009522367	0.099065452
Naa50	ENSMUSG00000022698	-0.381116953	0.00954102	0.099090212
Patl1	ENSMUSG00000046139	0.22322602	0.009545718	0.099090212
Dnajc21	ENSMUSG00000044224	-0.181600224	0.009568881	0.099090212
Sec11c	ENSMUSG00000024516	-0.408214876	0.009596422	0.099090212
Tmem9b	ENSMUSG00000031021	-0.270998751	0.009598132	0.099090212
Snrpd3	ENSMUSG00000020180	-0.285001424	0.009609638	0.099090212
Ift20	ENSMUSG00000001105	-0.29377274	0.009614122	0.099090212
Emc4	ENSMUSG00000027131	-0.286583119	0.009615822	0.099090212
Tomm7	ENSMUSG00000028998	-0.486033228	0.009621823	0.099090212
Mir7011+Sema4a	ENSMUSG00000028064+ENSMUSG00000098655	0.450624607	0.009627812	0.099090212
Loxl3	ENSMUSG00000000693	0.482063777	0.009629624	0.099090212
Trpm3	ENSMUSG00000052387	0.275280068	0.009630358	0.099090212
Zfpm1	ENSMUSG00000049577	0.501713107	0.00963149	0.099090212
Gm28403+Pign	ENSMUSG00000101214+ENSMUSG00000056536	-0.338762078	0.009648481	0.099180382

2010320M18Rik	ENSMUSG00000100691	-0.633937543	0.009656691	0.099180382
Acss3	ENSMUSG00000035948	-0.55929848	0.009673712	0.099189756
Sidt1	ENSMUSG00000022696	0.405705772	0.009674042	0.099189756
2010315B03Rik	ENSMUSG00000074829	-0.329511711	0.009687823	0.099220966
Tmem60	ENSMUSG00000045435	-0.34116387	0.009697759	0.099220966
Gm24158+Gm11613	ENSMUSG00000093004+ENSMUSG00000085586	-0.277903163	0.009706237	0.099220966
Bloc1s2	ENSMUSG00000057506	-0.417350716	0.009709974	0.099220966
Ston2	ENSMUSG00000020961	0.453818023	0.009720233	0.099241774
AI504432	ENSMUSG00000056145	-0.475605611	0.009734208	0.099246363
Mrps15	ENSMUSG00000028861	-0.290816957	0.009748431	0.099246363
Echs1	ENSMUSG00000025465	-0.242701633	0.009753224	0.099246363
Usp13	ENSMUSG00000056900	0.42698559	0.009753579	0.099246363
D8Ertd738e	ENSMUSG00000019362	-0.366365629	0.009772308	0.099311499
Gm24523+Rpl30	ENSMUSG00000058600+ENSMUSG00000065899	-0.485403095	0.009786275	0.099311499
Nphp1	ENSMUSG00000027378	-0.266677136	0.009789228	0.099311499
Gpr75+Asb3	ENSMUSG00000043999+ENSMUSG00000020305	-0.258009104	0.009792897	0.099311499
Suz12	ENSMUSG00000017548	-0.343015727	0.009802681	0.099326665
Pigf	ENSMUSG00000024145	-0.407387129	0.009820145	0.099326665
Slc6a17	ENSMUSG00000027894	0.438083548	0.00982946	0.099326665
Rpl17	ENSMUSG00000062328	-0.406770255	0.009838567	0.099326665
Commd4	ENSMUSG00000032299	-0.216493674	0.009844666	0.099326665
Ythdf3	ENSMUSG00000047213	-0.367334568	0.009848415	0.099326665
Polr2h	ENSMUSG00000021018	-0.392609003	0.009861605	0.099326665
Srgap3	ENSMUSG00000030257	0.458487961	0.009874443	0.099326665
Zfp942	ENSMUSG00000071267	-0.455361719	0.009885084	0.099326665
Atp6v1h	ENSMUSG00000033793	-0.193672627	0.009889036	0.099326665
	ENSMUSG00000106744	-0.258619076	0.009894124	0.099326665
Nsa2	ENSMUSG00000060739	-0.420897268	0.009902712	0.099326665
Ephb4	ENSMUSG00000029710	0.440063733	0.009907373	0.099326665
Cux1	ENSMUSG00000029705	0.171014351	0.009909621	0.099326665
Snapin	ENSMUSG00000001018	-0.182215982	0.009925818	0.099406454
Tmx3	ENSMUSG00000024614	-0.469036472	0.009975708	0.099704175
Fmnl3	ENSMUSG00000023008	0.259589944	0.009980236	0.099704175
Naa20	ENSMUSG00000002728	-0.51319814	0.009980332	0.099704175
Chmp2b	ENSMUSG00000004843	-0.174037555	0.010000416	0.099748816
Wbp1	ENSMUSG00000030035	-0.359970508	0.010010436	0.099748816
Tmbim4	ENSMUSG00000020225	-0.289248132	0.010012641	0.099748816
Neto2	ENSMUSG00000036902	-0.427519401	0.010017862	0.099748816
Aqp4	ENSMUSG00000024411	-0.400928925	0.010027645	0.099761723
Mrpl50	ENSMUSG00000044018	-0.34753524	0.01004237	0.099761723
Gm20498+Synj2bp+Cox16	ENSMUSG00000021139+ENSMUSG00000090935+E	-0.446208148	0.01006364	0.099761723
Gnpnat1	ENSMUSG00000037722	-0.437360295	0.010069579	0.099761723
Rel	ENSMUSG00000020275	0.293380097	0.010073223	0.099761723
Ndufaf5	ENSMUSG00000027384	-0.24062438	0.010082407	0.099761723
Tcof1	ENSMUSG00000024613	0.551743579	0.010092786	0.099761723
Tsc22d2	ENSMUSG00000027806	-0.240677241	0.010095619	0.099761723
Zfp35	ENSMUSG00000063281	-0.22839761	0.010098174	0.099761723
Hmgcll1	ENSMUSG00000007908	-0.279827606	0.010101825	0.099761723
Ppp1cb+Gm43312	ENSMUSG00000107197+ENSMUSG00000014956	-0.344688252	0.010120741	0.099778505
Vps29	ENSMUSG00000029462	-0.382686494	0.010141731	0.099778505
Pcnx2	ENSMUSG00000060212	0.600495238	0.010145087	0.099778505
Eef1akmt1	ENSMUSG00000021951	-0.281523756	0.010152915	0.099778505
Sccpdh	ENSMUSG00000038936	-0.189334735	0.010155711	0.099778505
Zfp85	ENSMUSG00000058331	-0.523694286	0.010156813	0.099778505
6430548M08Rik	ENSMUSG00000031824	0.350747184	0.010162156	0.099778505
S100a16	ENSMUSG00000074457	-0.343222404	0.010169669	0.099778505
Tmco1	ENSMUSG00000052428	-0.295779265	0.010181368	0.099812145
Upf1	ENSMUSG00000058301	0.542177416	0.010206123	0.099903519
Tbc1d16	ENSMUSG00000039976	0.322776851	0.010215552	0.099903519

Gabra4	ENSMUSG00000029211	-0.416359299	0.010219192	0.099903519
Scpep1	ENSMUSG00000000278	0.303319041	0.010227535	0.099903519
Stk17b	ENSMUSG00000026094	-0.416149581	0.010235383	0.099903519
Ece1	ENSMUSG00000057530	0.497946701	0.010269903	0.099903519
2810428I15Rik	ENSMUSG00000058833	-0.344817118	0.010279214	0.099903519
Slc5a7	ENSMUSG00000023945	-0.294329184	0.010282575	0.099903519
Azin1	ENSMUSG00000037458	-0.325824959	0.010285892	0.099903519
Zc3h4	ENSMUSG00000059273	0.484085059	0.010292271	0.099903519
Gm27937+Gm27517+Gm27	ENSMUSG00000056579+ENSMUSG00000098503+E	-0.294745302	0.010296205	0.099903519
Zfp386	ENSMUSG00000042063	-0.423291141	0.010303595	0.099903519
9230112E08Rik+Col20a1	ENSMUSG00000070461+ENSMUSG00000016356	0.443365985	0.010305032	0.099903519
Hpf1	ENSMUSG00000038005	-0.338236873	0.010314713	0.099903519
Itgb8	ENSMUSG00000025321	-0.585769671	0.010314864	0.099903519
Pnpla8	ENSMUSG00000036257	-0.342924428	0.010333286	0.099948428
Papd7	ENSMUSG00000034575	0.316555522	0.01034841	0.099948428
Borcs7	ENSMUSG00000062376	-0.271923078	0.010373234	0.099948428
Mef2d	ENSMUSG00000001419	0.446606663	0.010373713	0.099948428
Eps15	ENSMUSG00000028552	-0.306047333	0.010384844	0.099948428
Lgalsl	ENSMUSG00000042363	-0.314202272	0.010388505	0.099948428
Tiam2	ENSMUSG00000023800	0.405842909	0.010402051	0.099948428
Megf9	ENSMUSG00000039270	-0.266918222	0.010402482	0.099948428
Mrps31	ENSMUSG00000031533	-0.266055364	0.010422995	0.099948428
	ENSMUSG00000057924	-0.600203136	0.010437506	0.099948428
Fam172a	ENSMUSG00000064138	-0.249234186	0.010438573	0.099948428
Mydgf	ENSMUSG00000019579	-0.342730161	0.010442335	0.099948428
Btbd9	ENSMUSG00000062202	0.347298287	0.010470175	0.099948428
Mmp15	ENSMUSG00000031790	0.576923613	0.010499891	0.099948428
Apba2	ENSMUSG00000030519	0.342273826	0.010505006	0.099948428
Prpf8	ENSMUSG00000020850	0.545741406	0.010515611	0.099948428
Rps17	ENSMUSG00000061787	-0.46040188	0.010522065	0.099948428
Vps50	ENSMUSG00000001376	-0.198595255	0.010524926	0.099948428
Cox7a2l	ENSMUSG00000024248	-0.353035581	0.01052878	0.099948428
Dcun1d1	ENSMUSG00000027708	-0.47244048	0.010541411	0.099948428
Galnt10	ENSMUSG00000020520	0.236804616	0.010547632	0.099948428
Prkca	ENSMUSG00000050965	0.212791864	0.010554561	0.099948428
Nab1	ENSMUSG00000002881	-0.223089286	0.010555834	0.099948428
Anapc11	ENSMUSG00000025135	-0.236326898	0.010559159	0.099948428
Sp3	ENSMUSG00000027109	-0.396748574	0.010562546	0.099948428
Lasp1	ENSMUSG00000038366	0.275029034	0.010567041	0.099948428
Snrnp27	ENSMUSG00000001158	-0.486932859	0.010569795	0.099948428
Zfp646	ENSMUSG00000049739	0.425088192	0.010570964	0.099948428
Gm42671+Golph3l	ENSMUSG00000105460+ENSMUSG00000046519	-0.226272737	0.010589646	0.099948428
Dnajc19	ENSMUSG00000027679	-0.496300966	0.010595074	0.099948428
Lrig1	ENSMUSG00000030029	0.324712933	0.010596426	0.099948428
St7l	ENSMUSG00000045576	-0.238351159	0.010620294	0.099948428
Srsf3	ENSMUSG00000071172	-0.302791504	0.010625401	0.099948428
Pdcl3	ENSMUSG00000026078	-0.45858396	0.010636789	0.099948428
B230219D22Rik	ENSMUSG00000045767	-0.342525064	0.010637993	0.099948428
Ptpn21	ENSMUSG00000021009	0.308618254	0.010641264	0.099948428
Mrpl35	ENSMUSG00000052962	-0.279879124	0.010642963	0.099948428
Psmg4	ENSMUSG00000071451	-0.494986102	0.010645666	0.099948428
Gm27597+Gm11629+Rpl19	ENSMUSG00000098992+ENSMUSG00000017404+E	-0.384978792	0.010649914	0.099948428
Rab39	ENSMUSG00000055069	-0.499050339	0.010655933	0.099948428
S100a1	ENSMUSG00000044080	-0.446613507	0.010662119	0.099948428
Usp1	ENSMUSG00000028560	-0.315862248	0.010669544	0.099948428
Glrx	ENSMUSG00000021591	-0.335621094	0.010675632	0.099948428

Supplementary Table 2 Expression gene list for 12 months

Humanised mutant FUS drives progressive motor neuron degeneration without aggregation in 'FUSDelta14' knockin mice

Devoy et al. 2017

Supplementary Table 3 GO term pathway enrichment analysis of genes dysregulated at 12 months of age in FUS Delta14 spinal cord.

category	over_represented_pvalue	under_represented_pvalue	numDEInCat	Down	Up	numInCat	term	ontology	Category
GO:0044429	3.90E-20	1	77	76	1	553	mitochondrial part	CC	1
GO:0070469	3.13E-19	1	24	24	0	53	respiratory chain	CC	1
GO:0005743	4.95E-19	1	55	54	1	316	mitochondrial inner membrane	CC	1
GO:0005739	1.52E-18	1	134	126	8	1426	mitochondrion	CC	1
GO:0005746	2.21E-18	1	22	22	0	46	mitochondrial respiratory chain	CC	1
GO:0044455	2.46E-17	1	32	32	0	118	mitochondrial membrane part	CC	1
GO:0005740	6.67E-17	1	63	62	1	447	mitochondrial envelope	CC	1
GO:0031966	2.00E-16	1	60	59	1	420	mitochondrial membrane	CC	1
GO:0005747	4.11E-14	1	16	16	0	32	mitochondrial respiratory chain complex I	CC	1
GO:0030964	4.11E-14	1	16	16	0	32	NADH dehydrogenase complex	CC	1
GO:0045271	4.11E-14	1	16	16	0	32	respiratory chain complex I	CC	1
GO:1990204	1.79E-12	1	20	20	0	65	oxidoreductase complex	CC	1
GO:0045259	9.35E-09	1	9	9	0	17	proton-transporting ATP synthase complex	CC	1
GO:0009055	1.65E-08	0.999999998	14	14	0	50	electron carrier activity	MF	1
GO:0015078	2.43E-08	0.999999997	16	16	0	68	hydrogen ion transmembrane transporter activity	MF	1
GO:0005753	6.01E-08	0.999999998	8	8	0	15	mitochondrial proton-transporting ATP synthase complex	CC	1
GO:0016469	2.15E-07	0.99999998	11	11	0	36	proton-transporting two-sector ATPase complex	CC	1
GO:0055114	3.90E-07	1	62	57	5	748	oxidation-reduction process	BP	1
GO:0004129	6.04E-07	0.999999967	8	8	0	19	cytochrome-c oxidase activity	MF	1
GO:0015002	6.04E-07	0.999999967	8	8	0	19	heme-copper terminal oxidase activity	MF	1
GO:0016676	6.04E-07	0.999999967	8	8	0	19	oxidoreductase activity, acting on a heme group of donors, oxygen as acceptor	MF	1
GO:0016675	9.69E-07	0.999999943	8	8	0	20	oxidoreductase activity, acting on a heme group of donors	MF	1
GO:0045263	1.19E-06	0.99999997	6	6	0	10	proton-transporting ATP synthase complex, coupling factor F(o)	CC	1
GO:0006119	1.22E-06	0.999999903	9	9	0	27	oxidative phosphorylation	BP	1
GO:0042773	2.31E-06	0.999999884	7	7	0	16	ATP synthesis coupled electron transport	BP	1
GO:0033177	3.78E-06	0.99999979	7	7	0	17	proton-transporting two-sector ATPase complex, proton-transporting domain	CC	1
GO:0008137	5.95E-06	0.999999636	7	7	0	18	NADH dehydrogenase (ubiquinone) activity	MF	1
GO:0050136	5.95E-06	0.999999636	7	7	0	18	NADH dehydrogenase (quinone) activity	MF	1
GO:0000276	7.68E-06	0.999999828	5	5	0	8	mitochondrial proton-transporting ATP synthase complex, coupling factor F(o)	CC	1
GO:0003954	9.07E-06	0.999999396	7	7	0	19	NADH dehydrogenase activity	MF	1
GO:0016655	1.30E-05	0.999998782	8	8	0	27	oxidoreductase activity, acting on NAD(P)H, quinone or similar compound as acceptor	MF	1
GO:0022900	1.34E-05	0.999998457	9	9	0	35	electron transport chain	BP	1
GO:0042775	1.47E-05	0.999999253	6	6	0	14	mitochondrial ATP synthesis coupled electron transport	BP	1
GO:0070069	3.21E-05	0.999998806	5	5	0	10	cytochrome complex	CC	1
GO:0005761	4.78E-05	0.999992342	10	10	0	50	mitochondrial ribosome	CC	1
GO:0005759	9.93E-05	0.999969732	18	18	0	151	mitochondrial matrix	CC	1
GO:0044391	1.96E-13	1	26	26	0	104	ribosomal subunit	CC	2
GO:0005840	3.10E-13	1	32	32	0	161	ribosome	CC	2
GO:0003735	5.33E-12	1	24	24	0	101	structural constituent of ribosome	MF	2
GO:0022626	2.35E-10	1	18	18	0	66	cytosolic ribosome	CC	2

GO:0030529	6.74E-10	1	53	51	2	493	ribonucleoprotein complex	CC	2
GO:0015935	3.21E-09	1	15	15	0	52	small ribosomal subunit	CC	2
GO:0022627	6.33E-09	1	12	12	0	33	cytosolic small ribosomal subunit	CC	2
GO:0015934	1.39E-05	0.999997861	11	11	0	53	large ribosomal subunit	CC	2
GO:0006412	1.61E-05	0.999993454	35	35	0	373	translation	BP	2
GO:0000313	4.78E-05	0.999992342	10	10	0	50	organellar ribosome	CC	2
GO:0005839	5.95E-06	0.999999636	7	7	0	18	proteasome core complex	CC	3
GO:0019773	7.68E-06	0.999999828	5	5	0	8	proteasome core complex, alpha-subunit complex	CC	3
GO:0004298	1.34E-05	0.999999031	7	7	0	20	threonine-type endopeptidase activity	MF	3
GO:0070003	1.34E-05	0.999999031	7	7	0	20	threonine-type peptidase activity	MF	3
GO:0070062	1.96E-09	1	147	117	30	2137	extracellular vesicular exosome	CC	4
GO:0043230	2.15E-09	1	147	117	30	2140	extracellular organelle	CC	4
GO:0065010	2.15E-09	1	147	117	30	2140	extracellular membrane-bounded organelle	CC	4
GO:0031982	5.04E-08	1	174	134	40	2783	vesicle	CC	5
GO:0031988	8.20E-08	1	163	127	36	2583	membrane-bounded vesicle	CC	5
GO:0005198	2.08E-06	0.999999241	35	28	7	340	structural molecule activity	MF	6
GO:0003723	0.000106434	0.999936287	83	74	9	1270	RNA binding	MF	6
GO:0045116	0.000111545	0.999997012	4	4	0	7	protein neddylation	BP	6
GO:0019866	6.81E-19	1	57	55	2	340	organelle inner membrane	CC	7
GO:0031967	2.50E-14	1	76	71	5	686	organelle envelope	CC	7
GO:0031975	3.14E-14	1	76	71	5	689	envelope	CC	7
GO:0031090	6.92E-08	1	97	88	9	1317	organelle membrane	CC	7
GO:0044444	3.05E-15	1	324	275	49	5259	cytoplasmic part	CC	8
GO:0043227	6.35E-12	1	467	365	102	8837	membrane-bounded organelle	CC	8
GO:0005737	3.03E-11	1	424	340	84	7880	cytoplasm	CC	8
GO:0044446	2.58E-10	1	244	207	37	3996	intracellular organelle part	CC	8
GO:0044422	8.66E-10	1	249	211	38	4150	organelle part	CC	8
GO:0043226	9.72E-10	1	489	381	108	9610	organelle	CC	8
GO:0043231	3.43E-09	1	414	332	82	7875	intracellular membrane-bounded organelle	CC	8
GO:0043229	7.98E-09	1	449	360	89	8747	intracellular organelle	CC	8
GO:0044421	9.02E-08	1	173	135	38	2787	extracellular region part	CC	8
GO:0032991	1.19E-07	1	217	176	41	3702	macromolecular complex	CC	8
GO:0005576	1.26E-07	1	191	147	44	3167	extracellular region	CC	8
GO:0044424	2.32E-07	1	494	392	102	10026	intracellular part	CC	8
GO:0044445	1.08E-06	0.999999736	21	21	0	144	cytosolic part	CC	8
GO:0005622	2.31E-06	0.999999068	497	394	103	10208	intracellular	CC	8

Supplementary Table 3 GO term pathway enrichment analysis of genes dysregulated at 12 months of age in FUS Delta14 spinal cord.
