# Supplementary material for: Humanized mutant FUS drives progressive motor neuron degeneration without aggregation in ‘FUSDelta14’ knockin mice
Source: Brain. 2017 Oct 7;140(11):2797–805. doi: 10.1093/brain/awx248 (PMC5841203; doi:10.1093/brain/awx248)
Supplement: Supplementary Tables S4 to S10 [file brain-2017-00604-file009_awx248.pdf]

Humanised mutant FUS drives progressive motor neuron degeneration without aggregation in ‘FUSDelta14’ knockin mice

Devoy et al. 2017

Supplementary Table 4 Gene Set Enrichment Analysis (GSEA) aummary of gene lists dysregulated at 12 months of age in FUS Delta14 spinal corc

NAME	GS<br>follow link to MSigDB	GS DETAILS	SIZE	ES	NES	NOM p-val	FDR q-val	FWER p-val	RANK AT MAX	LEADING EDGE
CYTOCHROME C OXIDASE ACTIVITY	CYTOCHROME C OXIDASE ACTIVITY	Details ...	18	-0.6647342	-3.4626806	0.000	0.000	0.000	2556	tags=89%, list=23%, signal=115%
CYTOSOLIC RIBOSOME	CYTOSOLIC RIBOSOME	Details ...	74	-0.64356744	-6.4225993	0.000	0.000	0.000	1946	tags=81%, list=17%, signal=97%
LARGE RIBOSOMAL SUBUNIT	LARGE RIBOSOMAL SUBUNIT	Details ...	85	-0.56857693	-6.306569	0.000	0.000	0.000	1874	tags=73%, list=17%, signal=87%
MITOCHONDRIAL PROTON TRANSPORTING ATP SYNTHASE COMPLEX	MITOCHONDRIAL PROTON TRANSPORTING ATP SYNTHASE COMPLEX	Details ...	15	-0.67466897	-3.169331	0.000	0.000	0.000	1433	tags=80%, list=13%, signal=91%
MITOCHONDRIAL RESPIRATORY CHAIN COMPLEX I	MITOCHONDRIAL RESPIRATORY CHAIN COMPLEX I	Details ...	35	-0.5920298	-4.0417976	0.000	0.000	0.000	2057	tags=77%, list=18%, signal=94%
MITOCHONDRIAL_RESPIRATORY_CHAIN	MITOCHONDRIAL_RESPIRATORY_CHAIN	Details ...	59	-0.5750217	-5.16118	0.000	0.000	0.000	3318	tags=86%, list=29%, signal=122%
MITOCHONDRIAL RIBOSOME	MITOCHONDRIAL RIBOSOME	Details ...	80	-0.45795047	-4.818244	0.000	0.000	0.000	4060	tags=81%, list=36%, signal=126%
MITOCHONDRION	MITOCHONDRION	Details ...	1323	-0.13719822	-5.364362	0.000	0.000	0.000	3814	tags=46%, list=34%, signal=61%
PROTEASOME CORE COMPLEX ALPHA SUBUNIT COMPLEX	PROTEASOME CORE COMPLEX ALPHA SUBUNIT COMPLEX	Details ...	7	-0.79408056	-2.5507689	0.000	0.000	0.000	722	tags=86%, list=6%, signal=91%
PROTEASOME CORE COMPLEX	PROTEASOME CORE COMPLEX	Details ...	17	-0.66492707	-3.30452	0.000	0.000	0.000	2479	tags=88%, list=22%, signal=113%
RIBOSOME	RIBOSOME	Details ...	176	-0.46945027	-7.1704936	0.000	0.000	0.000	3202	tags=74%, list=28%, signal=102%
SMALL RIBOSOMAL SUBUNIT	SMALL RIBOSOMAL SUBUNIT	Details ...	61	-0.5441493	-5.056789	0.000	0.000	0.000	3160	tags=82%, list=28%, signal=113%
TRANSLATION	TRANSLATION	Details ...	437	-0.14763926	-3.5463178	0.000	0.000	0.000	1428	tags=27%, list=13%, signal=29%

Humanised mutant FUS drives progressive motor neuron degeneration without aggregation in 'FUSDelta14' knockin mice
Devoy et al. 2017
Supplementary Table 5 Gene Set Enrichment Analysis (GSEA) **Ribosome** - list of genes dysregulated at 12 months of age in FUS Delta14 spinal cord.

NAME	PROBE	GENE SYMBOL	GENE_TITLE	RANK IN GENE LIST	RANK METRIC SCORE	RUNNING ES	CORE ENRICHMENT
row_0	Abcf1	null	null	1251	24.42557335	-0.10632463	No
row_1	Mrps2	null	null	1259	24.30971718	-0.10126955	No
row_2	Rrbp1	null	null	1568	18.44819832	-0.12316406	No
row_3	Dhx9	null	null	1599	17.95916367	-0.12016825	No
row_4	Rps6kl1	null	null	1888	14.37590027	-0.14027208	No
row_5	Nsun4	null	null	2063	12.55372238	-0.1501691	No
row_6	Gemin5	null	null	2095	12.22449017	-0.14726283	No
row_7	Eif2ak4	null	null	2209	11.23381615	-0.15169829	No
row_8	Larp4b	null	null	2242	10.85581589	-0.14888154	No
row_9	Mrps5	null	null	3394	4.753861904	-0.24625282	No
row_10	Rps4l	null	null	3766	3.709308386	-0.27378795	No
row_11	Mrps27	null	null	3795	3.650242805	-0.27061307	No
row_12	Jrk	null	null	3799	3.641199589	-0.26519984	No
row_13	Surf6	null	null	4154	2.883627653	-0.2912129	No
row_14	Mrpl4	null	null	4233	2.762340069	-0.2925147	No
row_15	Hba-a1	null	null	4423	2.471960068	-0.30375472	No
row_16	Eif2d	null	null	4437	2.456758738	-0.29923683	No
row_17	Mrps18b	null	null	4504	2.353528023	-0.29946423	No
row_18	Mrpl38	null	null	4526	2.314575672	-0.2956626	No
row_19	Hba-a2	null	null	5330	1.447466493	-0.36187622	No
row_20	Mrpl45	null	null	5758	1.177889824	-0.3944252	No
row_21	Dap3	null	null	5767	1.17144978	-0.38945967	No
row_22	Mrpl37	null	null	5908	1.105024099	-0.39631256	No
row_23	Nle1	null	null	6077	1.017181993	-0.40567237	No
row_24	Mtg2	null	null	6111	1.00466454	-0.40294516	No
row_25	Nufip1	null	null	6305	-1.082650304	-0.4145433	No
row_26	Mrpl44	null	null	6398	-1.143575311	-0.41709858	No
row_27	Mpv17l2	null	null	6543	-1.227466464	-0.42430958	No
row_28	Mrpl10	null	null	6750	-1.36965251	-0.43707168	No
row_29	Mrpl43	null	null	6913	-1.524495244	-0.4458943	No
row_30	Mrpl28	null	null	6929	-1.549821138	-0.44155547	No
row_31	Prmt3	null	null	7022	-1.639651895	-0.44411075	No
row_32	Eef2	null	null	7128	-1.775381327	-0.44782993	No
row_33	Canx	null	null	7232	-1.933994889	-0.4513701	No
row_34	Mrpl49	null	null	7286	-2.00241971	-0.45043352	No
row_35	Mrps30	null	null	7406	-2.212972641	-0.45540622	No
row_36	Ptcd3	null	null	7407	-2.213463545	-0.44972438	No
row_37	Mrpl2	null	null	7421	-2.225229502	-0.4452065	No
row_38	Mrps7	null	null	7426	-2.229056597	-0.4398828	No
row_39	Mrps18a	null	null	7513	-2.375406742	-0.44190088	No
row_40	Mrps12	null	null	7532	-2.407607555	-0.4378307	No
row_41	Mrpl3	null	null	7668	-2.72729826	-0.4442359	No
row_42	Mrpl19	null	null	7721	-2.858035564	-0.4432098	No
row_43	Gadd45gip1	null	null	7784	-2.999709368	-0.44307905	No
row_44	Mrpl9	null	null	8043	-3.780286789	-0.4604969	No
row_45	Ppargc1a	null	null	8144	-4.17288065	-0.46376842	Yes
row_46	Mrpl34	null	null	8181	-4.340406895	-0.46130982	Yes
row_47	Mrps9	null	null	8186	-4.361042976	-0.45598614	Yes
row_48	Mrps11	null	null	8273	-4.816332817	-0.4580042	Yes
row_49	Zfp622	null	null	8297	-4.947680473	-0.45438164	Yes
row_50	Mrpl16	null	null	8337	-5.149276733	-0.45219165	Yes
row_51	Mrps26	null	null	8364	-5.248119354	-0.4488377	Yes
row_52	2810006K23Rik	null	null	8469	-5.951928139	-0.45246738	Yes
row_53	Mrps25	null	null	8533	-6.375924587	-0.45242617	Yes
row_54	Mrps23	null	null	8588	-6.735428333	-0.45157915	Yes
row_55	Mrps35	null	null	8642	-7.18482542	-0.45064262	Yes
row_56	Mtg1	null	null	8715	-7.807396889	-0.45140722	Yes
row_57	Mrpl14	null	null	8821	-8.893301964	-0.4551264	Yes
row_58	Tma16	null	null	8859	-9.341950417	-0.45275733	Yes
row_59	Nufip2	null	null	8943	-10.41436386	-0.4545068	Yes
row_60	Mrps17	null	null	9010	-11.25024986	-0.4547342	Yes

row_61	Larp4	null	null	9087	-12.32528591	-0.45585692	Yes
row_62	Hspa14	null	null	9096	-12.52689171	-0.45089138	Yes
row_63	Nck1	null	null	9108	-12.64617157	-0.44619444	Yes
row_64	Rpl7l1	null	null	9229	-14.61259651	-0.45125663	Yes
row_65	Mcts2	null	null	9400	-18.27958298	-0.46079552	Yes
row_66	Apod	null	null	9448	-19.3204422	-0.45932177	Yes
row_67	Rplp0	null	null	9472	-19.87993813	-0.45569924	Yes
row_68	Mrpl57	null	null	9499	-20.60731506	-0.45234528	Yes
row_69	Mrpl55	null	null	9547	-22.21569252	-0.45087153	Yes
row_70	Rpl27	null	null	9637	-25.04844666	-0.4531582	Yes
row_71	Mrps16	null	null	9645	-25.3657341	-0.44810313	Yes
row_72	Nsun3	null	null	9696	-27.04464722	-0.44689798	Yes
row_73	Rps11	null	null	9700	-27.1452446	-0.44148475	Yes
row_74	Zcchc17	null	null	9710	-27.51429367	-0.43660873	Yes
row_75	Mrpl39	null	null	9766	-29.67875099	-0.43585128	Yes
row_76	Fau	null	null	9869	-34.39938354	-0.43930188	Yes
row_77	Rps6	null	null	9871	-34.48515701	-0.4337096	Yes
row_78	Rpl8	null	null	9877	-34.74137497	-0.42847544	Yes
row_79	Mrpl17	null	null	9948	-38.69493866	-0.42906097	Yes
row_80	Pnpt1	null	null	9957	-39.1652565	-0.42409542	Yes
row_81	Mrpl46	null	null	9972	-40.10016251	-0.41966707	Yes
row_82	Rpl4	null	null	10000	-41.64800644	-0.41640264	Yes
row_83	Mrpl23	null	null	10021	-43.24662781	-0.4125115	Yes
row_84	Rps10	null	null	10036	-44.91659164	-0.40808314	Yes
row_85	Rps9	null	null	10042	-45.18154907	-0.40284902	Yes
row_86	Snca	null	null	10093	-48.96699905	-0.40164387	Yes
row_87	Rps15a	null	null	10155	-53.77290344	-0.4014236	Yes
row_88	Mrpl15	null	null	10165	-54.14429855	-0.3965476	Yes
row_89	Mrpl40	null	null	10166	-54.17938995	-0.39086574	Yes
row_90	Mrps28	null	null	10168	-54.25280762	-0.38527346	Yes
row_91	Mterf4	null	null	10187	-56.06287766	-0.38120326	Yes
row_92	Mrpl21	null	null	10188	-56.15470123	-0.37552145	Yes
row_93	Elavl4	null	null	10207	-57.84687424	-0.37145123	Yes
row_94	Rpl36	null	null	10253	-63.1675148	-0.36979842	Yes
row_95	Rpl28	null	null	10265	-64.4839859	-0.36510146	Yes
row_96	Rpl22	null	null	10300	-69.77206421	-0.36246377	Yes
row_97	Rps13	null	null	10334	-73.37827301	-0.35973656	Yes
row_98	Aurkaip1	null	null	10335	-73.55129242	-0.35405475	Yes
row_99	Rpl18	null	null	10353	-75.43097687	-0.349895	Yes
row_100	Mrpl20	null	null	10377	-78.18450928	-0.34627247	Yes
row_101	Rpl35a	null	null	10381	-78.56951141	-0.34085923	Yes
row_102	Rpl10a	null	null	10385	-78.81176758	-0.33544603	Yes
row_103	Chchd1	null	null	10430	-85.17641449	-0.33370367	Yes
row_104	Rpl14	null	null	10434	-85.91460419	-0.32829046	Yes
row_105	Rps25	null	null	10446	-88.5580368	-0.32359353	Yes
row_106	Mrpl32	null	null	10459	-91.17756653	-0.3189861	Yes
row_107	Mrpl11	null	null	10461	-91.59705353	-0.3133938	Yes
row_108	Mrpl35	null	null	10471	-93.95879364	-0.30851778	Yes
row_109	Rps17	null	null	10484	-95.03837585	-0.30391037	Yes
row_110	Mrps31	null	null	10487	-95.94171143	-0.29840764	Yes
row_111	Mrpl50	null	null	10512	-99.57808685	-0.2948746	Yes
row_112	Rpl17	null	null	10527	-101.6408234	-0.29044628	Yes
row_113	Mrps15	null	null	10533	-102.5806046	-0.28521213	Yes
row_114	Mrpl41	null	null	10549	-105.0257263	-0.2808733	Yes
row_115	Mrps33	null	null	10560	-106.3042831	-0.2760868	Yes
row_116	Rpl15	null	null	10565	-107.3610916	-0.27076313	Yes
row_117	Mrps36	null	null	10582	-113.0261688	-0.26651385	Yes
row_118	Rpl9	null	null	10583	-113.0406189	-0.26083204	Yes
row_119	Rps27l	null	null	10585	-114.0915375	-0.25523975	Yes
row_120	Rpl37a	null	null	10595	-115.9348679	-0.25036374	Yes
row_121	Mrpl27	null	null	10604	-117.6448135	-0.24539818	Yes
row_122	Mrpl12	null	null	10623	-122.7796249	-0.24132797	Yes
row_123	Rps27a	null	null	10632	-126.1458435	-0.23636241	Yes
row_124	Rps16	null	null	10643	-128.8344727	-0.23157594	Yes
row_125	Rps7	null	null	10653	-130.6835632	-0.22669992	Yes
row_126	Eif2a	null	null	10657	-131.8905945	-0.2212867	Yes

row_127	Mrps10	null	null	10666	-133.4852448	-0.21632116	Yes
row_128	Eif2s1	null	null	10684	-137.2604523	-0.2121614	Yes
row_129	Mrps14	null	null	10694	-141.0014954	-0.20728539	Yes
row_130	Npm1	null	null	10698	-142.3594055	-0.20187217	Yes
row_131	Rpl38	null	null	10710	-146.3634949	-0.19717522	Yes
row_132	Rpl34	null	null	10718	-148.569458	-0.19212013	Yes
row_133	Rps12	null	null	10721	-150.4437408	-0.18661739	Yes
row_134	Rps24	null	null	10724	-151.2233276	-0.18111463	Yes
row_135	Rpl26	null	null	10725	-151.5032654	-0.17543282	Yes
row_136	Rpl35	null	null	10727	-151.7383575	-0.16984053	Yes
row_137	Rpl23	null	null	10762	-162.8869324	-0.16720285	Yes
row_138	Rplp1	null	null	10771	-166.6326599	-0.1622373	Yes
row_139	Mrpl24	null	null	10786	-171.3456726	-0.15780894	Yes
row_140	Mrpl18	null	null	10794	-175.7474976	-0.15275386	Yes
row_141	Rps15	null	null	10803	-178.0572357	-0.14778832	Yes
row_142	Rpl11	null	null	10807	-179.357605	-0.1423751	Yes
row_143	Rpl29	null	null	10819	-183.9489899	-0.13767815	Yes
row_144	Rpl37	null	null	10851	-206.2558441	-0.13477187	Yes
row_145	Rps21	null	null	10854	-207.5920258	-0.12926912	Yes
row_146	Rps23	null	null	10860	-209.1409454	-0.12403497	Yes
row_147	Rps28	null	null	10871	-214.1931915	-0.11924849	Yes
row_148	Mrpl54	null	null	10882	-220.9447937	-0.114462	Yes
row_149	Rpl18a	null	null	10891	-225.9889374	-0.10949645	Yes
row_150	Mrpl47	null	null	10898	-228.3879852	-0.10435183	Yes
row_151	Rps18	null	null	10906	-235.6907349	-0.09929675	Yes
row_152	Mrps21	null	null	10920	-245.4321136	-0.09477887	Yes
row_153	Rpsa	null	null	10945	-261.1583557	-0.09124585	Yes
row_154	Rpl7	null	null	10948	-263.0768433	-0.08574311	Yes
row_155	Rps14	null	null	10969	-279.6862183	-0.08185196	Yes
row_156	Rps5	null	null	10993	-295.9665527	-0.07822941	Yes
row_157	Rps3a1	null	null	11014	-318.17099	-0.074338265	Yes
row_158	Mrpl42	null	null	11017	-321.1486816	-0.06883551	Yes
row_159	Rps19	null	null	11024	-326.9820251	-0.06369089	Yes
row_160	Mrpl22	null	null	11042	-341.1478577	-0.059531145	Yes
row_161	Ndufa7	null	null	11061	-366.0018311	-0.05546093	Yes
row_162	Rpl31	null	null	11082	-382.4345703	-0.051569782	Yes
row_163	Rps27	null	null	11100	-405.5938416	-0.047410034	Yes
row_164	mt-Rnr2	null	null	11107	-413.6788635	-0.04226542	Yes
row_165	Rps26	null	null	11142	-469.5862122	-0.03962774	Yes
row_166	Mrpl30	null	null	11147	-485.053894	-0.034304056	Yes
row_167	Rpl22l1	null	null	11148	-487.1173401	-0.028622236	Yes
row_168	mt-Rnr1	null	null	11155	-509.6646729	-0.02347762	Yes
row_169	Mrpl13	null	null	11158	-511.0132446	-0.017974868	Yes
row_170	Mrpl52	null	null	11173	-546.9841919	-0.01354652	Yes
row_171	Rps29	null	null	11182	-583.5073853	-0.00858097	Yes
row_172	Mrpl48	null	null	11190	-613.0897827	-0.003525886	Yes
row_173	Mrpl36	null	null	11231	-811.0979004	-0.001425409	Yes
row_174	Mrpl1	null	null	11233	-831.3317261	0.004166876	Yes
row_175	Mrps22	null	null	11326	-6304.582031	0.00161161	Yes

Humanised mutant FUS drives progressive motor neuron degeneration without aggregation in 'FUSDelta14' knockin mice
Devoy et al. 2017
Supplementary Table 6 Gene Set Enrichment Analysis (GSEA) **Large** r**ibosomal subunit** - list of genes dysregulated at 12 months of age in FUS Delta14 sp

NAME	PROBE	GENE SYMBOL	GENE_TITLE	RANK IN GENE LIST	RANK METRIC SCORE	RUNNING ES	CORE ENRICHMENT
row_0	Nsun4	null	null	2063	12.55372238	-0.17145021	No
row_1	Gemin5	null	null	2095	12.22449017	-0.16243862	No
row_2	Surf6	null	null	4154	2.883627653	-0.33344477	No
row_3	Mrpl4	null	null	4233	2.762340069	-0.32860723	No
row_4	Mrpl38	null	null	4526	2.314575672	-0.34277505	No
row_5	Mrpl45	null	null	5758	1.177889824	-0.4403354	No
row_6	Mrpl37	null	null	5908	1.105024099	-0.44180337	No
row_7	Mrpl44	null	null	6398	-1.143575311	-0.47346672	No
row_8	Mpv17l2	null	null	6543	-1.227466464	-0.47449064	No
row_9	Mrpl10	null	null	6750	-1.36965251	-0.48102078	No
row_10	Mrpl43	null	null	6913	-1.524495244	-0.4836433	No
row_11	Mrpl28	null	null	6929	-1.549821138	-0.47321072	No
row_12	Mrpl49	null	null	7286	-2.00241971	-0.49306238	No
row_13	Mrpl2	null	null	7421	-2.225229502	-0.4931982	No
row_14	Mrpl3	null	null	7668	-2.72729826	-0.50328076	No
row_15	Mrpl19	null	null	7721	-2.858035564	-0.49613413	No
row_16	Mrpl9	null	null	8043	-3.780286789	-0.5128774	No
row_17	Mrpl34	null	null	8181	-4.340406895	-0.5132797	No
row_18	Zfp622	null	null	8297	-4.947680473	-0.5117281	No
row_19	Mrpl16	null	null	8337	-5.149276733	-0.503427	No
row_20	2810006K23Rik	null	null	8469	-5.951928139	-0.5032964	No
row_21	Mrpl14	null	null	8821	-8.893301964	-0.522704	No
row_22	Rpl7l1	null	null	9229	-14.61259651	-0.5470849	No
row_23	Rplp0	null	null	9472	-19.87993813	-0.5568122	Yes
row_24	Mrpl55	null	null	9547	-22.21569252	-0.55161947	Yes
row_25	Rpl27	null	null	9637	-25.04844666	-0.5477588	Yes
row_26	Nsun3	null	null	9696	-27.04464722	-0.5411451	Yes
row_27	Zcchc17	null	null	9710	-27.51429367	-0.5305349	Yes
row_28	Mrpl39	null	null	9766	-29.67875099	-0.52365476	Yes
row_29	Rpl8	null	null	9877	-34.74137497	-0.52165914	Yes
row_30	Mrpl17	null	null	9948	-38.69493866	-0.51611114	Yes
row_31	Mrpl46	null	null	9972	-40.10016251	-0.5063891	Yes
row_32	Rpl4	null	null	10000	-41.64800644	-0.49702224	Yes
row_33	Mrpl23	null	null	10021	-43.24662781	-0.48703372	Yes
row_34	Mrpl15	null	null	10165	-54.14429855	-0.48796883	Yes
row_35	Mrpl40	null	null	10166	-54.17938995	-0.47620413	Yes
row_36	Mterf4	null	null	10187	-56.06287766	-0.46621564	Yes
row_37	Mrpl21	null	null	10188	-56.15470123	-0.45445094	Yes
row_38	Rpl36	null	null	10253	-63.1675148	-0.44837007	Yes
row_39	Rpl28	null	null	10265	-64.4839859	-0.43758225	Yes
row_40	Rpl22	null	null	10300	-69.77206421	-0.4288371	Yes
row_41	Rpl18	null	null	10353	-75.43097687	-0.4216905	Yes
row_42	Mrpl20	null	null	10377	-78.18450928	-0.41196844	Yes
row_43	Rpl35a	null	null	10381	-78.56951141	-0.40047014	Yes
row_44	Rpl10a	null	null	10385	-78.81176758	-0.38897187	Yes
row_45	Rpl14	null	null	10434	-85.91460419	-0.38147005	Yes
row_46	Mrpl32	null	null	10459	-91.17756653	-0.37183678	Yes
row_47	Mrpl11	null	null	10461	-91.59705353	-0.3601609	Yes
row_48	Mrpl35	null	null	10471	-93.95879364	-0.34919545	Yes
row_49	Mrpl50	null	null	10512	-99.57808685	-0.34098315	Yes
row_50	Rpl17	null	null	10527	-101.6408234	-0.3304618	Yes
row_51	Mrpl41	null	null	10549	-105.0257263	-0.3205621	Yes
row_52	Rpl15	null	null	10565	-107.3610916	-0.31012952	Yes
row_53	Rpl9	null	null	10583	-113.0406189	-0.2998746	Yes
row_54	Rpl37a	null	null	10595	-115.9348679	-0.2890868	Yes
row_55	Mrpl27	null	null	10604	-117.6448135	-0.27803257	Yes
row_56	Mrpl12	null	null	10623	-122.7796249	-0.26786646	Yes
row_57	Npm1	null	null	10698	-142.3594055	-0.26267368	Yes
row_58	Rpl38	null	null	10710	-146.3634949	-0.2518859	Yes
row_59	Rpl34	null	null	10718	-148.569458	-0.24074285	Yes

row_60	Rps12	null	null	10721	-150.4437408	-0.22915576	Yes
row_61	Rpl26	null	null	10725	-151.5032654	-0.21765748	Yes
row_62	Rpl35	null	null	10727	-151.7383575	-0.20598158	Yes
row_63	Rpl23	null	null	10762	-162.8869324	-0.19723642	Yes
row_64	Rplp1	null	null	10771	-166.6326599	-0.18618219	Yes
row_65	Mrpl24	null	null	10786	-171.3456726	-0.17566082	Yes
row_66	Mrpl18	null	null	10794	-175.7474976	-0.16451779	Yes
row_67	Rpl11	null	null	10807	-179.357605	-0.1538188	Yes
row_68	Rpl29	null	null	10819	-183.9489899	-0.143031	Yes
row_69	Rpl37	null	null	10851	-206.2558441	-0.1340194	Yes
row_70	Mrpl54	null	null	10882	-220.9447937	-0.124919	Yes
row_71	Rpl18a	null	null	10891	-225.9889374	-0.11386477	Yes
row_72	Mrpl47	null	null	10898	-228.3879852	-0.102632925	Yes
row_73	Rpl7	null	null	10948	-263.0768433	-0.09521991	Yes
row_74	Mrpl42	null	null	11017	-321.1486816	-0.08949428	Yes
row_75	Mrpl22	null	null	11042	-341.1478577	-0.07986101	Yes
row_76	Rpl31	null	null	11082	-382.4345703	-0.07155989	Yes
row_77	mt-Rnr2	null	null	11107	-413.6788635	-0.061926626	Yes
row_78	Mrpl30	null	null	11147	-485.053894	-0.053625505	Yes
row_79	Rpl22l1	null	null	11148	-487.1173401	-0.0418608	Yes
row_80	Mrpl13	null	null	11158	-511.0132446	-0.030895384	Yes
row_81	Mrpl52	null	null	11173	-546.9841919	-0.020374017	Yes
row_82	Mrpl48	null	null	11190	-613.0897827	-0.01003027	Yes
row_83	Mrpl36	null	null	11231	-811.0979004	-0.001817962	Yes
row_84	Mrpl1	null	null	11233	-831.3317261	0.009857935	Yes

inal cord.

Humanised mutant FUS drives progressive motor neuron degeneration without aggregation in 'FUSDelta14' knockin mice
Devoy et al. 2017
Supplementary Table 7 Gene Set Enrichment Analysis (GSEA) **Mitochondrion** - list of genes dysregulated at 12 months of age in FUS Delta14 spinal cord.

NAME	PROBE	GENE SYMBOL	GENE_TITLE	RANK IN GENE LIST	RANK METRIC SCORE	RUNNING ES	CORE ENRICHMENT
row_0	Tgm2	null	null	1	30555.05859	6.56E-04	No
row_1	Gramd4	null	null	27	885.7765503	-0.001082577	No
row_2	Smurf1	null	null	31	848.8059692	-6.26E-04	No
row_3	Me2	null	null	33	714.2062988	3.00E-05	No
row_4	Aldh18a1	null	null	73	351.8300171	-0.003105564	No
row_5	Tnfrsf1a	null	null	75	341.1329956	-0.002449486	No
row_6	Ilf3	null	null	129	200.5873413	-0.006981994	No
row_7	Ptcd1	null	null	142	188.5109711	-0.007423502	No
row_8	Slc8a2	null	null	156	176.8764343	-0.00796479	No
row_9	Tap1	null	null	211	138.2415009	-0.012597078	No
row_10	Acox3	null	null	268	114.7536926	-0.017428927	No
row_11	Adsl	null	null	289	109.3579407	-0.01866868	No
row_12	Prkca	null	null	332	94.74576569	-0.022103602	No
row_13	H6pd	null	null	340	92.17034149	-0.022046207	No
row_14	Acaca	null	null	346	90.62123108	-0.02178925	No
row_15	Pla2g15	null	null	380	81.20423889	-0.02432615	No
row_16	Srgap2	null	null	408	75.19145966	-0.026264364	No
row_17	Ambra1	null	null	416	74.48309326	-0.02620697	No
row_18	Mavs	null	null	434	71.58919525	-0.02714738	No
row_19	Slc11a2	null	null	474	65.73648071	-0.030282961	No
row_20	Pnkd	null	null	476	65.36031342	-0.029626884	No
row_21	Hap1	null	null	495	61.59661865	-0.030667074	No
row_22	Ago2	null	null	506	60.78369904	-0.03090902	No
row_23	Atad3a	null	null	523	59.092659	-0.03174965	No
row_24	Cds2	null	null	534	57.90937805	-0.031991597	No
row_25	Prkcd	null	null	567	54.89591217	-0.034428716	No
row_26	Lrrk1	null	null	577	53.73189545	-0.03457088	No
row_27	Akap1	null	null	609	50.97730255	-0.03690822	No
row_28	Slc8b1	null	null	614	50.38896561	-0.036551483	No
row_29	Kcnma1	null	null	620	50.07435226	-0.036294527	No
row_30	Nptx1	null	null	636	48.59153748	-0.037035376	No
row_31	Tchp	null	null	663	46.55474854	-0.03887381	No
row_32	Gsk3b	null	null	674	45.43483734	-0.039115757	No
row_33	Acox1	null	null	675	45.42578506	-0.0383599	No
row_34	Bcl2l13	null	null	689	44.94870758	-0.038901187	No
row_35	Tnrc18	null	null	712	43.39552307	-0.0403405	No
row_36	Fndc1	null	null	734	42.10077286	-0.041680034	No
row_37	Dnm1	null	null	737	41.86163712	-0.041123737	No
row_38	Vat1	null	null	746	41.18077087	-0.041166123	No
row_39	Srebf2	null	null	748	41.1349144	-0.040510044	No
row_40	1110008L16Rik	null	null	767	40.52392197	-0.041550238	No
row_41	Tmem8b	null	null	769	40.41680145	-0.04089416	No
row_42	Afg3l2	null	null	776	39.98567581	-0.040736984	No
row_43	Fasn	null	null	781	39.38042831	-0.040380247	No
row_44	Psen2	null	null	791	38.98199844	-0.040522415	No
row_45	Src	null	null	792	38.97668076	-0.039766558	No
row_46	Zmiz2	null	null	812	37.18345261	-0.040906526	No
row_47	Ctsa	null	null	845	35.89429855	-0.043343645	No
row_48	Wasf1	null	null	868	34.91271973	-0.04478296	No
row_49	Mthfd1	null	null	878	34.52032471	-0.044925123	No
row_50	Ogdh	null	null	882	34.38605499	-0.044468608	No
row_51	Vps13c	null	null	883	34.32542801	-0.04371275	No
row_52	Dhx29	null	null	885	34.30755234	-0.04305667	No
row_53	Herc2	null	null	911	33.40600586	-0.044795327	No
row_54	Mmp2	null	null	912	33.39179993	-0.04403947	No
row_55	Aco1	null	null	917	33.28639221	-0.04368273	No
row_56	Nol6	null	null	919	33.26008987	-0.043026656	No
row_57	Hk1	null	null	950	31.99718094	-0.04526421	No
row_58	Zdhhc8	null	null	961	31.75670624	-0.045506157	No
row_59	Nt5dc3	null	null	977	31.27624893	-0.04624701	No
row_60	Mars	null	null	982	30.92882156	-0.04589027	No
row_61	Abat	null	null	986	30.82634735	-0.045433756	No

row_62	Slit3	null	null	1001	30.14528084	-0.046074823	No
row_63	Nos1	null	null	1026	29.58099365	-0.047713697	No
row_64	Lrp5	null	null	1059	28.51641846	-0.050150815	No
row_65	Bri3bp	null	null	1095	27.79366302	-0.052887276	No
row_66	Mapk10	null	null	1097	27.68331718	-0.052231196	No
row_67	Acacb	null	null	1122	27.01604843	-0.05387007	No
row_68	Eln	null	null	1133	26.81711578	-0.054112017	No
row_69	Snd1	null	null	1142	26.66940117	-0.054154404	No
row_70	Nfkb1	null	null	1160	26.14028549	-0.055094812	No
row_71	Sars2	null	null	1192	25.4682312	-0.057432152	No
row_72	Atg13	null	null	1202	25.24794197	-0.057574317	No
row_73	Sardh	null	null	1210	25.12393951	-0.05751692	No
row_74	Trak1	null	null	1228	24.87200928	-0.058457334	No
row_75	Raf1	null	null	1237	24.67622185	-0.05849972	No
row_76	Heatr1	null	null	1241	24.64696312	-0.0580432	No
row_77	Lpin1	null	null	1254	24.37413216	-0.05848471	No
row_78	Mfn2	null	null	1257	24.32611847	-0.057928413	No
row_79	Mrps2	null	null	1259	24.30971718	-0.057272337	No
row_80	Hsdl1	null	null	1263	24.21035767	-0.056815818	No
row_81	Hjurp	null	null	1277	23.73453903	-0.057357106	No
row_82	Rai1	null	null	1286	23.56643867	-0.057399493	No
row_83	Camk2a	null	null	1291	23.41599274	-0.05704276	No
row_84	Hivep1	null	null	1292	23.38706017	-0.0562869	No
row_85	Arsb	null	null	1308	23.10136414	-0.05702775	No
row_86	Tars2	null	null	1318	22.91293907	-0.057169914	No
row_87	Atcay	null	null	1360	22.24528694	-0.06050506	No
row_88	Sirt5	null	null	1364	22.21564484	-0.06004854	No
row_89	Gtf3c4	null	null	1379	21.81399536	-0.06068961	No
row_90	Elac2	null	null	1405	21.40831947	-0.062428262	No
row_91	Grn	null	null	1409	21.33284378	-0.061971746	No
row_92	Stxbp1	null	null	1418	21.12141609	-0.062014133	No
row_93	Gbf1	null	null	1430	20.78752518	-0.06235586	No
row_94	Synj2	null	null	1456	20.35732269	-0.06409451	No
row_95	Nos3	null	null	1460	20.31061554	-0.063638	No
row_96	Alas1	null	null	1462	20.29590797	-0.06298192	No
row_97	Mtor	null	null	1474	20.091465	-0.06332365	No
row_98	Lig1	null	null	1481	19.99988365	-0.06316648	No
row_99	Dnm2	null	null	1488	19.91637421	-0.0630093	No
row_100	Cpt1a	null	null	1491	19.80084038	-0.062453	No
row_101	Slc25a35	null	null	1496	19.76806641	-0.062096264	No
row_102	Slc8a3	null	null	1501	19.70778465	-0.06173953	No
row_103	Chpf	null	null	1527	19.11482048	-0.06347819	No
row_104	Abcc8	null	null	1541	18.91813087	-0.06401947	No
row_105	Eefsec	null	null	1543	18.88107872	-0.063363396	No
row_106	Pcx	null	null	1550	18.76885033	-0.06320622	No
row_107	Slc25a22	null	null	1619	17.6996994	-0.06923544	No
row_108	Stat3	null	null	1629	17.52118874	-0.0693776	No
row_109	Por	null	null	1656	17.13784981	-0.07121603	No
row_110	Pop1	null	null	1659	17.11472893	-0.07065974	No
row_111	Prkce	null	null	1732	16.1432209	-0.07708807	No
row_112	Hlcs	null	null	1745	15.97098351	-0.07752958	No
row_113	Cerk	null	null	1757	15.80545139	-0.07787131	No
row_114	Aars2	null	null	1758	15.80330276	-0.077115454	No
row_115	Tomm34	null	null	1760	15.7971344	-0.07645938	No
row_116	Jarid2	null	null	1767	15.71577835	-0.0763022	No
row_117	Spns1	null	null	1777	15.60892487	-0.076444365	No
row_118	Letm1	null	null	1778	15.59956074	-0.07568851	No
row_119	Abcg1	null	null	1788	15.46698666	-0.075830676	No
row_120	Atic	null	null	1795	15.38154316	-0.0756735	No
row_121	Dhx32	null	null	1801	15.32862186	-0.07541654	No
row_122	Thop1	null	null	1805	15.26246834	-0.07496003	No
row_123	Kank2	null	null	1815	15.14765263	-0.075102195	No
row_124	Nnt	null	null	1818	15.13646603	-0.0745459	No
row_125	Sox10	null	null	1825	15.05711555	-0.07438872	No
row_126	Kcnj11	null	null	1827	15.03812504	-0.073732644	No
row_127	Vwa8	null	null	1830	14.9704361	-0.07317635	No
row_128	Gtpbp3	null	null	1871	14.61792278	-0.07641171	No

row_129	Hs1bp3	null	null	1875	14.53185177	-0.07595519	No
row_130	Dcaf5	null	null	1905	14.16957188	-0.07809297	No
row_131	Mrm1	null	null	1910	14.08028889	-0.077736236	No
row_132	Trub1	null	null	1946	13.7241745	-0.08047269	No
row_133	Fyn	null	null	1956	13.64822292	-0.08061486	No
row_134	Nacc2	null	null	1971	13.5198288	-0.08125593	No
row_135	Txnrd1	null	null	1987	13.36809254	-0.081996776	No
row_136	Pdpr	null	null	2000	13.24549294	-0.08243828	No
row_137	Pacs2	null	null	2001	13.2157011	-0.08168243	No
row_138	Psen1	null	null	2036	12.87554932	-0.08431911	No
row_139	Gad1	null	null	2048	12.7449131	-0.084660836	No
row_140	Nsun4	null	null	2063	12.55372238	-0.0853019	No
row_141	Coq8a	null	null	2078	12.3939724	-0.08594297	No
row_142	Pitrm1	null	null	2099	12.19889641	-0.08718272	No
row_143	Pdk2	null	null	2107	12.11560631	-0.08712532	No
row_144	Nlrx1	null	null	2108	12.10990238	-0.08636947	No
row_145	Nat8l	null	null	2140	11.88866329	-0.08870681	No
row_146	Cat	null	null	2156	11.77019882	-0.089447655	No
row_147	Mlxip	null	null	2159	11.76479816	-0.08889136	No
row_148	Abcf2	null	null	2165	11.71659088	-0.0886344	No
row_149	Bcl2l11	null	null	2175	11.55470085	-0.08877657	No
row_150	Efhd1	null	null	2177	11.52617168	-0.08812049	No
row_151	Pdp2	null	null	2189	11.43609905	-0.08846222	No
row_152	Dnajc5	null	null	2207	11.24533939	-0.08940263	No
row_153	Cry2	null	null	2211	11.20897484	-0.08894611	No
row_154	Acsl1	null	null	2212	11.19427395	-0.08819026	No
row_155	Aldh2	null	null	2215	11.15061951	-0.08763396	No
row_156	Dnajc11	null	null	2261	10.69070911	-0.09136822	No
row_157	Spg7	null	null	2273	10.5933857	-0.09170995	No
row_158	Snph	null	null	2280	10.55253887	-0.09155277	No
row_159	Slc25a25	null	null	2288	10.46755219	-0.09149538	No
row_160	Tigar	null	null	2302	10.32269001	-0.09203667	No
row_161	Fahd2a	null	null	2310	10.2719183	-0.09197927	No
row_162	Prkaca	null	null	2312	10.25896931	-0.0913232	No
row_163	Ivd	null	null	2322	10.19625473	-0.09146536	No
row_164	Ide	null	null	2328	10.12988567	-0.091208406	No
row_165	Ass1	null	null	2329	10.12852764	-0.09045255	No
row_166	Lrrk2	null	null	2339	10.0526638	-0.09059472	No
row_167	Ccar2	null	null	2340	10.04743385	-0.089838855	No
row_168	Sptlc2	null	null	2348	9.992437363	-0.08978146	No
row_169	Pi4k2a	null	null	2350	9.987475395	-0.08912539	No
row_170	Dcakd	null	null	2355	9.964668274	-0.08876865	No
row_171	Vars	null	null	2358	9.947878838	-0.088212356	No
row_172	Psap	null	null	2359	9.945831299	-0.087456495	No
row_173	Kif1b	null	null	2383	9.778719902	-0.08899559	No
row_174	Acad10	null	null	2384	9.778583527	-0.08823973	No
row_175	Ddah1	null	null	2410	9.573734283	-0.08997838	No
row_176	Elk3	null	null	2417	9.561273575	-0.08982121	No
row_177	Foxo3	null	null	2427	9.478625298	-0.08996338	No
row_178	Gpam	null	null	2452	9.280852318	-0.09160225	No
row_179	Mrs2	null	null	2476	9.17769146	-0.09314134	No
row_180	Mmab	null	null	2491	9.063270569	-0.09378241	No
row_181	Crat	null	null	2492	9.004062653	-0.093026556	No
row_182	Pmpca	null	null	2525	8.722874641	-0.09546367	No
row_183	Zfp13	null	null	2538	8.658646584	-0.09590518	No
row_184	Pi4kb	null	null	2574	8.449771881	-0.09864164	No
row_185	Ern1	null	null	2585	8.384327888	-0.098883584	No
row_186	Wdr81	null	null	2595	8.287446976	-0.09902575	No
row_187	Asah2	null	null	2610	8.202823639	-0.09966682	No
row_188	Shc1	null	null	2612	8.188264847	-0.09901074	No
row_189	Ldhd	null	null	2614	8.171419144	-0.09835467	No
row_190	Zfhx3	null	null	2615	8.164732933	-0.097598806	No
row_191	Slc25a10	null	null	2621	8.128980637	-0.09734185	No
row_192	Plscr3	null	null	2624	8.120078087	-0.09678555	No
row_193	Acly	null	null	2625	8.108164787	-0.0960297	No
row_194	Aldh7a1	null	null	2637	8.047185898	-0.09637143	No
row_195	Tcirg1	null	null	2644	8.004177094	-0.09621425	No

row_196	Phyhipl	null	null	2662	7.928709984	-0.09715466	No
row_197	Cs	null	null	2667	7.903690815	-0.09679792	No
row_198	Aldh9a1	null	null	2673	7.860301495	-0.096540965	No
row_199	Slc25a42	null	null	2679	7.821308613	-0.09628401	No
row_200	Hadha	null	null	2689	7.783488274	-0.09642618	No
row_201	Stard7	null	null	2697	7.743124962	-0.09636878	No
row_202	Spg20	null	null	2716	7.648876667	-0.09740897	No
row_203	Sncb	null	null	2741	7.55259943	-0.09904785	No
row_204	Trit1	null	null	2749	7.497159004	-0.098990455	No
row_205	Usp30	null	null	2757	7.435054302	-0.09893306	No
row_206	Mapk14	null	null	2786	7.293083191	-0.10097106	No
row_207	Agap2	null	null	2795	7.226827621	-0.101013444	No
row_208	Acsf3	null	null	2798	7.219959259	-0.10045715	No
row_209	Rnf144b	null	null	2824	7.089013577	-0.1021958	No
row_210	Myo19	null	null	2828	7.068799019	-0.10173928	No
row_211	Mto1	null	null	2844	6.97027874	-0.10248013	No
row_212	Tshz3	null	null	2849	6.945355892	-0.102123395	No
row_213	Atp10d	null	null	2857	6.916809559	-0.102066	No
row_214	Trap1	null	null	2858	6.909668446	-0.10131014	No
row_215	Lonp1	null	null	2865	6.871529102	-0.10115297	No
row_216	Agtpbp1	null	null	2903	6.70650053	-0.10408899	No
row_217	Sh3bp5	null	null	2911	6.670181751	-0.10403159	No
row_218	Th	null	null	2917	6.63672924	-0.10377464	No
row_219	Aifm2	null	null	2919	6.632403374	-0.10311856	No
row_220	Lrpprc	null	null	2921	6.619216919	-0.102462485	No
row_221	Mecr	null	null	2934	6.556781769	-0.10290399	No
row_222	Rpusd3	null	null	2935	6.556063652	-0.10214813	No
row_223	Epha4	null	null	2937	6.547751427	-0.101492055	No
row_224	Mthfd1l	null	null	2943	6.540843964	-0.1012351	No
row_225	Fen1	null	null	2960	6.49056673	-0.10207573	No
row_226	Xpc	null	null	2966	6.467611313	-0.10181878	No
row_227	Oxa1l	null	null	2971	6.455383778	-0.10146204	No
row_228	Xpnpep3	null	null	2975	6.428705692	-0.101005524	No
row_229	Mdh2	null	null	2980	6.417682648	-0.10064878	No
row_230	Tfap4	null	null	3017	6.232719898	-0.103485025	No
row_231	Brd8	null	null	3019	6.214444637	-0.10282895	No
row_232	Tmem143	null	null	3022	6.202355385	-0.10227265	No
row_233	Dnajc27	null	null	3026	6.194260597	-0.10181613	No
row_234	Fancg	null	null	3037	6.135654926	-0.10205808	No
row_235	Mcu	null	null	3042	6.115357399	-0.10170135	No
row_236	Abl1	null	null	3049	6.083391666	-0.10154417	No
row_237	Bphl	null	null	3055	6.061126709	-0.101287216	No
row_238	Slc25a44	null	null	3074	5.948134422	-0.10232741	No
row_239	Mtfmt	null	null	3078	5.936202526	-0.10187089	No
row_240	Gpd2	null	null	3101	5.844689846	-0.103310205	No
row_241	Slc25a23	null	null	3125	5.735219002	-0.104849294	No
row_242	Polg	null	null	3134	5.706501007	-0.10489168	No
row_243	Rab11b	null	null	3136	5.701321602	-0.104235604	No
row_244	Hsd17b4	null	null	3153	5.644258499	-0.10507623	No
row_245	Parp9	null	null	3162	5.612594604	-0.10511862	No
row_246	Cox15	null	null	3163	5.606339931	-0.10436276	No
row_247	Txnrd2	null	null	3175	5.567015171	-0.10470449	No
row_248	Dpysl2	null	null	3187	5.530966282	-0.10504622	No
row_249	Sfxn2	null	null	3189	5.515417099	-0.10439014	No
row_250	Aldh5a1	null	null	3196	5.481945992	-0.10423297	No
row_251	Tpp1	null	null	3204	5.461959839	-0.10417557	No
row_252	Ppif	null	null	3224	5.374773026	-0.10531554	No
row_253	Tdrd7	null	null	3228	5.361707687	-0.104859024	No
row_254	D2hgdh	null	null	3236	5.335103989	-0.10480163	No
row_255	Hmgcs2	null	null	3240	5.303886414	-0.10434511	No
row_256	Cry1	null	null	3252	5.268076897	-0.10468684	No
row_257	Trp53	null	null	3255	5.257921696	-0.104130544	No
row_258	Slc25a12	null	null	3264	5.231944561	-0.10417293	No
row_259	Stard3	null	null	3273	5.203613758	-0.10421532	No
row_260	Abcc9	null	null	3280	5.185572147	-0.10405814	No
row_261	Dhx57	null	null	3285	5.167887688	-0.103701405	No
row_262	Usp48	null	null	3289	5.144835949	-0.103244886	No

row_263	Gldc	null	null	3308	5.075715542	-0.104285076	No
row_264	Mxd1	null	null	3318	5.009304047	-0.10442725	No
row_265	Hsp90ab1	null	null	3365	4.84558773	-0.10826129	No
row_266	Abcd3	null	null	3379	4.801146507	-0.10880258	No
row_267	Iqce	null	null	3387	4.786997318	-0.10874518	No
row_268	Cpt1c	null	null	3388	4.775093079	-0.107989326	No
row_269	Mrps5	null	null	3394	4.753861904	-0.10773237	No
row_270	Serac1	null	null	3399	4.72367239	-0.10737564	No
row_271	Adh1	null	null	3410	4.709075928	-0.10761758	No
row_272	Msto1	null	null	3421	4.674862862	-0.10785953	No
row_273	Thra	null	null	3438	4.621332645	-0.108700156	No
row_274	Fads1	null	null	3442	4.606935978	-0.108243644	No
row_275	Afg3l1	null	null	3447	4.584078312	-0.1078869	No
row_276	Asl	null	null	3453	4.567703724	-0.10762995	No
row_277	Nipsnap1	null	null	3463	4.533967018	-0.10777212	No
row_278	Exog	null	null	3464	4.531827927	-0.10701626	No
row_279	Sarm1	null	null	3469	4.523144722	-0.106659524	No
row_280	Lig3	null	null	3474	4.497749329	-0.10630278	No
row_281	Dmpk	null	null	3475	4.496816635	-0.10554693	No
row_282	Chdh	null	null	3477	4.488336563	-0.10489085	No
row_283	Acsf2	null	null	3480	4.471518993	-0.104334556	No
row_284	Kif1bp	null	null	3490	4.449288845	-0.10447672	No
row_285	Ctsb	null	null	3507	4.424388885	-0.10531735	No
row_286	Nefh	null	null	3508	4.423994541	-0.10456149	No
row_287	Pde2a	null	null	3516	4.400578022	-0.1045041	No
row_288	Mief1	null	null	3519	4.39420414	-0.1039478	No
row_289	Slc27a1	null	null	3536	4.313569069	-0.10478843	No
row_290	Got2	null	null	3538	4.310852528	-0.104132354	No
row_291	Sirt4	null	null	3540	4.303637505	-0.10347628	No
row_292	0610009O20Rik	null	null	3542	4.301675797	-0.102820195	No
row_293	Polrmt	null	null	3544	4.299694538	-0.10216412	No
row_294	Mutyh	null	null	3555	4.252169609	-0.10240607	No
row_295	Bdh1	null	null	3567	4.221230984	-0.1027478	No
row_296	Clu	null	null	3578	4.201067924	-0.10298974	No
row_297	Atp5sl	null	null	3594	4.17263937	-0.10373059	No
row_298	Dcaf8	null	null	3595	4.170204639	-0.102974735	No
row_299	Secisbp2	null	null	3597	4.166490555	-0.10231866	No
row_300	Gabbr1	null	null	3629	4.085955143	-0.104655996	No
row_301	Bckdha	null	null	3632	4.071686268	-0.1040997	No
row_302	Aldh3a2	null	null	3636	4.063464642	-0.10364318	No
row_303	Hspa9	null	null	3644	4.034802914	-0.10358579	No
row_304	Rnasel	null	null	3645	4.025636673	-0.102829926	No
row_305	Sdha	null	null	3649	4.021390915	-0.102373414	No
row_306	Capn1	null	null	3660	4.000296593	-0.10261536	No
row_307	Zbed5	null	null	3675	3.958540201	-0.10325643	No
row_308	Ucp2	null	null	3681	3.944452286	-0.10299947	No
row_309	Slc25a38	null	null	3709	3.84490633	-0.10493769	No
row_310	Acsl5	null	null	3717	3.83062315	-0.10488029	No
row_311	Stard5	null	null	3724	3.811373234	-0.10472312	No
row_312	Sox4	null	null	3732	3.798035622	-0.10466572	No
row_313	9030617O03Rik	null	null	3733	3.792416573	-0.103909865	No
row_314	Pus1	null	null	3740	3.769305468	-0.10375269	No
row_315	Casp9	null	null	3777	3.683356524	-0.10658893	No
row_316	Aldh1l2	null	null	3779	3.678087473	-0.105932854	No
row_317	Samm50	null	null	3783	3.670893431	-0.105476335	No
row_318	Plcd1	null	null	3785	3.663417816	-0.10482026	No
row_319	Mccc1	null	null	3792	3.653384447	-0.10466308	No
row_320	Akt1	null	null	3794	3.652406454	-0.104007006	No
row_321	Mrps27	null	null	3795	3.650242805	-0.103251144	No
row_322	Htra2	null	null	3802	3.639060259	-0.103093974	No
row_323	Rai14	null	null	3804	3.634070873	-0.10243789	No
row_324	Mtus1	null	null	3814	3.616141319	-0.10258006	No
row_325	Dna2	null	null	3823	3.595425129	-0.10262245	No
row_326	Ptpn11	null	null	3825	3.592255116	-0.101966366	No
row_327	2310061I04Rik	null	null	3840	3.551478624	-0.10260744	No
row_328	Timm44	null	null	3845	3.543540001	-0.1022507	No
row_329	Acat2	null	null	3857	3.525716305	-0.10259243	No

row_330	Abcb8	null	null	3862	3.507148743	-0.1022357	No
row_331	Drg2	null	null	3870	3.497701883	-0.1021783	No
row_332	Letm2	null	null	3885	3.448040724	-0.10281937	No
row_333	Dao	null	null	3890	3.435639143	-0.102462634	No
row_334	Casp2	null	null	3891	3.430714369	-0.10170677	No
row_335	Vars2	null	null	3896	3.422293186	-0.10135004	No
row_336	Pkm	null	null	3917	3.371460199	-0.10258979	No
row_337	Ogdhl	null	null	3922	3.365113974	-0.10223305	No
row_338	Cltc	null	null	3924	3.358335495	-0.10157698	No
row_339	Akap8	null	null	3946	3.298427105	-0.10291651	No
row_340	Gls2	null	null	3951	3.282819033	-0.102559775	No
row_341	Aifm3	null	null	3970	3.250430822	-0.103599966	No
row_342	Nln	null	null	3983	3.23709631	-0.10404147	No
row_343	Gars	null	null	3994	3.220311165	-0.104283415	No
row_344	Dgat2	null	null	4002	3.205233097	-0.10422602	No
row_345	Mmachc	null	null	4003	3.204890728	-0.10347017	No
row_346	Atpaf1	null	null	4019	3.171010256	-0.10421102	No
row_347	Ankzf1	null	null	4022	3.164656639	-0.10365472	No
row_348	Acp6	null	null	4032	3.137387514	-0.103796884	No
row_349	Cox18	null	null	4046	3.105129004	-0.10433818	No
row_350	Dact2	null	null	4048	3.102981567	-0.10368209	No
row_351	Dlst	null	null	4061	3.065677166	-0.10412361	No
row_352	Rnaseh1	null	null	4095	3.002027512	-0.1066605	No
row_353	Shmt2	null	null	4096	3.001744986	-0.105904646	No
row_354	Ltbp1	null	null	4106	2.982506275	-0.10604681	No
row_355	Acss1	null	null	4119	2.948084354	-0.10648832	No
row_356	Sphkap	null	null	4130	2.923780918	-0.10673027	No
row_357	Clic4	null	null	4142	2.910177708	-0.107071996	No
row_358	Acot1	null	null	4171	2.850931406	-0.10910999	No
row_359	Supv3l1	null	null	4195	2.821311712	-0.11064908	No
row_360	Dnm3	null	null	4204	2.812295675	-0.110691465	No
row_361	Cyb5r3	null	null	4221	2.777993917	-0.1115321	No
row_362	Ywhaz	null	null	4225	2.771474361	-0.11107558	No
row_363	Mrpl4	null	null	4233	2.762340069	-0.11101819	No
row_364	Slc8a1	null	null	4246	2.743217707	-0.111459695	No
row_365	Gcdh	null	null	4256	2.729022741	-0.11160186	No
row_366	Cdk5rap1	null	null	4265	2.712977886	-0.111644246	No
row_367	Prosc	null	null	4288	2.686755419	-0.11308356	No
row_368	Txnrd3	null	null	4301	2.674651384	-0.11352507	No
row_369	Ndrg4	null	null	4323	2.644342422	-0.114864595	No
row_370	Ddit4	null	null	4324	2.642177343	-0.11410874	No
row_371	Ncbp1	null	null	4335	2.628850222	-0.11435069	No
row_372	Txnip	null	null	4337	2.623420954	-0.11369461	No
row_373	Wars2	null	null	4349	2.590276957	-0.11403634	No
row_374	Rab35	null	null	4352	2.582321644	-0.11348004	No
row_375	Pccb	null	null	4356	2.579746723	-0.11302353	No
row_376	Abca8a	null	null	4358	2.577151775	-0.112367444	No
row_377	Opa3	null	null	4364	2.570951939	-0.11211049	No
row_378	Nars	null	null	4390	2.530139685	-0.11384915	No
row_379	Clpb	null	null	4413	2.486188412	-0.11528846	No
row_380	Ndufv1	null	null	4416	2.484201193	-0.11473216	No
row_381	Immt	null	null	4431	2.461073875	-0.11537323	No
row_382	Gk5	null	null	4470	2.401633263	-0.11840903	No
row_383	Azin2	null	null	4475	2.397264957	-0.1180523	No
row_384	Tmem173	null	null	4482	2.391468525	-0.11789512	No
row_385	Cox10	null	null	4497	2.367503643	-0.11853619	No
row_386	Fastkd1	null	null	4498	2.365339279	-0.11778033	No
row_387	Mrps18b	null	null	4504	2.353528023	-0.11752337	No
row_388	Noa1	null	null	4511	2.341440678	-0.1173662	No
row_389	Rbfa	null	null	4516	2.326744795	-0.11700946	No
row_390	Sfxn3	null	null	4523	2.317281723	-0.11685229	No
row_391	Mrpl38	null	null	4526	2.314575672	-0.11629599	No
row_392	Ldhb	null	null	4527	2.311837673	-0.11554013	No
row_393	Hars2	null	null	4528	2.311443329	-0.11478428	No
row_394	Bckdk	null	null	4547	2.279392481	-0.11582447	No
row_395	Dhodh	null	null	4553	2.27401948	-0.11556751	No
row_396	Abca8b	null	null	4559	2.268688917	-0.11531056	No

row_397	Mapk9	null	null	4564	2.265136719	-0.11495382	No
row_398	Uqcrc1	null	null	4584	2.235046864	-0.11609379	No
row_399	Bckdhb	null	null	4589	2.23006773	-0.11573706	No
row_400	Fam110b	null	null	4591	2.227714062	-0.115080975	No
row_401	Bcat1	null	null	4604	2.207109928	-0.11552249	No
row_402	Pck2	null	null	4628	2.182816744	-0.11706158	No
row_403	Adck5	null	null	4638	2.167192221	-0.11720374	No
row_404	Mul1	null	null	4640	2.163887501	-0.11654767	No
row_405	Nrgn	null	null	4641	2.162825584	-0.11579181	No
row_406	Aldoa	null	null	4648	2.149488688	-0.115634635	No
row_407	Slc27a2	null	null	4650	2.148413181	-0.11497856	No
row_408	Pla2g6	null	null	4665	2.128335238	-0.11561963	No
row_409	Rab3d	null	null	4668	2.126123905	-0.11506333	No
row_410	Rap1gds1	null	null	4670	2.12301445	-0.11440725	No
row_411	Idh2	null	null	4676	2.118370771	-0.11415029	No
row_412	Primpol	null	null	4683	2.097679615	-0.11399312	No
row_413	Hmgcl	null	null	4686	2.089436054	-0.113436826	No
row_414	Mccc2	null	null	4692	2.083467722	-0.11317987	No
row_415	Stom	null	null	4714	2.066258669	-0.1145194	No
row_416	Zfp202	null	null	4718	2.063120127	-0.11406288	No
row_417	Idh3a	null	null	4727	2.050417185	-0.11410527	No
row_418	Sirt2	null	null	4730	2.043070316	-0.11354897	No
row_419	Acadvl	null	null	4738	2.027019978	-0.11349158	No
row_420	Rtn4ip1	null	null	4754	2.014859438	-0.11423243	No
row_421	Acot8	null	null	4759	2.007431984	-0.113875695	No
row_422	Oat	null	null	4797	1.95788765	-0.116811715	No
row_423	Ubiad1	null	null	4801	1.953627944	-0.116355196	No
row_424	Tufm	null	null	4805	1.948068261	-0.115898676	No
row_425	Taco1	null	null	4817	1.941556931	-0.116240405	No
row_426	Agpat4	null	null	4821	1.9382056	-0.11578389	No
row_427	1810043G02Rik	null	null	4829	1.933098078	-0.11572649	No
row_428	Bak1	null	null	4836	1.927259088	-0.11556932	No
row_429	Acad12	null	null	4846	1.91947639	-0.11571149	No
row_430	Cd3eap	null	null	4849	1.917641163	-0.11515519	No
row_431	Timm50	null	null	4854	1.9124403	-0.11479846	No
row_432	Ears2	null	null	4862	1.907261252	-0.11474106	No
row_433	Napg	null	null	4869	1.900616169	-0.11458389	No
row_434	Wwox	null	null	4881	1.87975812	-0.114925615	No
row_435	Rmnd1	null	null	4887	1.871801734	-0.11466866	No
row_436	Gfm1	null	null	4910	1.841452718	-0.11610797	No
row_437	Me3	null	null	4920	1.83217001	-0.116250135	No
row_438	Cpt2	null	null	4929	1.820766926	-0.11629252	No
row_439	Lipe	null	null	4936	1.806792021	-0.116135344	No
row_440	Trim39	null	null	4970	1.770997286	-0.118672244	No
row_441	Cyb5r1	null	null	4980	1.759034038	-0.11881441	No
row_442	Cars2	null	null	4985	1.75614512	-0.118457675	No
row_443	Fkbp4	null	null	4999	1.738030791	-0.11899897	No
row_444	Rhbdd1	null	null	5009	1.726055264	-0.11914113	No
row_445	Ogg1	null	null	5013	1.720508337	-0.11868461	No
row_446	Tmbim6	null	null	5028	1.702496767	-0.11932568	No
row_447	Agk	null	null	5029	1.702414751	-0.11856982	No
row_448	Acadm	null	null	5041	1.695208192	-0.11891155	No
row_449	Vdac1	null	null	5043	1.694457769	-0.11825547	No
row_450	Pnkp	null	null	5046	1.693071127	-0.117699176	No
row_451	Degs1	null	null	5072	1.666658759	-0.11943783	No
row_452	Coq6	null	null	5090	1.646344066	-0.12037824	No
row_453	Kyat3	null	null	5105	1.627786875	-0.12101931	No
row_454	Pnpla7	null	null	5110	1.624467611	-0.12066258	No
row_455	Casp8	null	null	5121	1.612777233	-0.12090452	No
row_456	Ckb	null	null	5133	1.604647636	-0.12124625	No
row_457	Bcat2	null	null	5137	1.601385355	-0.12078973	No
row_458	Pdk4	null	null	5140	1.598991632	-0.12023344	No
row_459	Ecsit	null	null	5153	1.592443705	-0.120674945	No
row_460	Slc9b2	null	null	5159	1.587163091	-0.12041799	No
row_461	Hspa2	null	null	5162	1.584339142	-0.11986169	No
row_462	Mief2	null	null	5189	1.556150913	-0.12170012	No
row_463	Mcl1	null	null	5202	1.548757911	-0.12214164	No

row_464	Msrb3	null	null	5223	1.531596661	-0.12338138	No
row_465	Qrsl1	null	null	5239	1.521166682	-0.12412223	No
row_466	Rsad1	null	null	5241	1.52100265	-0.12346616	No
row_467	Pgs1	null	null	5248	1.518357754	-0.12330898	No
row_468	Rnf185	null	null	5249	1.517173171	-0.122553125	No
row_469	Grsf1	null	null	5256	1.512559295	-0.12239595	No
row_470	Tmem14c	null	null	5263	1.504982233	-0.12223878	No
row_471	Hcls1	null	null	5264	1.503959656	-0.121482916	No
row_472	Tk2	null	null	5270	1.497492433	-0.12122596	No
row_473	Park2	null	null	5277	1.490501285	-0.12106878	No
row_474	Etfdh	null	null	5287	1.479205132	-0.121210955	No
row_475	Slc25a30	null	null	5303	1.464933157	-0.1219518	No
row_476	Slc27a3	null	null	5332	1.446204662	-0.1239898	No
row_477	Dhtkd1	null	null	5344	1.437472463	-0.12433153	No
row_478	Hars	null	null	5347	1.435396194	-0.12377523	No
row_479	Mmaa	null	null	5357	1.423421621	-0.12391739	No
row_480	Noc3l	null	null	5384	1.406960726	-0.12575583	No
row_481	Top1mt	null	null	5391	1.399850607	-0.12559865	No
row_482	Ppox	null	null	5401	1.394378662	-0.12574083	No
row_483	Dusp18	null	null	5407	1.388860703	-0.12548387	No
row_484	Slc25a29	null	null	5411	1.388074398	-0.12502734	No
row_485	Acad8	null	null	5412	1.387992263	-0.12427149	No
row_486	Sord	null	null	5425	1.380183816	-0.124712996	No
row_487	Galc	null	null	5426	1.379164696	-0.12395714	No
row_488	Ap2m1	null	null	5433	1.376042485	-0.123799965	No
row_489	Armc10	null	null	5454	1.360972047	-0.12503971	No
row_490	Gpd1	null	null	5457	1.35944438	-0.12448342	No
row_491	Opa1	null	null	5459	1.358728647	-0.123827346	No
row_492	Akap10	null	null	5469	1.352800012	-0.12396951	No
row_493	Cmpk2	null	null	5492	1.3377707	-0.12540883	No
row_494	Acads	null	null	5496	1.334851503	-0.12495231	No
row_495	Sod2	null	null	5500	1.331216693	-0.12449579	No
row_496	Akr7a5	null	null	5515	1.318644047	-0.12513685	No
row_497	Spata7	null	null	5517	1.318313837	-0.12448078	No
row_498	Coq10a	null	null	5520	1.315760136	-0.12392449	No
row_499	Prdx2	null	null	5522	1.312723041	-0.1232684	No
row_500	Atp5a1	null	null	5525	1.309823394	-0.122712106	No
row_501	Comt	null	null	5526	1.309430599	-0.12195625	No
row_502	Fitm2	null	null	5527	1.309401393	-0.12120039	No
row_503	Nit1	null	null	5532	1.304175615	-0.12084366	No
row_504	Atp5d	null	null	5550	1.292449474	-0.12178407	No
row_505	Amt	null	null	5554	1.290746331	-0.12132755	No
row_506	Aldoc	null	null	5562	1.285212874	-0.12127016	No
row_507	Mtfp1	null	null	5563	1.284635544	-0.120514296	No
row_508	Mapk1	null	null	5580	1.279322267	-0.12135493	No
row_509	Neu4	null	null	5582	1.278998375	-0.120698854	No
row_510	Acsl6	null	null	5588	1.278223634	-0.1204419	No
row_511	Coq9	null	null	5594	1.276125431	-0.12018494	No
row_512	Ppm1e	null	null	5602	1.271299005	-0.120127544	No
row_513	Dnajc30	null	null	5617	1.260826707	-0.120768614	No
row_514	Ddx28	null	null	5627	1.25445807	-0.12091078	No
row_515	Idh3b	null	null	5632	1.251594186	-0.120554045	No
row_516	Kyat1	null	null	5640	1.246470571	-0.12049665	No
row_517	Anxa1	null	null	5658	1.23743546	-0.12143706	No
row_518	Gapdh	null	null	5667	1.233139873	-0.121479444	No
row_519	Trmu	null	null	5682	1.224284887	-0.122120515	No
row_520	Slc25a11	null	null	5687	1.220462203	-0.12176378	No
row_521	Dlat	null	null	5689	1.219401002	-0.121107705	No
row_522	D10Jhu81e	null	null	5692	1.217714787	-0.12055141	No
row_523	Abcb10	null	null	5704	1.211159587	-0.120893136	No
row_524	Adck1	null	null	5723	1.201917648	-0.121933326	No
row_525	Slc25a13	null	null	5725	1.20121789	-0.12127724	No
row_526	Slc25a27	null	null	5726	1.200457454	-0.12052139	No
row_527	Mtx1	null	null	5730	1.200089931	-0.12006487	No
row_528	Ndufa10	null	null	5737	1.191801667	-0.1199077	No
row_529	Pdss2	null	null	5743	1.18592751	-0.119650744	No
row_530	Slc25a37	null	null	5745	1.185130358	-0.11899466	No

row_531	Pycr2	null	null	5750	1.181620002	-0.11863793	No
row_532	Mrpl45	null	null	5758	1.177889824	-0.118580535	No
row_533	Dap3	null	null	5767	1.17144978	-0.11862292	No
row_534	Tamm41	null	null	5769	1.170014977	-0.11796684	No
row_535	Ckmt1	null	null	5796	1.155171037	-0.11980528	No
row_536	Slc25a1	null	null	5797	1.154243827	-0.119049415	No
row_537	Dus2	null	null	5804	1.151184559	-0.118892245	No
row_538	Lyn	null	null	5810	1.148291945	-0.11863529	No
row_539	Suox	null	null	5811	1.147613883	-0.11787943	No
row_540	Fdxr	null	null	5813	1.146381378	-0.11722335	No
row_541	Tomm40	null	null	5814	1.145564079	-0.11646749	No
row_542	Slc30a6	null	null	5817	1.14279294	-0.1159112	No
row_543	Esr1	null	null	5818	1.14243114	-0.11515534	No
row_544	Map1lc3b	null	null	5844	1.132220149	-0.11689399	No
row_545	As3mt	null	null	5855	1.129204988	-0.11713594	No
row_546	Aco2	null	null	5856	1.12805295	-0.11638008	No
row_547	Tmtc1	null	null	5859	1.126435637	-0.11582378	No
row_548	Chat	null	null	5868	1.120303035	-0.11586617	No
row_549	Acaa1a	null	null	5875	1.118386984	-0.115709	No
row_550	Braf	null	null	5881	1.116573691	-0.11545204	No
row_551	Rhot1	null	null	5882	1.116189241	-0.11469618	No
row_552	C1qbp	null	null	5895	1.111651301	-0.11513769	No
row_553	Ubb	null	null	5906	1.106663108	-0.11537964	No
row_554	Mrpl37	null	null	5908	1.105024099	-0.11472356	No
row_555	Pdhx	null	null	5936	1.086140275	-0.11666177	No
row_556	Cnp	null	null	5945	1.083652258	-0.11670416	No
row_557	Ndufs2	null	null	5952	1.079359531	-0.11654699	No
row_558	Capn10	null	null	5957	1.077243924	-0.11619025	No
row_559	Ndufs1	null	null	5965	1.070915699	-0.116132855	No
row_560	Cct7	null	null	5968	1.069877267	-0.11557656	No
row_561	Sfxn5	null	null	5978	1.065988302	-0.11571872	No
row_562	Vamp1	null	null	5985	1.062066674	-0.11556155	No
row_563	Dcps	null	null	5990	1.059567809	-0.11520481	No
row_564	Iars2	null	null	6002	1.054593205	-0.11554654	No
row_565	Card19	null	null	6018	1.046981692	-0.11628739	No
row_566	Bag5	null	null	6025	1.043227315	-0.11613022	No
row_567	Isoc2b	null	null	6030	1.041677117	-0.11577348	No
row_568	Ddah2	null	null	6052	1.029665828	-0.11711301	No
row_569	Mlycd	null	null	6065	1.021879435	-0.11755452	No
row_570	Rhoa	null	null	6094	1.011967897	-0.11959252	No
row_571	Ak2	null	null	6105	1.007686615	-0.11983446	No
row_572	Mtg2	null	null	6111	1.00466454	-0.119577505	No
row_573	Mthfd2	null	null	6116	1.003046393	-0.11922077	No
row_574	Mapk8ip1	null	null	6121	-1.002788782	-0.11886404	No
row_575	Dhrs1	null	null	6126	-1.004933119	-0.118507296	No
row_576	Lrrc59	null	null	6130	-1.006840706	-0.118050784	No
row_577	Slc1a3	null	null	6131	-1.006961346	-0.11729492	No
row_578	Gfm2	null	null	6139	-1.009041786	-0.11723753	No
row_579	Sars	null	null	6164	-1.019652843	-0.118876405	No
row_580	Slc25a3	null	null	6174	-1.02440083	-0.11901857	No
row_581	Foxred1	null	null	6175	-1.02528739	-0.118262716	No
row_582	Nudt6	null	null	6177	-1.027190328	-0.11760663	No
row_583	Abca9	null	null	6215	-1.043434143	-0.12054265	No
row_584	Eral1	null	null	6220	-1.045260549	-0.12018592	No
row_585	Map2k1	null	null	6221	-1.045526147	-0.11943006	No
row_586	Kars	null	null	6222	-1.046314955	-0.118674204	No
row_587	P4ha1	null	null	6283	-1.074584723	-0.123905174	No
row_588	Letmd1	null	null	6297	-1.078640223	-0.12444646	No
row_589	Alkbh1	null	null	6298	-1.07873404	-0.123690605	No
row_590	Tmem186	null	null	6304	-1.082322001	-0.12343365	No
row_591	Slc25a18	null	null	6307	-1.08379066	-0.12287735	No
row_592	Fech	null	null	6314	-1.08719945	-0.122720174	No
row_593	Aass	null	null	6329	-1.094418883	-0.123361245	No
row_594	Rpusd4	null	null	6335	-1.097349644	-0.12310429	No
row_595	Mgea5	null	null	6346	-1.103762507	-0.12334624	No
row_596	Mtif3	null	null	6352	-1.108437538	-0.12308928	No
row_597	Acaa2	null	null	6360	-1.11532402	-0.123031884	No

row_598	Ciapin1	null	null	6377	-1.127071023	-0.12387252	No
row_599	Abhd12	null	null	6380	-1.130044341	-0.12331622	No
row_600	Parl	null	null	6385	-1.136977553	-0.12295949	No
row_601	Comtd1	null	null	6391	-1.140025616	-0.12270253	No
row_602	Nt5m	null	null	6396	-1.141565681	-0.12234579	No
row_603	Mrpl44	null	null	6398	-1.143575311	-0.121689714	No
row_604	Slc25a15	null	null	6399	-1.144333482	-0.12093386	No
row_605	Hoxb9	null	null	6421	-1.151726842	-0.12227339	No
row_606	Prodh	null	null	6430	-1.157647252	-0.12231577	No
row_607	Tmem135	null	null	6437	-1.162997842	-0.1221586	No
row_608	Phykpl	null	null	6448	-1.171460152	-0.122400545	No
row_609	Mipep	null	null	6453	-1.176584721	-0.12204381	No
row_610	Slc16a1	null	null	6455	-1.178070307	-0.121387735	No
row_611	Rars	null	null	6464	-1.182522893	-0.12143012	No
row_612	Micu1	null	null	6496	-1.194515228	-0.12376746	No
row_613	Acbd3	null	null	6503	-1.200517297	-0.12361028	No
row_614	Spryd4	null	null	6514	-1.209576726	-0.12385223	No
row_615	Coq4	null	null	6517	-1.211387992	-0.12329593	No
row_616	Pemt	null	null	6535	-1.221538305	-0.124236345	No
row_617	Atg4d	null	null	6538	-1.222115278	-0.12368005	No
row_618	Mpv17l2	null	null	6543	-1.227466464	-0.12332331	No
row_619	Mapk12	null	null	6562	-1.240274906	-0.1243635	No
row_620	Pars2	null	null	6568	-1.245190024	-0.12410654	No
row_621	Stk11	null	null	6587	-1.256942987	-0.12514673	No
row_622	Cep89	null	null	6591	-1.259579659	-0.12469022	No
row_623	Mfn1	null	null	6592	-1.26042521	-0.12393436	No
row_624	Slc25a20	null	null	6595	-1.261403322	-0.12337806	No
row_625	Dguok	null	null	6607	-1.272910595	-0.12371979	No
row_626	Bcl2l1	null	null	6652	-1.305274606	-0.12735428	No
row_627	Mtfr1l	null	null	6661	-1.309640169	-0.12739666	No
row_628	Ehhadh	null	null	6664	-1.310539365	-0.12684037	No
row_629	Rad51	null	null	6666	-1.312627792	-0.12618428	No
row_630	Ndufaf7	null	null	6673	-1.316715837	-0.1260271	No
row_631	Ppp1r15a	null	null	6685	-1.325673103	-0.12636884	No
row_632	Mcur1	null	null	6694	-1.332969666	-0.12641123	No
row_633	Nktr	null	null	6705	-1.341600895	-0.12665316	No
row_634	Slc25a34	null	null	6715	-1.344793439	-0.12679534	No
row_635	Adap2	null	null	6740	-1.361662388	-0.12843421	No
row_636	Gsr	null	null	6745	-1.365924597	-0.12807748	No
row_637	Pxmp2	null	null	6749	-1.369105697	-0.12762097	No
row_638	Mrpl10	null	null	6750	-1.36965251	-0.1268651	No
row_639	Pecr	null	null	6755	-1.376085997	-0.12650837	No
row_640	Ppp3ca	null	null	6763	-1.386181116	-0.12645097	No
row_641	Ncoa4	null	null	6788	-1.411063433	-0.12808985	No
row_642	Oxsm	null	null	6790	-1.411330462	-0.12743376	No
row_643	Glud1	null	null	6821	-1.439887285	-0.12967132	No
row_644	Prr5l	null	null	6837	-1.452314854	-0.13041218	No
row_645	Fastkd3	null	null	6840	-1.453123569	-0.12985587	No
row_646	Ndufs3	null	null	6847	-1.457847953	-0.1296987	No
row_647	Cav1	null	null	6852	-1.460912585	-0.12934196	No
row_648	Atp6v1a	null	null	6858	-1.467097759	-0.129085	No
row_649	Slc25a39	null	null	6859	-1.471047163	-0.12832916	No
row_650	Hacd3	null	null	6872	-1.480469465	-0.12877066	No
row_651	Slc25a45	null	null	6875	-1.486242294	-0.12821436	No
row_652	Mcat	null	null	6888	-1.496676207	-0.12865587	No
row_653	Ykt6	null	null	6908	-1.52192688	-0.12979583	No
row_654	Phyh	null	null	6911	-1.523732901	-0.12923954	No
row_655	Mrpl43	null	null	6913	-1.524495244	-0.12858346	No
row_656	Brinp3	null	null	6925	-1.539889574	-0.12892519	No
row_657	Mrpl28	null	null	6929	-1.549821138	-0.12846868	No
row_658	Coasy	null	null	6951	-1.569150925	-0.12980822	No
row_659	Cyba	null	null	6952	-1.569957852	-0.12905236	No
row_660	Adprhl2	null	null	6957	-1.573737621	-0.12869562	No
row_661	Spr	null	null	6973	-1.591120243	-0.12943646	No
row_662	Rab24	null	null	6980	-1.598194957	-0.12927929	No
row_663	Slc25a24	null	null	6982	-1.598283172	-0.12862322	No
row_664	1600014C10Rik	null	null	6983	-1.598754764	-0.12786736	No

row_665	Zadh2	null	null	6998	-1.614399076	-0.12850842	No
row_666	Hspd1	null	null	6999	-1.615206599	-0.12775257	No
row_667	Gpt2	null	null	7007	-1.621333003	-0.12769517	No
row_668	Coq5	null	null	7026	-1.643521428	-0.12873536	No
row_669	Poldip2	null	null	7029	-1.650955677	-0.12817906	No
row_670	Reep1	null	null	7030	-1.651575446	-0.12742321	No
row_671	Vdac2	null	null	7039	-1.659523606	-0.12746559	No
row_672	Slc25a17	null	null	7048	-1.673672318	-0.12750798	No
row_673	Amacr	null	null	7057	-1.68515408	-0.12755036	No
row_674	Nif3l1	null	null	7063	-1.696105123	-0.12729341	No
row_675	Coa7	null	null	7067	-1.697702169	-0.1268369	No
row_676	Slc25a19	null	null	7068	-1.697833776	-0.12608103	No
row_677	Acadl	null	null	7079	-1.719028473	-0.12632298	No
row_678	Sco1	null	null	7106	-1.749349475	-0.12816142	No
row_679	Gpn1	null	null	7132	-1.778760672	-0.12990007	No
row_680	Atpaf2	null	null	7149	-1.801508069	-0.1307407	No
row_681	Gatb	null	null	7161	-1.822108865	-0.13108243	No
row_682	Bid	null	null	7162	-1.822134972	-0.13032657	No
row_683	Grpel2	null	null	7168	-1.830789804	-0.13006961	No
row_684	Gstp1	null	null	7181	-1.857334137	-0.13051112	No
row_685	Vrk2	null	null	7208	-1.885925293	-0.13234955	No
row_686	Slc25a28	null	null	7210	-1.887192369	-0.13169348	No
row_687	Gtpbp8	null	null	7219	-1.905473471	-0.13173586	No
row_688	Xrcc3	null	null	7235	-1.936885834	-0.13247672	No
row_689	Abhd6	null	null	7238	-1.938058019	-0.13192041	No
row_690	Chchd10	null	null	7248	-1.955937743	-0.13206258	No
row_691	Mrpl49	null	null	7286	-2.00241971	-0.1349986	No
row_692	Hadh	null	null	7295	-2.012938261	-0.13504098	No
row_693	Trmt1	null	null	7312	-2.040254831	-0.13588162	No
row_694	Slc44a1	null	null	7317	-2.050876141	-0.13552488	No
row_695	Sdsl	null	null	7330	-2.062828541	-0.13596639	No
row_696	Mtpap	null	null	7333	-2.067467928	-0.1354101	No
row_697	Bcl2	null	null	7349	-2.09152174	-0.13615094	No
row_698	Nol3	null	null	7359	-2.119362116	-0.13629311	No
row_699	Ptges2	null	null	7362	-2.124014854	-0.13573681	No
row_700	Golph3	null	null	7364	-2.126166821	-0.13508074	No
row_701	Hax1	null	null	7369	-2.143068552	-0.134724	No
row_702	Abhd11	null	null	7383	-2.170074701	-0.13526529	No
row_703	Caprin2	null	null	7390	-2.178168774	-0.13510811	No
row_704	Bcs1l	null	null	7405	-2.208737612	-0.13574918	No
row_705	Mrps30	null	null	7406	-2.212972641	-0.13499333	No
row_706	Ptcd3	null	null	7407	-2.213463545	-0.13423747	No
row_707	Mrpl2	null	null	7421	-2.225229502	-0.13477875	No
row_708	Ociad1	null	null	7425	-2.228018761	-0.13432224	No
row_709	Mrps7	null	null	7426	-2.229056597	-0.13356638	No
row_710	Sgk1	null	null	7440	-2.251292467	-0.13410766	No
row_711	Atp5b	null	null	7451	-2.269165754	-0.13434961	No
row_712	Nars2	null	null	7466	-2.285134792	-0.13499069	No
row_713	Gcat	null	null	7477	-2.299731016	-0.13523263	No
row_714	Thg1l	null	null	7488	-2.319569111	-0.13547458	No
row_715	Mtch1	null	null	7499	-2.34424758	-0.13571653	No
row_716	Ccdc51	null	null	7504	-2.355716467	-0.1353598	No
row_717	Mrps18a	null	null	7513	-2.375406742	-0.13540217	No
row_718	Mrps12	null	null	7532	-2.407607555	-0.13644236	Yes
row_719	Map2k2	null	null	7536	-2.416812181	-0.13598585	Yes
row_720	Trmt5	null	null	7544	-2.427974939	-0.13592845	Yes
row_721	Nat8f1	null	null	7548	-2.429358482	-0.13547194	Yes
row_722	Gabarapl1	null	null	7552	-2.440652609	-0.13501543	Yes
row_723	Cd36	null	null	7559	-2.46012187	-0.13485825	Yes
row_724	Macrod1	null	null	7570	-2.476816654	-0.1351002	Yes
row_725	Ybey	null	null	7572	-2.47964263	-0.13444412	Yes
row_726	Mrm2	null	null	7573	-2.480162144	-0.13368826	Yes
row_727	Hnrnpk	null	null	7574	-2.485645771	-0.1329324	Yes
row_728	Tdrkh	null	null	7578	-2.502861023	-0.13247588	Yes
row_729	Mapk3	null	null	7584	-2.521585226	-0.13221893	Yes
row_730	Lap3	null	null	7588	-2.523762465	-0.13176242	Yes
row_731	Nt5c	null	null	7589	-2.527381182	-0.13100655	Yes

row_732	Tango2	null	null	7590	-2.531311512	-0.13025069	Yes
row_733	Pacrg	null	null	7595	-2.543083191	-0.12989396	Yes
row_734	Ngb	null	null	7608	-2.583889723	-0.13033547	Yes
row_735	Oxct1	null	null	7614	-2.594003201	-0.13007851	Yes
row_736	Oxnad1	null	null	7615	-2.594465256	-0.12932266	Yes
row_737	Mmadhc	null	null	7632	-2.646801472	-0.13016328	Yes
row_738	Dars2	null	null	7635	-2.662145615	-0.12960699	Yes
row_739	Ifit3b	null	null	7645	-2.676638603	-0.12974915	Yes
row_740	Mgme1	null	null	7655	-2.692664146	-0.12989132	Yes
row_741	Tsfm	null	null	7661	-2.711207151	-0.12963437	Yes
row_742	Mrpl3	null	null	7668	-2.72729826	-0.12947719	Yes
row_743	Oma1	null	null	7685	-2.774160147	-0.13031782	Yes
row_744	Mrpl19	null	null	7721	-2.858035564	-0.13305429	Yes
row_745	Lgals3	null	null	7729	-2.878502607	-0.13299689	Yes
row_746	Rab1b	null	null	7730	-2.883603811	-0.13224103	Yes
row_747	Fez1	null	null	7732	-2.887315273	-0.13158494	Yes
row_748	Mtif2	null	null	7741	-2.906193018	-0.13162734	Yes
row_749	Gls	null	null	7742	-2.906574726	-0.13087147	Yes
row_750	Myoc	null	null	7746	-2.915161133	-0.13041496	Yes
row_751	Hebp1	null	null	7761	-2.951076508	-0.13105603	Yes
row_752	Ddx6	null	null	7770	-2.972809076	-0.13109842	Yes
row_753	Uxs1	null	null	7777	-2.982473135	-0.13094124	Yes
row_754	Gadd45gip1	null	null	7784	-2.999709368	-0.13078406	Yes
row_755	Lias	null	null	7795	-3.025215864	-0.13102601	Yes
row_756	Lars2	null	null	7797	-3.02647233	-0.13036993	Yes
row_757	Sfxn1	null	null	7801	-3.042882681	-0.12991342	Yes
row_758	Serhl	null	null	7816	-3.084194422	-0.13055448	Yes
row_759	Mtfr1	null	null	7839	-3.157846689	-0.1319938	Yes
row_760	Bsg	null	null	7850	-3.185370445	-0.13223575	Yes
row_761	Smim20	null	null	7853	-3.193604946	-0.13167945	Yes
row_762	Uqcc1	null	null	7860	-3.214009762	-0.13152227	Yes
row_763	Hagh	null	null	7870	-3.242093801	-0.13166444	Yes
row_764	Dut	null	null	7876	-3.261046648	-0.13140748	Yes
row_765	Tspo	null	null	7884	-3.293490171	-0.13135009	Yes
row_766	Hspa5	null	null	7891	-3.312300205	-0.13119292	Yes
row_767	Eci2	null	null	7922	-3.382910252	-0.13343047	Yes
row_768	Suclg2	null	null	7923	-3.387673616	-0.13267462	Yes
row_769	Hif3a	null	null	7931	-3.402850151	-0.13261722	Yes
row_770	Pdia3	null	null	7936	-3.419475794	-0.13226049	Yes
row_771	Ldha	null	null	7939	-3.422772884	-0.13170418	Yes
row_772	L2hgdh	null	null	7950	-3.451996088	-0.13194613	Yes
row_773	Chchd5	null	null	7952	-3.456046343	-0.13129006	Yes
row_774	Ttc19	null	null	7974	-3.519971371	-0.13262959	Yes
row_775	Qtrt1	null	null	7989	-3.580135345	-0.13327065	Yes
row_776	Gm2a	null	null	7991	-3.585829735	-0.13261458	Yes
row_777	Slc25a26	null	null	8021	-3.703247547	-0.13475236	Yes
row_778	Pmpcb	null	null	8028	-3.731791258	-0.13459519	Yes
row_779	Lactb	null	null	8034	-3.755672455	-0.13433823	Yes
row_780	Mrpl9	null	null	8043	-3.780286789	-0.13438061	Yes
row_781	Cyc1	null	null	8053	-3.818825006	-0.13452278	Yes
row_782	Arl2	null	null	8055	-3.824611902	-0.1338667	Yes
row_783	Cryab	null	null	8058	-3.831045866	-0.1333104	Yes
row_784	Slc35b3	null	null	8066	-3.858414173	-0.13325301	Yes
row_785	Mfsd7b	null	null	8080	-3.907736301	-0.1337943	Yes
row_786	Tatdn3	null	null	8104	-4.008947849	-0.13533339	Yes
row_787	Mpg	null	null	8105	-4.013735294	-0.13457753	Yes
row_788	Rab8b	null	null	8106	-4.022218227	-0.13382168	Yes
row_789	Bnip1	null	null	8108	-4.024753571	-0.1331656	Yes
row_790	Polg2	null	null	8112	-4.044993401	-0.13270909	Yes
row_791	Msra	null	null	8117	-4.060251713	-0.13235235	Yes
row_792	Endog	null	null	8122	-4.094445229	-0.1319956	Yes
row_793	Pebp1	null	null	8132	-4.130330563	-0.13213778	Yes
row_794	Apex1	null	null	8134	-4.132324696	-0.13148169	Yes
row_795	Ppargc1a	null	null	8144	-4.17288065	-0.13162386	Yes
row_796	Ywhae	null	null	8146	-4.179256916	-0.13096778	Yes
row_797	Uqcc3	null	null	8148	-4.184010506	-0.13031171	Yes
row_798	Slc22a4	null	null	8162	-4.248437405	-0.130853	Yes

row_799	Dusp26	null	null	8167	-4.274590015	-0.13049626	Yes
row_800	Stard4	null	null	8173	-4.314545155	-0.13023931	Yes
row_801	Adhfe1	null	null	8179	-4.331067085	-0.12998235	Yes
row_802	Mrpl34	null	null	8181	-4.340406895	-0.12932627	Yes
row_803	Mrps9	null	null	8186	-4.361042976	-0.12896954	Yes
row_804	Ppp1cc	null	null	8191	-4.377094746	-0.1286128	Yes
row_805	Timm17a	null	null	8192	-4.390330315	-0.12785694	Yes
row_806	Fbxw7	null	null	8194	-4.403601646	-0.12720087	Yes
row_807	Xaf1	null	null	8214	-4.521070957	-0.12834084	Yes
row_808	Fas	null	null	8235	-4.607834339	-0.12958059	Yes
row_809	Ethe1	null	null	8236	-4.611383915	-0.12882473	Yes
row_810	Stard6	null	null	8238	-4.620233059	-0.12816866	Yes
row_811	Fbxo7	null	null	8239	-4.634056091	-0.1274128	Yes
row_812	Dbt	null	null	8241	-4.640494347	-0.12675671	Yes
row_813	Gstz1	null	null	8243	-4.658217907	-0.12610064	Yes
row_814	Dnajc4	null	null	8262	-4.771062851	-0.12714083	Yes
row_815	Tcaim	null	null	8265	-4.782747746	-0.12658453	Yes
row_816	Clic1	null	null	8269	-4.803500652	-0.12612802	Yes
row_817	Mrps11	null	null	8273	-4.816332817	-0.1256715	Yes
row_818	Becn1	null	null	8299	-4.961402893	-0.12741016	Yes
row_819	Trak2	null	null	8308	-5.019854069	-0.12745254	Yes
row_820	Auh	null	null	8312	-5.043000698	-0.12699603	Yes
row_821	Abcg2	null	null	8315	-5.060030937	-0.12643974	Yes
row_822	Fxn	null	null	8317	-5.065469265	-0.12578365	Yes
row_823	Slc25a33	null	null	8332	-5.131250858	-0.12642471	Yes
row_824	Mrpl16	null	null	8337	-5.149276733	-0.12606798	Yes
row_825	Nudt9	null	null	8338	-5.149444103	-0.12531212	Yes
row_826	Ppp2r2b	null	null	8339	-5.151051998	-0.124556266	Yes
row_827	Etnppl	null	null	8341	-5.153329372	-0.12390019	Yes
row_828	Suclg1	null	null	8349	-5.188228607	-0.1238428	Yes
row_829	1700021F05Rik	null	null	8357	-5.228873253	-0.12378541	Yes
row_830	Pdk1	null	null	8362	-5.238944054	-0.123428665	Yes
row_831	Mrps26	null	null	8364	-5.248119354	-0.12277259	Yes
row_832	Cyp27a1	null	null	8386	-5.396493435	-0.12411212	Yes
row_833	Cyp1b1	null	null	8390	-5.409810066	-0.1236556	Yes
row_834	Rpp14	null	null	8391	-5.413635254	-0.12289975	Yes
row_835	Bad	null	null	8394	-5.426966667	-0.12234345	Yes
row_836	Slc25a32	null	null	8416	-5.643331051	-0.12368298	Yes
row_837	Atp6v1e1	null	null	8417	-5.647609234	-0.12292712	Yes
row_838	Hrk	null	null	8418	-5.650303364	-0.12217127	Yes
row_839	Ghr	null	null	8429	-5.739111423	-0.12241321	Yes
row_840	Ucp3	null	null	8430	-5.744561672	-0.12165736	Yes
row_841	Hemk1	null	null	8432	-5.756567478	-0.12100128	Yes
row_842	Tfb2m	null	null	8433	-5.757839203	-0.12024542	Yes
row_843	Mars2	null	null	8439	-5.782354355	-0.119988464	Yes
row_844	Sgpp1	null	null	8441	-5.804055214	-0.11933239	Yes
row_845	Ppid	null	null	8458	-5.903687477	-0.120173015	Yes
row_846	2810006K23Rik	null	null	8469	-5.951928139	-0.120414965	Yes
row_847	Abcd2	null	null	8473	-5.982910156	-0.119958445	Yes
row_848	Tusc2	null	null	8481	-6.035149574	-0.11990105	Yes
row_849	Ndufs7	null	null	8482	-6.047410011	-0.11914519	Yes
row_850	Bax	null	null	8511	-6.23724699	-0.121183194	Yes
row_851	Yrdc	null	null	8520	-6.316610336	-0.12122557	Yes
row_852	Ercc6l2	null	null	8531	-6.360164165	-0.12146752	Yes
row_853	Mrps25	null	null	8533	-6.375924587	-0.12081145	Yes
row_854	Ifit3	null	null	8534	-6.377792358	-0.120055586	Yes
row_855	Prelid1	null	null	8545	-6.440027237	-0.120297536	Yes
row_856	Cyb5b	null	null	8548	-6.469127178	-0.11974124	Yes
row_857	Vhl	null	null	8550	-6.472899914	-0.11908516	Yes
row_858	Rad51c	null	null	8552	-6.47774744	-0.11842908	Yes
row_859	Dnm1l	null	null	8558	-6.518179417	-0.11817213	Yes
row_860	Fars2	null	null	8563	-6.550168037	-0.11781539	Yes
row_861	Txn2	null	null	8574	-6.604828358	-0.11805734	Yes
row_862	Lactb2	null	null	8576	-6.652994633	-0.117401265	Yes
row_863	Mrps23	null	null	8588	-6.735428333	-0.117742985	Yes
row_864	Dnlz	null	null	8612	-6.907063007	-0.11928208	Yes
row_865	Cmc2	null	null	8620	-6.951449871	-0.11922469	Yes

row_866	Coa4	null	null	8621	-6.956696987	-0.11846883	Yes
row_867	Sirt1	null	null	8627	-6.991393089	-0.11821187	Yes
row_868	Mrps35	null	null	8642	-7.18482542	-0.11885294	Yes
row_869	Pak7	null	null	8645	-7.221498489	-0.118296646	Yes
row_870	Cfl1	null	null	8646	-7.234813213	-0.11754079	Yes
row_871	Tmem11	null	null	8648	-7.24037838	-0.11688471	Yes
row_872	Aldh6a1	null	null	8657	-7.305575848	-0.116927095	Yes
row_873	Uri1	null	null	8668	-7.365706921	-0.117169045	Yes
row_874	Ppa2	null	null	8673	-7.409092903	-0.1168123	Yes
row_875	Pold4	null	null	8675	-7.425961971	-0.11615623	Yes
row_876	Tppp	null	null	8679	-7.450469971	-0.115699716	Yes
row_877	Mpst	null	null	8682	-7.481423855	-0.11514342	Yes
row_878	Nudt1	null	null	8706	-7.744891644	-0.11668251	Yes
row_879	Mtg1	null	null	8715	-7.807396889	-0.11672489	Yes
row_880	Gsta4	null	null	8725	-7.868164539	-0.116867065	Yes
row_881	Higd2a	null	null	8729	-7.905550957	-0.116410546	Yes
row_882	Sh3glb1	null	null	8745	-8.053488731	-0.117151394	Yes
row_883	Tfb1m	null	null	8751	-8.152229309	-0.11689444	Yes
row_884	Fkbp8	null	null	8761	-8.301417351	-0.1170366	Yes
row_885	Ndufa9	null	null	8773	-8.412945747	-0.11737833	Yes
row_886	Cox5a	null	null	8790	-8.647891998	-0.118218966	Yes
row_887	Gja1	null	null	8793	-8.680226326	-0.11766267	Yes
row_888	Pon2	null	null	8800	-8.743035316	-0.11750549	Yes
row_889	Pcca	null	null	8806	-8.781692505	-0.117248535	Yes
row_890	Mrpl14	null	null	8821	-8.893301964	-0.117889605	Yes
row_891	Cox6b2	null	null	8834	-9.038023949	-0.11833111	Yes
row_892	Mapk8	null	null	8836	-9.050470352	-0.117675036	Yes
row_893	Ak4	null	null	8837	-9.071762085	-0.116919175	Yes
row_894	Dhrs4	null	null	8844	-9.149191856	-0.116762005	Yes
row_895	Cisd1	null	null	8846	-9.166194916	-0.11610592	Yes
row_896	Clpp	null	null	8851	-9.22636795	-0.11574919	Yes
row_897	Cpne3	null	null	8860	-9.343126297	-0.115791574	Yes
row_898	Rars2	null	null	8882	-9.572128296	-0.11713111	Yes
row_899	Tst	null	null	8886	-9.602250099	-0.11667459	Yes
row_900	Me1	null	null	8894	-9.689884186	-0.116617195	Yes
row_901	Abcb1b	null	null	8901	-9.855746269	-0.11646002	Yes
row_902	Ranbp2	null	null	8903	-9.886762619	-0.11580394	Yes
row_903	Aim2	null	null	8911	-10.0067873	-0.11574655	Yes
row_904	Cpox	null	null	8916	-10.08503819	-0.11538981	Yes
row_905	Mrrf	null	null	8970	-10.69846153	-0.11992232	Yes
row_906	Ptcd2	null	null	8997	-11.09209251	-0.121760756	Yes
row_907	Mrps17	null	null	9010	-11.25024986	-0.12220226	Yes
row_908	Pten	null	null	9019	-11.41794682	-0.12224465	Yes
row_909	Stoml2	null	null	9020	-11.44312763	-0.12148879	Yes
row_910	Sdhaf2	null	null	9022	-11.44784069	-0.12083271	Yes
row_911	Cox11	null	null	9025	-11.46237469	-0.120276414	Yes
row_912	Fh1	null	null	9034	-11.57426643	-0.1203188	Yes
row_913	Erbb4	null	null	9035	-11.57727432	-0.119562946	Yes
row_914	Fbxl4	null	null	9047	-11.70630932	-0.119904675	Yes
row_915	Cox7a1	null	null	9050	-11.77324963	-0.11934838	Yes
row_916	Plgrkt	null	null	9061	-11.94862652	-0.11959032	Yes
row_917	Tmem65	null	null	9067	-12.0536499	-0.119333364	Yes
row_918	Arl2bp	null	null	9079	-12.26266003	-0.11967509	Yes
row_919	Nme6	null	null	9106	-12.6298542	-0.12151353	Yes
row_920	Ccm2	null	null	9127	-12.82771969	-0.12275328	Yes
row_921	Clpx	null	null	9162	-13.47883701	-0.12538996	Yes
row_922	Pcf11	null	null	9169	-13.53768921	-0.12523279	Yes
row_923	Guf1	null	null	9175	-13.58656788	-0.12497583	Yes
row_924	Gatc	null	null	9191	-13.758255	-0.12571667	Yes
row_925	Ppm1k	null	null	9192	-13.7719965	-0.12496082	Yes
row_926	Snn	null	null	9197	-13.88101292	-0.12460408	Yes
row_927	Rfk	null	null	9199	-13.94225788	-0.12394801	Yes
row_928	Nipsnap3b	null	null	9210	-14.26474953	-0.12418995	Yes
row_929	Cisd2	null	null	9225	-14.56065559	-0.12483102	Yes
row_930	Fahd1	null	null	9227	-14.56924629	-0.124174945	Yes
row_931	Casp8ap2	null	null	9236	-14.87312126	-0.12421733	Yes
row_932	Glyat	null	null	9237	-14.89055157	-0.12346147	Yes

row_933	Bdnf	null	null	9249	-15.04542065	-0.1238032	Yes
row_934	Tomm20	null	null	9259	-15.21238327	-0.12394536	Yes
row_935	Phb	null	null	9276	-15.61261749	-0.124786	Yes
row_936	Pgr	null	null	9287	-15.77380753	-0.12502794	Yes
row_937	Park7	null	null	9289	-15.90094566	-0.124371864	Yes
row_938	Dctn6	null	null	9293	-15.98540688	-0.12391535	Yes
row_939	Coa5	null	null	9298	-16.07702827	-0.12355861	Yes
row_940	Csde1	null	null	9310	-16.42247772	-0.12390034	Yes
row_941	Ptgr2	null	null	9311	-16.43027115	-0.123144485	Yes
row_942	Uchl5	null	null	9318	-16.54318428	-0.12298731	Yes
row_943	Atxn3	null	null	9319	-16.54673195	-0.122231446	Yes
row_944	Timm13	null	null	9328	-16.73526955	-0.12227383	Yes
row_945	Pgam5	null	null	9333	-16.93280029	-0.1219171	Yes
row_946	Tmem160	null	null	9334	-16.94737244	-0.121161245	Yes
row_947	Slc25a16	null	null	9351	-17.2355957	-0.12200187	Yes
row_948	Rps6kb1	null	null	9357	-17.37483025	-0.121744916	Yes
row_949	Nudt2	null	null	9361	-17.40889549	-0.1212884	Yes
row_950	Ndufaf3	null	null	9362	-17.49358749	-0.12053254	Yes
row_951	Timm22	null	null	9366	-17.53284073	-0.12007602	Yes
row_952	Pdss1	null	null	9370	-17.60705376	-0.11961951	Yes
row_953	Dhfr	null	null	9375	-17.68106461	-0.11926277	Yes
row_954	Dctpp1	null	null	9382	-17.84201622	-0.1191056	Yes
row_955	Akr1b10	null	null	9392	-18.05519485	-0.119247764	Yes
row_956	Slc25a36	null	null	9406	-18.37786102	-0.11978905	Yes
row_957	Nfu1	null	null	9413	-18.52360344	-0.11963188	Yes
row_958	Fdx1	null	null	9423	-18.87505341	-0.11977404	Yes
row_959	Acadsb	null	null	9429	-18.9678688	-0.11951709	Yes
row_960	Bola1	null	null	9434	-19.07062531	-0.119160354	Yes
row_961	Sdhc	null	null	9437	-19.20131493	-0.11860406	Yes
row_962	Diablo	null	null	9445	-19.28621101	-0.11854666	Yes
row_963	Metap1d	null	null	9450	-19.37894249	-0.11818992	Yes
row_964	Sirt3	null	null	9453	-19.47476387	-0.117633626	Yes
row_965	Cwc15	null	null	9459	-19.6007843	-0.11737667	Yes
row_966	Nudt13	null	null	9465	-19.71403694	-0.117119715	Yes
row_967	Surf1	null	null	9478	-19.95809746	-0.11756123	Yes
row_968	Cisd3	null	null	9498	-20.5403347	-0.1187012	Yes
row_969	Mrpl57	null	null	9499	-20.60731506	-0.117945336	Yes
row_970	Isca1	null	null	9505	-20.71168709	-0.11768838	Yes
row_971	Cnr1	null	null	9507	-20.77907181	-0.117032304	Yes
row_972	Pptc7	null	null	9514	-20.98081017	-0.11687513	Yes
row_973	Jtb	null	null	9516	-21.00700378	-0.11621905	Yes
row_974	Ndufs5	null	null	9517	-21.03370476	-0.1154632	Yes
row_975	Clybl	null	null	9521	-21.16021919	-0.11500668	Yes
row_976	Slc25a40	null	null	9535	-21.97784042	-0.11554797	Yes
row_977	Mrpl55	null	null	9547	-22.21569252	-0.11588969	Yes
row_978	Gcsh	null	null	9553	-22.3434124	-0.115632735	Yes
row_979	Ptpmt1	null	null	9559	-22.50498772	-0.11537578	Yes
row_980	Agps	null	null	9562	-22.54671097	-0.11481949	Yes
row_981	Rnf5	null	null	9571	-22.86269951	-0.114861876	Yes
row_982	Gfer	null	null	9582	-23.19923782	-0.11510382	Yes
row_983	Nr3c1	null	null	9595	-23.51283455	-0.115545325	Yes
row_984	Ndufaf1	null	null	9603	-23.62001228	-0.11548793	Yes
row_985	Rexo2	null	null	9606	-23.75908279	-0.114931636	Yes
row_986	Isoc2a	null	null	9607	-23.80036163	-0.114175774	Yes
row_987	Bag1	null	null	9612	-24.0206356	-0.11381904	Yes
row_988	Alkbh7	null	null	9620	-24.27889442	-0.11376165	Yes
row_989	Micu2	null	null	9631	-24.87324905	-0.11400359	Yes
row_990	Ndufb10	null	null	9634	-24.98407745	-0.11344729	Yes
row_991	Micu3	null	null	9642	-25.30237579	-0.1133899	Yes
row_992	Mrps16	null	null	9645	-25.3657341	-0.112833604	Yes
row_993	Prdx6	null	null	9649	-25.54658508	-0.11237709	Yes
row_994	Eci1	null	null	9651	-25.62137604	-0.11172101	Yes
row_995	Coq10b	null	null	9654	-25.65528297	-0.11116471	Yes
row_996	Sod1	null	null	9657	-25.82836533	-0.11060842	Yes
row_997	Glrx2	null	null	9684	-26.60131645	-0.11244685	Yes
row_998	Trnt1	null	null	9693	-26.91167068	-0.11248924	Yes
row_999	Nsun3	null	null	9696	-27.04464722	-0.11193294	Yes

row_1000	Ndufaf6	null	null	9704	-27.30491638	-0.11187555	Yes
row_1001	Abhd10	null	null	9707	-27.37851524	-0.11131925	Yes
row_1002	Slc25a4	null	null	9712	-27.59967422	-0.11096252	Yes
row_1003	Crebzf	null	null	9725	-28.0343895	-0.111404024	Yes
row_1004	Ado	null	null	9732	-28.18508911	-0.11124685	Yes
row_1005	Mobp	null	null	9739	-28.66893005	-0.11108967	Yes
row_1006	Chchd7	null	null	9755	-29.1933918	-0.11183052	Yes
row_1007	Mrpl39	null	null	9766	-29.67875099	-0.11207247	Yes
row_1008	Timmdc1	null	null	9777	-30.19294357	-0.11231442	Yes
row_1009	Tomm22	null	null	9796	-31.08649635	-0.11335461	Yes
row_1010	Tomm40l	null	null	9816	-31.93983078	-0.11449458	Yes
row_1011	Mtch2	null	null	9821	-32.15523911	-0.11413784	Yes
row_1012	Tyms	null	null	9830	-32.66235733	-0.11418023	Yes
row_1013	Col4a3bp	null	null	9837	-32.95975494	-0.11402305	Yes
row_1014	Creb1	null	null	9844	-33.38456726	-0.113865875	Yes
row_1015	Eny2	null	null	9848	-33.47211456	-0.11340936	Yes
row_1016	Prdx5	null	null	9859	-33.85455322	-0.113651305	Yes
row_1017	Isca2	null	null	9865	-34.08067703	-0.11339435	Yes
row_1018	Sfxn4	null	null	9872	-34.55498123	-0.11323718	Yes
row_1019	Chchd4	null	null	9880	-34.95825577	-0.11317978	Yes
row_1020	Hsdl2	null	null	9881	-35.11917496	-0.11242393	Yes
row_1021	Cox20	null	null	9887	-35.4295845	-0.11216697	Yes
row_1022	Ndufa11	null	null	9890	-35.52186966	-0.11161067	Yes
row_1023	Msrb2	null	null	9893	-35.61967087	-0.111054376	Yes
row_1024	Prkar2b	null	null	9897	-36.05644989	-0.11059786	Yes
row_1025	Kras	null	null	9901	-36.46847916	-0.110141344	Yes
row_1026	Sdhb	null	null	9906	-36.68238068	-0.1097846	Yes
row_1027	Atp5g3	null	null	9913	-37.01175308	-0.10962743	Yes
row_1028	Nthl1	null	null	9914	-37.04969406	-0.10887157	Yes
row_1029	Pstk	null	null	9924	-37.30030823	-0.109013736	Yes
row_1030	Uros	null	null	9927	-37.41860199	-0.108457446	Yes
row_1031	Rmdn2	null	null	9935	-37.97363281	-0.10840005	Yes
row_1032	Tfrc	null	null	9940	-38.28883362	-0.10804331	Yes
row_1033	Mrpl17	null	null	9948	-38.69493866	-0.10798592	Yes
row_1034	Mycbp	null	null	9953	-38.89450455	-0.10762918	Yes
row_1035	BC026585	null	null	9955	-39.0784111	-0.106973104	Yes
row_1036	Pnpt1	null	null	9957	-39.1652565	-0.10631703	Yes
row_1037	Abce1	null	null	9962	-39.57680511	-0.10596029	Yes
row_1038	Siva1	null	null	9966	-39.77045059	-0.105503775	Yes
row_1039	Mrpl46	null	null	9972	-40.10016251	-0.10524682	Yes
row_1040	G0s2	null	null	9981	-40.59399796	-0.105289206	Yes
row_1041	Dimt1	null	null	9985	-40.85116959	-0.10483269	Yes
row_1042	P2ry12	null	null	9989	-41.17295074	-0.104376175	Yes
row_1043	Coa3	null	null	9996	-41.46343231	-0.104219	Yes
row_1044	Immp2l	null	null	10002	-41.76685715	-0.10396204	Yes
row_1045	Mrpl23	null	null	10021	-43.24662781	-0.10500223	Yes
row_1046	Pin1	null	null	10022	-43.45586014	-0.10424637	Yes
row_1047	Tomm70a	null	null	10033	-44.77823257	-0.10448832	Yes
row_1048	Glrx5	null	null	10034	-44.81364441	-0.10373247	Yes
row_1049	Malsu1	null	null	10043	-45.1876297	-0.103774846	Yes
row_1050	Gtpbp10	null	null	10048	-45.46854782	-0.10341811	Yes
row_1051	Ndufaf4	null	null	10058	-46.54335403	-0.10356028	Yes
row_1052	Cox19	null	null	10072	-47.35327911	-0.10410157	Yes
row_1053	Pcbd2	null	null	10073	-47.47594833	-0.10334571	Yes
row_1054	Nubpl	null	null	10088	-48.5483551	-0.10398678	Yes
row_1055	Uqcrc2	null	null	10089	-48.59988403	-0.10323092	Yes
row_1056	Snca	null	null	10093	-48.96699905	-0.102774404	Yes
row_1057	Coq3	null	null	10099	-49.43915939	-0.10251745	Yes
row_1058	Lyrm1	null	null	10111	-50.40526581	-0.10285918	Yes
row_1059	Sdhaf1	null	null	10121	-50.80779266	-0.10300134	Yes
row_1060	Ak3	null	null	10129	-51.45952988	-0.10294395	Yes
row_1061	Cox4i1	null	null	10135	-51.9659996	-0.102686994	Yes
row_1062	Timm21	null	null	10146	-53.04618835	-0.10292894	Yes
row_1063	Nol7	null	null	10150	-53.28798294	-0.102472425	Yes
row_1064	Rps15a	null	null	10155	-53.77290344	-0.10211569	Yes
row_1065	Oxld1	null	null	10156	-53.78178024	-0.10135983	Yes
row_1066	mt-Tt	null	null	10162	-54.08966446	-0.10110287	Yes

row_1067	Mrpl15	null	null	10165	-54.14429855	-0.100546576	Yes
row_1068	Mrpl40	null	null	10166	-54.17938995	-0.09979072	Yes
row_1069	Mrps28	null	null	10168	-54.25280762	-0.09913464	Yes
row_1070	Mterf4	null	null	10187	-56.06287766	-0.10017483	Yes
row_1071	Mrpl21	null	null	10188	-56.15470123	-0.099418975	Yes
row_1072	Armc1	null	null	10192	-56.62251663	-0.098962456	Yes
row_1073	Tfam	null	null	10194	-56.79144669	-0.09830638	Yes
row_1074	Tusc3	null	null	10199	-57.31978989	-0.09794965	Yes
row_1075	Ssbp1	null	null	10206	-57.8459053	-0.09779247	Yes
row_1076	Fam213a	null	null	10213	-59.10402298	-0.09763529	Yes
row_1077	Hscb	null	null	10219	-59.78435135	-0.097378336	Yes
row_1078	Slc25a46	null	null	10224	-60.28212738	-0.0970216	Yes
row_1079	Ghitm	null	null	10226	-60.34692764	-0.09636553	Yes
row_1080	Dnajc15	null	null	10236	-61.1727829	-0.09650769	Yes
row_1081	Pet100	null	null	10246	-62.41042709	-0.096649855	Yes
row_1082	Chchd6	null	null	10254	-63.19470596	-0.09659246	Yes
row_1083	Mut	null	null	10271	-65.41750336	-0.09743309	Yes
row_1084	Gdap1	null	null	10275	-66.26888275	-0.09697658	Yes
row_1085	Cmc1	null	null	10282	-67.03113556	-0.0968194	Yes
row_1086	Fmc1	null	null	10295	-68.77264404	-0.09726091	Yes
row_1087	Mat2b	null	null	10303	-69.92798615	-0.097203515	Yes
row_1088	Ndufb7	null	null	10306	-70.14627838	-0.09664722	Yes
row_1089	mt-Nd5	null	null	10320	-71.7754364	-0.09718851	Yes
row_1090	Ptrh2	null	null	10323	-72.17381287	-0.09663221	Yes
row_1091	Atp5c1	null	null	10327	-72.67047882	-0.09617569	Yes
row_1092	Ccdc58	null	null	10333	-73.21050262	-0.09591874	Yes
row_1093	Aurkaip1	null	null	10335	-73.55129242	-0.09526266	Yes
row_1094	Bok	null	null	10340	-74.38480377	-0.09490593	Yes
row_1095	Ndufaf2	null	null	10351	-75.30464172	-0.09514787	Yes
row_1096	Ireb2	null	null	10360	-75.9850769	-0.09519026	Yes
row_1097	Gatm	null	null	10370	-77.37644958	-0.09533242	Yes
row_1098	Mrpl20	null	null	10377	-78.18450928	-0.09517525	Yes
row_1099	Oxr1	null	null	10379	-78.50544739	-0.09451917	Yes
row_1100	Rpl35a	null	null	10381	-78.56951141	-0.09386309	Yes
row_1101	Rrm2b	null	null	10384	-78.67315674	-0.093306795	Yes
row_1102	Rpl10a	null	null	10385	-78.81176758	-0.09255094	Yes
row_1103	Tmem70	null	null	10386	-78.95426178	-0.09179508	Yes
row_1104	Atp5k	null	null	10393	-80.08720398	-0.09163791	Yes
row_1105	Atad1	null	null	10402	-80.63222504	-0.091680296	Yes
row_1106	Atp5g1	null	null	10403	-80.78824615	-0.090924434	Yes
row_1107	Iscu	null	null	10406	-81.19332886	-0.09036814	Yes
row_1108	Rdh14	null	null	10407	-81.45159912	-0.08961228	Yes
row_1109	Ndufa8	null	null	10411	-81.88077545	-0.08915576	Yes
row_1110	Gpx1	null	null	10421	-83.44191742	-0.08929793	Yes
row_1111	Hebp2	null	null	10422	-83.63102722	-0.088542074	Yes
row_1112	Ndufa13	null	null	10427	-84.91810608	-0.08818533	Yes
row_1113	Chchd1	null	null	10430	-85.17641449	-0.087629035	Yes
row_1114	P2ry1	null	null	10438	-87.35630035	-0.08757164	Yes
row_1115	Hadhb	null	null	10440	-87.66394806	-0.08691557	Yes
row_1116	1700123O20Rik	null	null	10448	-88.73156738	-0.086858176	Yes
row_1117	mt-Tp	null	null	10451	-89.54573059	-0.08630188	Yes
row_1118	Mpv17l	null	null	10452	-89.56101227	-0.08554602	Yes
row_1119	Pdp1	null	null	10457	-90.84617615	-0.08518928	Yes
row_1120	Nadk2	null	null	10458	-90.9777832	-0.08443342	Yes
row_1121	Mrpl32	null	null	10459	-91.17756653	-0.08367757	Yes
row_1122	Mrpl11	null	null	10461	-91.59705353	-0.083021484	Yes
row_1123	Glrx	null	null	10466	-93.67127228	-0.08266475	Yes
row_1124	Mrpl35	null	null	10471	-93.95879364	-0.08230802	Yes
row_1125	Dnajc19	null	null	10476	-94.38348389	-0.08195128	Yes
row_1126	Cox7a2l	null	null	10482	-94.97776794	-0.08169433	Yes
row_1127	Mrps31	null	null	10487	-95.94171143	-0.081337586	Yes
row_1128	Sccpdh	null	null	10504	-98.46676636	-0.08217822	Yes
row_1129	Ndufaf5	null	null	10510	-99.18267059	-0.081921265	Yes
row_1130	Mrpl50	null	null	10512	-99.57808685	-0.08126519	Yes
row_1131	Echs1	null	null	10532	-102.5301971	-0.08240516	Yes
row_1132	Mrps15	null	null	10533	-102.5806046	-0.081649296	Yes
row_1133	Bloc1s2	null	null	10535	-102.9868927	-0.08099322	Yes

row_1134	Acss3	null	null	10538	-103.3729324	-0.08043692	Yes
row_1135	Tomm7	null	null	10540	-103.9304123	-0.07978085	Yes
row_1136	Mrpl41	null	null	10549	-105.0257263	-0.07982323	Yes
row_1137	Minos1	null	null	10556	-106.0631256	-0.079666056	Yes
row_1138	Cebpzos	null	null	10558	-106.1506195	-0.07900998	Yes
row_1139	Pfdn2	null	null	10559	-106.2244415	-0.078254126	Yes
row_1140	Mrps33	null	null	10560	-106.3042831	-0.077498265	Yes
row_1141	Timm9	null	null	10569	-108.0822144	-0.07754065	Yes
row_1142	Timm10	null	null	10571	-108.9081802	-0.076884575	Yes
row_1143	Yme1l1	null	null	10575	-111.4082108	-0.076428056	Yes
row_1144	Ndufb6	null	null	10578	-112.1855392	-0.07587176	Yes
row_1145	Mrps36	null	null	10582	-113.0261688	-0.075415246	Yes
row_1146	Ndufb9	null	null	10586	-114.2367783	-0.07495873	Yes
row_1147	Vamp8	null	null	10591	-115.3288116	-0.07460199	Yes
row_1148	Cyb5a	null	null	10593	-115.4785843	-0.07394591	Yes
row_1149	Sdhaf4	null	null	10599	-117.0391159	-0.07368896	Yes
row_1150	Mrpl27	null	null	10604	-117.6448135	-0.07333222	Yes
row_1151	Gng5	null	null	10605	-117.6814728	-0.07257637	Yes
row_1152	Tbc1d15	null	null	10609	-118.394722	-0.07211985	Yes
row_1153	Triap1	null	null	10613	-119.2129974	-0.07166333	Yes
row_1154	Lyrm7	null	null	10618	-121.4991531	-0.07130659	Yes
row_1155	Ndfip2	null	null	10621	-122.4475784	-0.070750296	Yes
row_1156	Mrpl12	null	null	10623	-122.7796249	-0.07009422	Yes
row_1157	Gstk1	null	null	10626	-122.9423904	-0.06953792	Yes
row_1158	Sdhaf3	null	null	10630	-125.882637	-0.06908141	Yes
row_1159	Mpc2	null	null	10642	-128.825943	-0.06942313	Yes
row_1160	Mterf2	null	null	10645	-129.4467926	-0.068866834	Yes
row_1161	Bola3	null	null	10646	-129.4609222	-0.06811098	Yes
row_1162	Mthfsl	null	null	10655	-130.8339386	-0.06815337	Yes
row_1163	Idh1	null	null	10659	-132.4538422	-0.06769685	Yes
row_1164	Mrps10	null	null	10666	-133.4852448	-0.06753968	Yes
row_1165	Rmdn3	null	null	10677	-136.1708984	-0.06778162	Yes
row_1166	Ngdn	null	null	10690	-139.246582	-0.068223126	Yes
row_1167	Sdhd	null	null	10691	-139.5778198	-0.06746727	Yes
row_1168	Sri	null	null	10692	-139.7857971	-0.06671141	Yes
row_1169	Mrps14	null	null	10694	-141.0014954	-0.066055335	Yes
row_1170	Ech1	null	null	10712	-147.0210266	-0.06699575	Yes
row_1171	Fis1	null	null	10717	-148.0871277	-0.06663901	Yes
row_1172	Rpl34	null	null	10718	-148.569458	-0.06588315	Yes
row_1173	Fam136a	null	null	10726	-151.6855164	-0.06582576	Yes
row_1174	Acat1	null	null	10729	-152.2902374	-0.06526946	Yes
row_1175	Nt5c3	null	null	10730	-152.3727417	-0.0645136	Yes
row_1176	Ndufa5	null	null	10731	-152.454422	-0.06375775	Yes
row_1177	Cycs	null	null	10733	-152.6729126	-0.063101664	Yes
row_1178	Decr1	null	null	10734	-153.1964111	-0.06234581	Yes
row_1179	Acot13	null	null	10744	-156.6663666	-0.062487975	Yes
row_1180	Adh5	null	null	10750	-159.9565277	-0.06223102	Yes
row_1181	Coq7	null	null	10752	-159.9845123	-0.061574943	Yes
row_1182	Uqcrfs1	null	null	10755	-160.5681915	-0.061018646	Yes
row_1183	Uqcr11	null	null	10763	-162.8999634	-0.06096125	Yes
row_1184	Trmt10c	null	null	10778	-169.5286102	-0.06160232	Yes
row_1185	Mrpl24	null	null	10786	-171.3456726	-0.061544925	Yes
row_1186	Pdhb	null	null	10788	-173.1611633	-0.06088885	Yes
row_1187	Bbox1	null	null	10791	-175.2691956	-0.06033255	Yes
row_1188	Bnip3l	null	null	10792	-175.5288544	-0.059576694	Yes
row_1189	Mrpl18	null	null	10794	-175.7474976	-0.058920614	Yes
row_1190	Rida	null	null	10802	-178.0277252	-0.058863223	Yes
row_1191	Agpat5	null	null	10805	-178.5522461	-0.058306925	Yes
row_1192	Ndufs6	null	null	10812	-181.0307159	-0.05814975	Yes
row_1193	Mtrf1l	null	null	10827	-189.5698547	-0.058790818	Yes
row_1194	Grpel1	null	null	10828	-190.0778809	-0.05803496	Yes
row_1195	Lyrm2	null	null	10834	-193.1143646	-0.057778005	Yes
row_1196	Nudt19	null	null	10843	-197.8856201	-0.05782039	Yes
row_1197	Ociad2	null	null	10844	-198.9339142	-0.057064533	Yes
row_1198	Lyrm4	null	null	10866	-212.6182556	-0.058404066	Yes
row_1199	Tmem126a	null	null	10879	-219.2784119	-0.058845572	Yes
row_1200	Ccdc90b	null	null	10881	-219.7425537	-0.058189496	Yes

row_1201	Mrpl54	null	null	10882	-220.9447937	-0.05743364	Yes
row_1202	Uqcrb	null	null	10884	-221.0401459	-0.05677756	Yes
row_1203	1110008F13Rik	null	null	10890	-225.6994019	-0.056520604	Yes
row_1204	Vdac3	null	null	10895	-227.7704315	-0.05616387	Yes
row_1205	Mrpl47	null	null	10898	-228.3879852	-0.055607572	Yes
row_1206	Ndufb4	null	null	10900	-230.1529541	-0.054951496	Yes
row_1207	Qdpr	null	null	10913	-240.8872681	-0.055393003	Yes
row_1208	Etfa	null	null	10914	-241.0535583	-0.054637145	Yes
row_1209	Mrps21	null	null	10920	-245.4321136	-0.05438019	Yes
row_1210	mt-Cytb	null	null	10927	-249.171875	-0.054223016	Yes
row_1211	Romo1	null	null	10929	-251.1148987	-0.053566936	Yes
row_1212	Tomm5	null	null	10933	-252.4406128	-0.05311042	Yes
row_1213	Tmem126b	null	null	10940	-255.2789154	-0.052953247	Yes
row_1214	Txn1	null	null	10947	-262.6124268	-0.05279607	Yes
row_1215	Fastkd2	null	null	10952	-266.7426758	-0.052439336	Yes
row_1216	Dld	null	null	10957	-270.6190186	-0.0520826	Yes
row_1217	Mff	null	null	10958	-272.1513977	-0.05132674	Yes
row_1218	Atp5j	null	null	10962	-273.1491089	-0.050870225	Yes
row_1219	Rps14	null	null	10969	-279.6862183	-0.050713047	Yes
row_1220	Smim4	null	null	10970	-280.773468	-0.04995719	Yes
row_1221	2410015M20Rik	null	null	10972	-282.0343323	-0.049301114	Yes
row_1222	Emc2	null	null	10973	-282.3363037	-0.048545256	Yes
row_1223	Ndufs8	null	null	10979	-287.3091125	-0.0482883	Yes
row_1224	Vps35	null	null	10982	-288.8184204	-0.047732003	Yes
row_1225	Uqcrh	null	null	10985	-289.26828	-0.047175705	Yes
row_1226	Ndufa12	null	null	10988	-291.8304443	-0.046619408	Yes
row_1227	C330018D20Rik	null	null	10989	-292.0364685	-0.04586355	Yes
row_1228	Ndufab1	null	null	10992	-294.145874	-0.045307253	Yes
row_1229	Ndufa6	null	null	10994	-297.1447754	-0.044651177	Yes
row_1230	mt-Co1	null	null	10995	-297.6434631	-0.04389532	Yes
row_1231	Atp5l	null	null	11001	-305.8949585	-0.043638363	Yes
row_1232	Ndufv3	null	null	11008	-310.8027344	-0.04348119	Yes
row_1233	Mrpl42	null	null	11017	-321.1486816	-0.043523576	Yes
row_1234	Atp5e	null	null	11018	-323.6139526	-0.042767715	Yes
row_1235	Rab29	null	null	11020	-324.2150879	-0.04211164	Yes
row_1236	Ndufb3	null	null	11023	-326.5795898	-0.04155534	Yes
row_1237	Ndufc1	null	null	11033	-334.5463867	-0.04169751	Yes
row_1238	Cbr4	null	null	11041	-340.0866089	-0.041640114	Yes
row_1239	Mrpl22	null	null	11042	-341.1478577	-0.040884256	Yes
row_1240	Hspe1	null	null	11046	-346.5660095	-0.04042774	Yes
row_1241	Hddc2	null	null	11050	-350.5193481	-0.039971225	Yes
row_1242	Uqcrq	null	null	11051	-351.4783325	-0.039215364	Yes
row_1243	Slirp	null	null	11053	-354.5403137	-0.038559288	Yes
row_1244	Cox6b1	null	null	11058	-360.0204468	-0.03820255	Yes
row_1245	Ndufa7	null	null	11061	-366.0018311	-0.037646256	Yes
row_1246	Cox6c	null	null	11064	-368.4577637	-0.03708996	Yes
row_1247	Fdx1l	null	null	11067	-372.1758423	-0.03653366	Yes
row_1248	Atp5j2	null	null	11069	-372.3390808	-0.035877585	Yes
row_1249	Mcee	null	null	11073	-375.6546326	-0.035421066	Yes
row_1250	Them4	null	null	11079	-381.8096924	-0.03516411	Yes
row_1251	Hibch	null	null	11081	-382.0937195	-0.034508035	Yes
row_1252	Ndufa2	null	null	11084	-385.3541565	-0.033951737	Yes
row_1253	Scp2	null	null	11099	-403.3277893	-0.034592807	Yes
row_1254	Prdx1	null	null	11101	-405.9992371	-0.033936728	Yes
row_1255	mt-Rnr2	null	null	11107	-413.6788635	-0.033679772	Yes
row_1256	Ndufa4	null	null	11108	-414.8104553	-0.032923914	Yes
row_1257	Acyp2	null	null	11115	-423.4879456	-0.03276674	Yes
row_1258	Atp5h	null	null	11118	-425.3863831	-0.032210443	Yes
row_1259	Pts	null	null	11124	-431.2923889	-0.031953488	Yes
row_1260	Cox5b	null	null	11126	-433.5984192	-0.03129741	Yes
row_1261	Chchd3	null	null	11127	-435.0106812	-0.030541552	Yes
row_1262	Cox6a1	null	null	11128	-436.5970764	-0.029785695	Yes
row_1263	Ndufb5	null	null	11135	-453.8414307	-0.029628519	Yes
row_1264	Crls1	null	null	11136	-454.217926	-0.028872661	Yes
row_1265	Cox7c	null	null	11139	-462.2252197	-0.028316366	Yes
row_1266	Atpif1	null	null	11141	-467.4630127	-0.027660288	Yes
row_1267	Ndufc2	null	null	11145	-476.1025696	-0.02720377	Yes

row_1268	Mrpl30	null	null	11147	-485.053894	-0.026547693	Yes
row_1269	Ndufs4	null	null	11154	-502.0514526	-0.026390519	Yes
row_1270	mt-Rnr1	null	null	11155	-509.6646729	-0.025634661	Yes
row_1271	Mrpl13	null	null	11158	-511.0132446	-0.025078364	Yes
row_1272	Nit2	null	null	11165	-533.8369141	-0.024921188	Yes
row_1273	Coa6	null	null	11167	-535.1019897	-0.02426511	Yes
row_1274	Cox7a2	null	null	11172	-542.3001709	-0.023908375	Yes
row_1275	Mrpl52	null	null	11173	-546.9841919	-0.023152517	Yes
row_1276	Mgst1	null	null	11174	-550.0083008	-0.02239666	Yes
row_1277	Idi1	null	null	11177	-568.5610962	-0.021840362	Yes
row_1278	Timm8b	null	null	11179	-572.4307861	-0.021184284	Yes
row_1279	Cox8a	null	null	11189	-609.3577271	-0.02132645	Yes
row_1280	Mrpl48	null	null	11190	-613.0897827	-0.020570593	Yes
row_1281	Sugct	null	null	11194	-623.5093384	-0.020114077	Yes
row_1282	Fam162a	null	null	11195	-625.0778809	-0.01935822	Yes
row_1283	Coq2	null	null	11196	-627.296814	-0.018602362	Yes
row_1284	Uqcc2	null	null	11198	-650.5671387	-0.017946284	Yes
row_1285	Echdc2	null	null	11199	-651.3895264	-0.017190427	Yes
row_1286	Fth1	null	null	11202	-659.2412109	-0.016634129	Yes
row_1287	Usmg5	null	null	11203	-659.8202515	-0.015878271	Yes
row_1288	Mtx2	null	null	11210	-702.46875	-0.015721096	Yes
row_1289	2010107E04Rik	null	null	11213	-704.3325195	-0.015164799	Yes
row_1290	Sucla2	null	null	11214	-710.5256958	-0.014408941	Yes
row_1291	Gpx4	null	null	11217	-724.1456299	-0.013852644	Yes
row_1292	Chchd2	null	null	11218	-726.7298584	-0.013096786	Yes
row_1293	Cox14	null	null	11219	-729.1559448	-0.012340928	Yes
row_1294	Echdc1	null	null	11223	-747.9562988	-0.011884412	Yes
row_1295	Mpc1	null	null	11226	-788.2207031	-0.011328115	Yes
row_1296	Mdh1	null	null	11230	-810.9002075	-0.010871599	Yes
row_1297	Mrpl36	null	null	11231	-811.0979004	-0.010115741	Yes
row_1298	Mrpl1	null	null	11233	-831.3317261	-0.009459663	Yes
row_1299	Mterf3	null	null	11235	-833.3121338	-0.008803586	Yes
row_1300	Higd1a	null	null	11236	-843.5780029	-0.008047728	Yes
row_1301	Dynll1	null	null	11241	-873.329834	-0.007690992	Yes
row_1302	Bnip3	null	null	11243	-876.8321533	-0.007034915	Yes
row_1303	Ndufa3	null	null	11245	-892.0710449	-0.006378837	Yes
row_1304	Uqcr10	null	null	11250	-926.0384521	-0.006022101	Yes
row_1305	Tmem256	null	null	11254	-955.6976318	-0.005565585	Yes
row_1306	Prdx3	null	null	11255	-956.8841553	-0.004809727	Yes
row_1307	Ndufv2	null	null	11257	-971.1478271	-0.004153649	Yes
row_1308	Pam16	null	null	11258	-974.7127686	-0.003397791	Yes
row_1309	Dbi	null	null	11262	-1083.3573	-0.002941275	Yes
row_1310	mt-Nd6	null	null	11264	-1101.637085	-0.002285197	Yes
row_1311	Hint2	null	null	11273	-1291.182373	-0.002327583	Yes
row_1312	Atp5f1	null	null	11281	-1457.118286	-0.002270189	Yes
row_1313	Atp5s	null	null	11282	-1463.586914	-0.001514331	Yes
row_1314	Tefm	null	null	11285	-1530.48584	-9.58E-04	Yes
row_1315	Yars2	null	null	11296	-1858.709961	-0.001199981	Yes
row_1316	Mtrf1	null	null	11301	-1992.6427	-8.43E-04	Yes
row_1317	Pfdn4	null	null	11306	-2174.711182	-4.87E-04	Yes
row_1318	Immp1l	null	null	11309	-2341.505127	6.98E-05	Yes
row_1319	Mthfd2l	null	null	11312	-2691.698242	6.26E-04	Yes
row_1320	Mrps22	null	null	11326	-6304.582031	8.48E-05	Yes
row_1321	Ankrd37	null	null	11338	-21064.36914	-2.57E-04	Yes
row_1322	Dtymk	null	null	11341	-107098.7656	2.99E-04	Yes

Humanised mutant FUS drives progressive motor neuron degeneration without aggregation in 'FUSDelta14' knockin mice
Devoy et al. 2017
Supplementary Table 8 Gene Set Enrichment Analysis (GSEA) **Mitochondrial respiratory chain** - list of genes dysregulated at 12 months of age in I

NAME	PROBE	GENE SYMBOL	GENE_TITLE	RANK IN GENE LIST	RANK METRIC SCORE	RUNNING ES	CORE ENRICHMENT
row_0	Sdha	null	null	3649	4.021390915	-0.30637178	No
row_1	Ndufv1	null	null	4416	2.484201193	-0.35729432	No
row_2	Uqcrc1	null	null	4584	2.235046864	-0.35514227	No
row_3	Ndufa10	null	null	5737	1.191801667	-0.4402665	No
row_4	Ndufs2	null	null	5952	1.079359531	-0.4422789	No
row_5	Ndufs1	null	null	5965	1.070915699	-0.426393	No
row_6	Foxred1	null	null	6175	-1.02528739	-0.42796236	No
row_7	Ndufs3	null	null	6847	-1.457847953	-0.4704674	No
row_8	Pmpcb	null	null	8028	-3.731791258	-0.55807257	Yes
row_9	Cyc1	null	null	8053	-3.818825006	-0.5432499	Yes
row_10	Uqcc3	null	null	8148	-4.184010506	-0.5346297	Yes
row_11	Ndufs7	null	null	8482	-6.047410011	-0.54718614	Yes
row_12	Ndufa9	null	null	8773	-8.412945747	-0.5559325	Yes
row_13	Cox5a	null	null	8790	-8.647891998	-0.54040104	Yes
row_14	Cox7a1	null	null	9050	-11.77324963	-0.54640067	Yes
row_15	Park7	null	null	9289	-15.90094566	-0.5505396	Yes
row_16	Sdhc	null	null	9437	-19.20131493	-0.5466154	Yes
row_17	Ndufs5	null	null	9517	-21.03370476	-0.5366661	Yes
row_18	Ndufb10	null	null	9634	-24.98407745	-0.5299952	Yes
row_19	Ndufa11	null	null	9890	-35.52186966	-0.5356404	Yes
row_20	Sdhb	null	null	9906	-36.68238068	-0.5200203	Yes
row_21	Uqcrc2	null	null	10089	-48.59988403	-0.51919734	Yes
row_22	Cox4i1	null	null	10135	-51.9659996	-0.5062354	Yes
row_23	Ndufb7	null	null	10306	-70.14627838	-0.5043492	Yes
row_24	Ndufa8	null	null	10411	-81.88077545	-0.496615	Yes
row_25	Ndufa13	null	null	10427	-84.91810608	-0.4809949	Yes
row_26	Cox7a2l	null	null	10482	-94.97776794	-0.46883047	Yes
row_27	Ndufb6	null	null	10578	-112.1855392	-0.4602988	Yes
row_28	Ndufb9	null	null	10586	-114.2367783	-0.4439699	Yes
row_29	Sdhd	null	null	10691	-139.5778198	-0.4362357	Yes
row_30	Ndufa5	null	null	10731	-152.454422	-0.42274216	Yes
row_31	Uqcrfs1	null	null	10755	-160.5681915	-0.40783092	Yes
row_32	Ndufs6	null	null	10812	-181.0307159	-0.39584368	Yes
row_33	Uqcrb	null	null	10884	-221.0401459	-0.3851855	Yes
row_34	Ndufb4	null	null	10900	-230.1529541	-0.36956543	Yes
row_35	Ndufs8	null	null	10979	-287.3091125	-0.3595275	Yes
row_36	Uqcrh	null	null	10985	-289.26828	-0.34302136	Yes
row_37	Ndufa12	null	null	10988	-291.8304443	-0.32624942	Yes
row_38	Ndufab1	null	null	10992	-294.145874	-0.30956608	Yes
row_39	Ndufa6	null	null	10994	-297.1447754	-0.29270554	Yes
row_40	mt-Co1	null	null	10995	-297.6434631	-0.2757564	Yes
row_41	Ndufv3	null	null	11008	-310.8027344	-0.2598705	Yes
row_42	Ndufb3	null	null	11023	-326.5795898	-0.24416181	Yes
row_43	Ndufc1	null	null	11033	-334.5463867	-0.22801012	Yes
row_44	Uqcrq	null	null	11051	-351.4783325	-0.21256725	Yes
row_45	Ndufa7	null	null	11061	-366.0018311	-0.19641556	Yes
row_46	Ndufa2	null	null	11084	-385.3541565	-0.18141572	Yes
row_47	Ndufa4	null	null	11108	-414.8104553	-0.16650449	Yes
row_48	Cox5b	null	null	11126	-433.5984192	-0.15106162	Yes
row_49	Cox6a1	null	null	11128	-436.5970764	-0.13420108	Yes
row_50	Ndufb5	null	null	11135	-453.8414307	-0.11778356	Yes
row_51	Cox7c	null	null	11139	-462.2252197	-0.10110022	Yes
row_52	Ndufc2	null	null	11145	-476.1025696	-0.08459409	Yes
row_53	Ndufs4	null	null	11154	-502.0514526	-0.06835379	Yes
row_54	Cox7a2	null	null	11172	-542.3001709	-0.052910924	Yes
row_55	Cox8a	null	null	11189	-609.3577271	-0.03737946	Yes
row_56	Ndufa3	null	null	11245	-892.0710449	-0.025303598	Yes
row_57	Uqcr10	null	null	11250	-926.0384521	-0.008708868	Yes
row_58	Ndufv2	null	null	11257	-971.1478271	0.007708653	Yes

FUS Delta14 spinal cord.

Humanised mutant FUS drives progressive motor neuron degeneration without aggregation in 'FUSDelta14' knockin mice
Devoy et al. 2017

Supplementary Table 9 Gene Set Enrichment Analysis (GSEA) **Proteasome Core Complex** - list of genes dysregulated at 12 months of age in FUS

NAME	PROBE	GENE SYMBOL	GENE_TITLE	RANK IN GENE LIST	RANK METRIC SCORE	RUNNING ES	CORE ENRICHMENT
row_0	Alad	null	null	4457	2.415877342	-0.33462632	No
row_1	Psmb8	null	null	7485	-2.314165592	-0.5430168	No
row_2	Psme4	null	null	8867	-9.392008781	-0.60610354	Yes
row_3	Psma7	null	null	8950	-10.50782394	-0.55451876	Yes
row_4	Psmb10	null	null	9104	-12.60319042	-0.5092016	Yes
row_5	Psmb2	null	null	9335	-16.95043755	-0.4706817	Yes
row_6	Psmb6	null	null	9773	-30.09322166	-0.45043516	Yes
row_7	Psmb4	null	null	9833	-32.73668671	-0.39681995	Yes
row_8	Psma4	null	null	10624	-122.8734818	-0.40773514	Yes
row_9	Psmb3	null	null	10701	-143.6298981	-0.35562065	Yes
row_10	Psma2	null	null	10862	-211.6173859	-0.3109214	Yes
row_11	Psma3	null	null	11048	-347.6372681	-0.2684291	Yes
row_12	Psma5	null	null	11068	-372.2513428	-0.21128282	Yes
row_13	Psma6	null	null	11080	-381.8269043	-0.15343033	Yes
row_14	Psmb1	null	null	11133	-450.9679565	-0.0991972	Yes
row_15	Psmb7	null	null	11178	-569.8171997	-0.04425785	Yes
row_16	Psma1	null	null	11253	-945.2886353	0.008033194	Yes

Delta14 spinal cord.

Humanised mutant FUS drives progressive motor neuron degeneration without aggregation in 'FUSDelta14' knockin mice
Devoy et al. 201

Supplementary Table 10 Gene Set Enrichment Analysis (GSEA) **Proteasome core complex - alpha subunit complex** - list of genes dysregulate
at 12 months of age in FUS Delta14 spinal cord.

NAME	PROBE	GENE SYMBOL	GENE_TITLE	RANK IN GENE LIST	RANK METRIC SCORE	RUNNING ES	CORE ENRICHMENT
row_0	Psma7	null	null	8950	-10.50782394	-0.6465237	No
row_1	Psma4	null	null	10624	-122.8734818	-0.6512234	Yes
row_2	Psma2	null	null	10862	-211.6173859	-0.52926946	Yes
row_3	Psma3	null	null	11048	-347.6372681	-0.4027291	Yes
row_4	Psma5	null	null	11068	-372.2513428	-0.2615477	Yes
row_5	Psma6	null	null	11080	-381.8269043	-0.119660765	Yes
row_6	Psma1	null	null	11253	-945.2886353	0.008026158	Yes

ed
