# Supplementary material for: Humanized mutant FUS drives progressive motor neuron degeneration without aggregation in ‘FUSDelta14’ knockin mice
Source: Brain. 2017 Oct 7;140(11):2797–805. doi: 10.1093/brain/awx248 (PMC5841203; doi:10.1093/brain/awx248)
Supplement: Supplementary Materials and Methods [file devoy_et_al_supplementary_materials_and_methods_awx248.pdf]

Humanised mutant FUS drives progressive motor neuron degeneration without aggregation in ‘FUSDelta14’ knockin mice

Supplementary Materials and Methods

Generation and maintenance of FUS Delta14 mice

The new strain is designated B6N;B6J-Fus$^{tm1Emcf/H}$, referred to here as FUS Delta14.

FUS Delta14 mice were generated by homologous recombination in the C57BL6/N mouse embryonic stem cell (ESC) line JM8-F6 (Pettitt, Liang et al. 2009) using standard procedures. Briefly, a construct of 8kb with FRT-flanked Neo cassette, introduced an A to G splice mutation at g.13845 and humanised the coding sequence of exon 15 through the following sequence changes: g.14230 C>T, g.14232 A>T, g.14234C>G, g.14260 A>G and g.14266 ATTA insertion. Correct clones were initially identified through long-range PCR and copy number qPCR and validated by RT-PCR and western blot for the truncated RNA/protein product, followed by DNA sequence verification. Mice were generated by injection of modified ESCs into B6(Cg)-*Tyr*$^{c\text{-}2J/J}$ (B6-albino) donor embryos, with the resultant chimeric male offspring crossed to B6-albino females to obtain germline transmission (GLT). GLT was confirmed by copy number qPCR. The FRT-flanked Neo cassette was removed by crossing to the Flpo-expression line (B6(C3)-Tg(Pgk1-FLPo)10Sykr/J) which is maintained on the C57BL6/J background. The Neo-negative line then was backcrossed for a minimum of three generations onto the C57BL6/J background before experimental cohorts were bred. Genotyping was performed by standard PCR using the following primers:
001 GGTCAGAGATCATGAGGAAATCAGG
002 GAGTTCTACAAAGTGAGTTCCAGGACAG
003 CAGGAGCCAGGCTAATTAATAC
PCR product formed: Wild-type allele (001+002): 659 bp; Mutant allele (001+003): 258 bp. This line has been submitted to the European Mouse Mutant Archive (EMMA, EM:11106).

Generation of FUS Delta14 antibody

Antibodies specific to FUS Delta14 were generated by Everest Biotech Ltd. In brief, two goats were immunised with the 15 residue peptide MGVSTDRIAGRGRIN. The goat sera were affinity purified and the resultant polyclonal antibodies validated by ELISA before being optimised for immunoblot and immunocytochemistry.

Behavioural analysis

All longitudinal behavioural analysis was carried out on a cohort of 60 mice, constituting 15 mice per sex per genotype at the start of the study. This cohort was also used for the Kaplan-Meier survival analysis.

Wildtype and heterozygous FUS Delta14 littermates were assessed for motor function using Locotronic equipment as per the manufacturer's instructions (Intellibio, Nancy, France). The locotronic is a horizontal ladder that counts errors made when paws slip from the rungs or miss rungs entirely, as mice move across it. Locotronic analysis was performed at 2, 6 and 15 months of age. Statistical analysis was only carried out on mice with readings from all time-points. Cohort size was as follows: 11 wildtype males, 11 wildtype females, 9 FUS Delta14 males and 11 FUS Delta14 females.

Wildtype and heterozygous FUS Delta14 littermates had gait assessed using a treadmill and Treadscan video tracking system (Cleversys, Virginia, US). Gait analysis was performed at 6 and 18 months of age. Statistical analysis was only carried out on mice with readings from both time-points. Cohort size was as follows: 10 wildtype males, 10 wildtype females, 7 FUS Delta14 males and 12 FUS Delta14 females.

Tissue preparation

Tissues for molecular analysis (RNA and protein studies) were prepared via cervical dislocation of mice, dissection and snap freezing in liquid nitrogen for storage at -80 ºC until required. Tissue was taken at 3 and 12 months of age. Cohort size was 10 wildtype (5 male and 5 female) and 10 FUS Delta14 (5 male and 5 female) for each timepoint.

Tissue for pathological analysis were prepared by terminal anesthesia of mice, cardiac perfusion of fixative (4% PFA in PBS, pH 7.4) followed by immersion fixation of whole cadaver for 24 hours at 4 ºC. Tissues were then dissected and processed into paraffin wax in a Leica ASP300S tissue processor and embedded in fresh wax using a Leica embedding centre (EG1150H) and cold plate. Tissue was taken at 3, 12 and 18 months of age. Cohort size was 10 wildtype (5 male and 5 female) and 10 FUS Delta14 (5 male and 5 female) at 3 and 12 months, and 6 wildtype (3 male and 3 female) and 6 FUS Delta14 (3 male and 3 female) at 18 months of age.

Immunoblotting

Snap frozen spinal cord tissue from 3 and 12 month old wildtype and FUS Delta14 littermates was homogenised in CellLytic MT buffer (Sigma) with protease and phosphatase inhibitors (Sigma and Roche Diagnostic, respectively) using a conical-tipped 1ml Teflon-coated homogenizer at 4°C. Homogenates were centrifuged at 17,000 xg for 15 minutes and the soluble fraction removed. 20 μg of the soluble fraction was resolved by SDS–PAGE (NUPAGE system, Invitrogen) and transferred to low fluorescent PVDF (PVDF-FL) membranes (Millipore). Blots were incubated with primary and secondary antibodies at the following concentrations: anti-N-terminal FUS (Santa Cruz), 1:2000; anti-C-terminal FUS (Novus Biologicals), 1:5000; anti-frameshift FUS, 1:1000, anti-β-actin (Sigma), 1:10000, anti-mouse-HRP (DAKO), 1:10000, anti-rabbit-HRP (CST) 1:2000, anti-goat-HRP (Jackson Immunoresearch) 1:20000. Protein was visualized using SuperSignal West Pico Chemiluminescent Substrate (ThermoFisher) with Amersham Hyperfilm-ECL (GE healthcare) and film developed using a SRX-101A xograph developer machine (Konica Minolta). Quantified western blots were carried out as above but with fluorescent development for visualisation using a LiCor Odessey scanner and normalized to total protein using REVERT total protein stain (LiCor); with anti-N-terminal FUS (Santa Cruz), 1:2000; anti-total OXPHOS cocktail (Abcam), 1:2000; anti-Psma5 (CST), 1:2000, anti-Psmb5 (CST), 1:2000; anti-β-actin (Sigma), 1:10000; anti-mouse and anti-rabbit-IRDye 800CW (LiCor), 1:10,000. One exception was anti-RPL26 (CST), 1:200, which was visualized using SuperSignal West Pico Chemiluminescent Substrate (ThermoFisher) with a BioRad ChemiDoc Touch Scanner and normalized to total protein using REVERT total protein stain (LiCor).

Soluble and Insoluble Fractionation

Snap frozen spinal cord tissue from 3 month old wildtype and FUS Delta14 littermates was homogenised in PBS at 10% w/v, with protease and phosphatase inhibitors (Sigma and Roche Diagnostic, respectively) using a conical-tipped 1ml Teflon-coated homogenizer at 4°C. Equal volume ice old 2% Sarkosyl in PBS was added to lysate and mixed by tituration through a 23G needle. Lysate was centrifuged at 100,000 xg for 30 min at 4C in an ultracentrifuge. Supernatant (soluble fraction) was transferred to a fresh tube and pellet re-suspended in 1ml 1% Sarkosyl in PBS and centrifuged again at 100,000 xg for 30 min at 4C.

Supernatant was discarded and pellet (insoluble fraction) solubilised in 4% SDS. Spinal cord from 120 day old SOD1-G93A mice was used as positive control tissue.

Motor neuron counts

Serial transverse sections (5 µm) from the lumbar region (L3-4) of paraffin-embedded spinal cords were cut and mounted onto sets of 10 polysine-coated glass slides (VWR), such that each slide had 5 sections representing 50 µm intervals within the lumbar spinal cord. This avoids the possibility of counting the same motor neuron in consecutive sections. A total of 5 sets of 10 slides were cut, with one slide from each set being stained with Nissl stain (Cresyl violet acetate, ARCOS Organics) for identification and counting of motor neurons within the ventral horn. Motor neurons were visualised and imaged on a DM2000 LED light microscope (Leica), using LAS version 4.8 software (Leica). Only large, polygonal neurons with a distinguishable nucleus and clearly identifiable Nissl structure were included in the counts.

Immunocytochemistry

Serial transverse sections (5 µm) from the lumbar region (L3-4) of paraffin-embedded spinal cords were dewaxed, followed by antigen retrieval in Tris-buffered saline (pH 9) for 10 minutes in a pressure cooker. Sections were blocked with M.O.M blocking buffer (Vector Labs) then incubated with the following primary antibodies: anti-C-terminal FUS (Novus Biologicals), 1:1000; anti-frameshift FUS, 1:300; anti-S6 riboprotein (CST), 1:500; anti-GFAP (Abcam), 1:250; anti-IBA1 (WAKO), 1:500; anti-p62 (Abcam) 1:1000, anti-Ubiquitin (Santa Cruz) 1:1000. Goat IgG Isotype control (CST) was used as a negative control for anti-frameshift FUS. Sections were then incubated with appropriate secondary antibodies (all Alexa Fluor, Life Technologies, 1:1000). Autofluorescent background was reduced using Sudan black B (0.2% in 70% ethanol/PBS) and sections mounted with ProlongGold containing DAPI (Life Technologies). Images were obtained using an LSM710 META confocal microscope (Zeiss).

Neuromusclular Junction Analysis

Hindlimb lumbrical muscles were dissected and post-fixed in 4% PFA. Free-floating whole muscles were blocked with M.O.M blocking buffer (Vector Labs) then incubated with the following primary antibodies: mouse monoclonal anti-synaptic vesicle glycoprotein 2A

(Developmental Studies Hybridoma Bank), mouse monoclonal anti-neurofilament (165 kDa) (Developmental Studies Hybridoma Bank). M.O.M. biotinylated secondary antibody (Vector Labs) was used following manufacturer instructions. α-Bungarotxin-rhodamine was used to label postsynapses and muscles mounted. Images were acquired on a confocal microscope(Leica DFC 420C) and NMJ were manually counted.

Adult mouse fibroblast preparation

Adult mouse fibroblasts were derived from ear biopsy material of 60-80 day old animals. Briefly, ear biopsies of 1.5 mm diameter were diced with a scalpel and collected in 500 μl collagenase (Sigma) and incubated at 37°C for 25 minutes. The tissue digest was gently agitated and then centrifuged at 1000 rpm for 5 minutes at room temperature. The supernatant was discarded and the cell pellet was washed in 1ml HBSS (Life Technologies), with the pellet resuspend by tituration. The cells were centrifuged again and the supernatant discarded. The cell pellet was then resuspended in 500 μl of 0.25% Trypsin-EDTA (Life Technologies) and incubated for 30 minutes at 37 °C, with gentle trituration every 10 minutes. 500 □l of fresh media was added (DMEM with 10% FBS and 1% penicillin/streptomycin, all from Life Technologies) and the cell suspension centrifuged as before. The supernantant was discarded and the cell pellet resuspended in 5 ml fresh media and transferred to a T-25 flask (Corning) pre-coated with 0.1% gelatin (Millipore) and grown under standard culture conditions of 37°C, 5% CO2 / 95% air.

Human fibroblasts

Muscle biopsy-derived human fibroblasts were obtained from The EuroBioBank and Telethon Network of Genetic Biobanks (GTB12001D to E.P.). All lines with FUS mutations were heterozygous for the mutation and were maintained in DMEM with 10% FBS and 1% penicillin/streptomycin under standard culture conditions.

Induction of stress granules

Both mouse and human fibroblasts were plated onto 13mm glass coverslips pre-coated with 0.1% gelatin (Millipore) at a density of $6x10^3$ cells per coverslip, 24 hours prior to stress granule induction. Stress granules were induced by 90 minute incubation in standard

fibroblast media containing 12 μM thapsigargin (Sigma). Cells were then briefly rinsed with ice-cold PBS (Invitrogen) containing 12 μM thapsigargin and fixed for 10 minutes at RT with ice cold 4% PFA, 4% sucrose in PBS. Fixative was removed from cells with two PBS rinses and cells permeabilised with 0.1% triton X-100 (Sigma) in PBS for 10 minutes. Cells were blocked with 1% BSA (Sigma) in PBS and stress granules were then immuno-fluorescently stained with the following primary antibodies: anti-G3BP (BD biosciences), 1:2000; anti-N-terminal FUS (Novus Biologicals), 1:1000; anti-C-terminal FUS (Novus Biologicals), 1:1000; anti-frameshift FUS, 1:300. Cells were then incubated with appropriate secondary antibodies (all Alexa Fluor, Life Technologies, 1:2000) and coverslips mounted onto glass slides with Prolong Gold containing DAPI (Life Technologies). Images were obtained using a LSM710 META confocal microscope (Zeiss).

RNA sequencing

Snap frozen lumbar spinal cord tissue was pre-incubated in RNAlater-ICE frozen tissue transition solution (Life Technologies) before total RNA was extracted using a miRNeasy mini kit (QIAGEN), as per manufacturer's instructions. RNA was extracted from four male mice of each genotype (wildtype and heterozygous FUS Delta14 littermates) at 3 and 12 months of age. Quality and quantity of extracted RNA was assessed on a 2200 TapeStation (Agilent Technologies). Libraries were prepared using TruSeq Stranded mRNA Library Prep Kits (Illumina), pooled and sequenced on 2 lanes of a HiSeq4000 at 75 bp paired end. Total number of reads was 740 million (per sample: mean 46 million, range 36-54 million). Library preparation and RNA sequencing was carried out by the High-Throughput Genomics Group at the Wellcome Trust Centre for Human Genetics (funded by Wellcome Trust grant reference 090532/Z/09/Z).

Image analysis

All images analysed were obtained using a LSM710 META confocal microscope (Zeiss). All image analysis was carried out using Volocity software (Perkin Elmer). FUS distribution in lumbar spinal motor neurons was measured as follows: wildtype FUS staining was used to define the nucleus and either fluorescent-labelled Nissl stain or S6 riboprotein to define the cytoplasm, using size and intensity thresholds such that only MN with large, clearly defined nuclei were included in the analysis population. Amount of wildtype FUS (comparison

between mice) in each cellular compartment was then calculated as density (amount per area (μm^3)). For mutant FUS the absolute amount of FUS was compared within each MN. For longitudinal analysis a ratio of cytoplasmic to nuclear FUS (both wildtype and mutant FUS) was calculated within each MN (= total cytoplasmic/total nuclear). Global Pearson's Correlation for co-localisation of mutant FUS with RER was calculated using the colocalisation feature of Volocity software (Perkin Elmer), which is designed specifically for quantitative analysis of colocalization as described by Manders et al (1993), Costes et al. (2004) and Barlow et al. (2010). Staining and analysis was carried out on serial sections as used for MN counts (see above). One slide (5 sections) per animal was used in each analysis, allowing 50+ MN to be analysed per animal. N = 5 animals per genotype per time-point.

Bioinformatics analyses

After the trimming of adapters and low quality (phred score < 20) sequence with Trim Galore! 0.4.1 (Martin 2011), RNA-sequencing reads were aligned to the mouse genome mm10 with the STAR aligner v2.4.2a (Dobin, Davis et al. 2013). The aligned reads were sorted and duplicate reads marked with NovoSort 1.03.09 (Novocraft). The mm10 transcript list from Ensembl (Mus_musculus.GRCm38.82) was flattened to create a set of union exons using the dexseq_prepare_annotation.py script included with DEXSeq 1.20.0 (Anders, Reyes et al. 2012). The number of reads overlapping each gene were counted using HTSeq (Anders, Reyes et al. 2012). Differential gene expression between the wildtype and mutant conditions was assessed using DESeq2 1.14.0 (Anders and Huber 2010) running in R 3.3.2. Volcano plots were created using ggplot2 2.1.0 (Wickham 2016). Gene ontology analysis was performed using goseq (Young, Wakefield et al. 2010) using genes in each dataset which passed a relaxed differential expression threshold of unadjusted $P < 0.005$. The p-values for the enrichment test for each GO category were adjusted using the Benjamini-Hochberg false discovery rate at a level of 0.05. Gene Set Enrichment Analysis (GSEA) was performed using the GSEAPreranked tool to calculate enrichment of pathways identified by gene ontology analysis (Subramanian, Tamayo et al. 2005). The gene ranking metric was determined by the inverse p-value assigned a sign based on the direction of the $\log_2$ fold change (Plaisier, Taschereau et al. 2010). Gene sets with $p < 0.05$ and $FDR < 0.25$ were considered significantly changed, as suggested by the GSEA manual.

Statistical analysis

A repeated measures ANOVA test was used to compare between wildtype and heterozygous FUS Delta14 littermates for genotypes, with cage and batch as covariates, across time-points followed by Bonferroni's multiple comparisons testing correction for weight and Locotronic analysis. An ANOVA with cage and batch as covariates, followed by Bonferroni's multiple comparisons testing correction was used to analyse Gait analysis and ECHO-MRI. Kaplan-Meier (LogRank(Mantel-Cox)) survival analysis was used to analyse survival data. The Mann–Whitney U test was used to compare between genotypes for surviving motor units and endplate occupancy for neuromuscular junction analysis. A repeated measure ANOVA without covariates was used to analyse motor neuron counts. Student's t-test was used to analyse wildtype FUS distribution in lumbar motor neurons, p62 and ubiquitin inclusions. Paired t-test was used to analyse distribution of frameshift FUS in cytoplasm versus nucleus of lumbar motor neurons. Two-tailed tests were used in all instances and significance level was set at $P < 0.05$. Global Pearson's Correlation for co-localisation of mutant FUS with RER was calculated using the colocalisation feature of Volocity software (Perkin Elmer), which is designed specifically for quantitative analysis of colocalization as described by Manders et al (1993), Costes et al. (2004) and Barlow et al. (2010).

Compliance with ARRIVE guidelines and ethical standards

All applicable international, national, and institutional guidelines, including ARRIVE guidelines, for the care and use of animals were followed. All procedures performed in studies involving animals were in accordance with the ethical standards of the institution at which the studies were conducted. All experimenters were blind to genotype. All cohorts were of balanced genotype, age and sex, with wildtype littermates as controls. All cages were of mixed genotype, with 3-5 mice per cage. No single housed mice were included in studies. Where appropriate, all statistical analysis had cage and batch as covariates.

All applicable international, national, and institutional ethics and guidelines for the use of human fibroblast cells were followed.
